# Supplementary material for: Effects of dry bio-slurry and nitrogen fertilizer on potato and wheat yields under rotation cropping system
Source: PLoS One. 2024 Jul 31;19(7):e0306625. doi: 10.1371/journal.pone.0306625 (PMC11290689; doi:10.1371/journal.pone.0306625)
Supplement: Attachment — Submitted filename: PONE-D-23-10312_R1_reviewer.pdf [file pone.0306625.s006.pdf]

PLOS ONE

Effects of dry-bio-slurry supplemented with chemical nitrogen fertilizer on growth and yield of potato and wheat under Potato-wheat cropping system

--Manuscript Draft--

**Manuscript Number:**	PONE-D-23-10312R1
**Article Type:**	Research Article
**Full Title:**	Effects of dry-bio-slurry supplemented with chemical nitrogen fertilizer on growth and yield of potato and wheat under Potato-wheat cropping system
**Short Title:**	Effects of dry bioslurry with nitrogen fertilizer on yields of potato and wheat under rotation cropping system
**Corresponding Author:**	Zelalem Addis Musse, Msc<br>Amhara Regional Agricultural Research Institute<br>bahirdar, Amhara ETHIOPIA
**Keywords:**	dry bio-slurry, nitrogen, soil properties, potato, wheat
**Abstract:**	The experiment was conducted to determine the main and residual effect of dry bio-slurry with equivalence N rates on soil properties and yield components of Potato and wheat in the potato-wheat cropping system at Yelmana Denesa district to improve soil fertility through balanced fertilizer application with the objective of optimizing the rate for yield and soil properties. The experiment comprising ten levels of treatments Control (0.0), RN(recommended nitrogen), 50%DBS(dry-bioslurry), 100%DBS(dry-bioslurry), 75%DBS(dry-bioslurry), 75%DBS(dry-bioslurry)+25%RN(recommended nitrogen), 50%DBS(dry bioslurry)+50%RN(recommended nitrogen), 25%DBS(dry-bioslurry)+75%RN(recommended nitrogen), 100%DBS(dry-bioslurry)+25%RN(recommended nitrogen), and 100%DBS(dry-bioslurry)+50%RN (recommended nitrogen) were laid out in RCBD design with three replications for three years (2019-2021). Data on soil Physico-chemical properties and yield components of potato and wheat were collected and subjected to ANOVA using SAS software. The results indicated that the application of different rates of dry bio-slurry with N fertilizer significantly affected the yield components of both potato and wheat. Soil property except pH is not significantly affected by the main effect of dry bio-slurry. Applying 25% DBS with 75 % RN gives the highest tuber yield (27.6 tha-1) compared to the control. Similarly, an application of sole 100% and 75% dry bio-slurry scored the highest grain yield (3.85tha-1) and above-ground biomass (9.59tha-1) respectively due to the residual effect on wheat. Similarly, 25%DBS with 75% RN scored the highest net benefit with acceptable marginal return (above 100%) next to 50%DBS with 50% RN. Generally, the application of dry bio-slurry with nitrogen rates can be improved the yield and yield components of potato and wheat in the study area. To attain the highest net benefit, as DBS within a short period of time application of 50%DBS with 50%RN can be preferable for yield improvement in the study area and similar agro ecological environments. But further research should be done widely and repeatedly in the areas and similar agro –ecologies in permanent plots.
**Order of Authors:**	Zelalem Addis Musse, Msc<br>Tadele Amare Kassie, PhD<br>Bitewelegn kerebeh Kassahun, BSC<br>Antenh Abewa Mogesie, PhD<br>Tesfaye Feysa Beyene, PhD<br>Abrham Awoke Tshale, Bsc<br>Abere Tenagne Molla, Bsc
**Response to Reviewers:**	Reviewer 1<br>.title: It is suggested to rephrase as “Effects of dry-bio-slurry supplemented with chemical nitrogen fertilizer on growth and yield of potato and wheat under Potato-wheat cropping system<br>We have are adjust the title based on reviewer “Effects of dry-bio-slurry supplemented

Powered by Editorial Manager® and ProduXion Manager® from Aries Systems Corporation

	with chemical nitrogen fertilizer on growth and yield of potato and wheat under Potato-wheat cropping system<br>Abstract<br>• It is recommended to include problem statement and objective of the study at the beginning of the abstract.<br>• The conclusion should be changed after correcting the MRR analysis in the results and discussion.<br>Here we also incorporate statement of the problem at the begging of abstract<br>we have tried to check MRR by back tracing of the data its correct but our recommendation and conclusion adjusted from 50%RN+ 50DBS to 25%DBS +75%RN<br>Introduction<br>• The authors should justify why intergraded application of organic and inorganic fertilizer is better than the solo application of the fertilizers.<br>Accepted and incorporated in main document (manuscript)<br>• Previous research experiences in the integrated application of fertilizers should be reviewed.<br>Accepted and incorporated in main document (manuscript)<br>• Most of the information/references are very old and the authors should change them with recent references<br>in case of reference we have tried to explorer recent works and adjust it based on the comment<br>• There are sentences that are difficult to understand and unfinished, which should be revised<br>Accepted and revised in main document (manuscript)<br>• A lot of grammatical and typographical errors that should be revised we also tried adjust the language errors<br>For more information please refer the commented document<br>Methodology<br>This part of the manuscript should be given due attention and requires rigorous revision. Description of the study area, the production system and data collection methods implemented (growth and yield) should be clearly described so that it is understandable and could be repeated by other person (for more information, please refer the attached document).<br>Accepted and incorporated as per the comment in main document (manuscript)<br>Results and discussion<br>The way results are presented is not attractive for the reader. It is suggested to present first the results and then forward why it could be and then compare the results with others.<br>Results should be presented in the past tense form.<br>Although no description of site 1 and site 2 in the methodology, results of site 1 and site 2 (soil sample results) are presented, which are confusing.<br>The economic analysis should be done once again as the MRR of 50%DBS+50%N (8461.1%) is not correct. Accordingly, the conclusion and recommendation part should be revised based on the corrected MRR value.<br>For more information, refer commented document<br>Conclusion and recommendation<br>It should be revised after the revision of the MRR values.<br>All the comments are pertinent, and incorporated in main document (manuscript)<br>References<br>The way reference listed should be uniform and meet the guideline of the journal.<br>Based on journal guide line we adjust it<br>Reviewer #2:<br>I must appreciate authors for nice work in which they have tried to understand supplementation of N with DBS on the performance of Potato-wheat cropping system and soil quality. The study has been conducted by following established procedures and methods and is scientifically sound. The data recorded and presented in the MS are also in order. I havethoroughly gone through the MS and realized that there is still scope to further improve its quality, presentation and content. According some suggestions/comments have been tendered on the MS itself. These comments are easy to understand and self explanatory. In addition to these comments, some specific comments are as under. Authors are advised to revise the MS by duly incorporating all the suggestions/comments.<br><br>1. English language polishing is highly needed to bring the information of the MS in

	presentable form.<br>Accepted and corrected in the main document<br><br>2. Authors are advised to include more yield attributes of both the crops for better and clear understanding of treatment effects on test crops.<br>In addition to the above, it is recommended to include system equivalent yield as study has been attempted in system mode.<br>Not applicable for rotation system<br><br>3. Include recent and updated works to support findings. Authors are also advised to cross check the work cited in text and presented in the reference section and vice versa.<br>Accepted and incorporated<br><br>Reviewer #3:<br>The research holds importance in the area where its being conducted. Although the program is well planned but it needs to be re written. The introduction is very weak, does not fully justify the topic. It needs to be revised keeping in view all the important parameters in the title. The results too are poorly written and discussed with most of the incomplete sentences.<br>the abstract also needs to be rewritten..<br>All are accepted and incorporated in the main document
**Additional Information:**	
**Question**	**Response**
**Financial Disclosure**<br><br>Enter a financial disclosure statement that describes the sources of funding for the work included in this submission. Review the submission guidelines for detailed requirements. View published research articles from *PLOS ONE* for specific examples.<br><br>This statement is required for submission and **will appear in the published article** if the submission is accepted. Please make sure it is accurate.	The research supported by budget by Amhara Agricultural Research Institute (ARARI) and the funder hand no role in data collection and designs and none of the authors had not receive salary from the funder.

**Unfunded studies**<br>Enter: *The author(s) received no specific funding for this work.*<br><br>**Funded studies**<br>Enter a statement with the following details:<br>• Initials of the authors who received each award<br>• Grant numbers awarded to each author<br>• The full name of each funder<br>• URL of each funder website<br>• Did the sponsors or funders play any role in the study design, data collection and analysis, decision to publish, or preparation of the manuscript?<br>• **NO** - Include this sentence at the end of your statement: *The funders had no role in study design, data collection and analysis, decision to publish, or preparation of the manuscript.*<br>• **YES** - Specify the role(s) played.<br><br>* typeset	
**Competing Interests**<br><br>Use the instructions below to enter a competing interest statement for this submission. On behalf of all authors, disclose any competing interests that could be perceived to bias this work—acknowledging all financial support and any other relevant financial or non-financial competing interests.<br><br>This statement is required for submission and **will appear in the published article** if the submission is accepted. Please make sure it is accurate and that any funding sources listed in your Funding Information later in the submission form are also declared in your Financial Disclosure statement.<br><br>View published research articles from *PLOS ONE* for specific examples.	The authors have declared that no competing interests exist.

**NO authors have competing interests**<br><br>Enter: *The authors have declared that no competing interests exist.*<br><br>**Authors with competing interests**<br><br>Enter competing interest details beginning with this statement:<br><br>*I have read the journal's policy and the authors of this manuscript have the following competing interests: [insert competing interests here]*<br><br>* typeset	
**Ethics Statement**<br><br>Enter an ethics statement for this submission. This statement is required if the study involved:<br><br>• Human participants<br>• Human specimens or tissue<br>• Vertebrate animals or cephalopods<br>• Vertebrate embryos or tissues<br>• Field research<br><br>Write "N/A" if the submission does not require an ethics statement.<br><br>General guidance is provided below. Consult the submission guidelines for detailed instructions. **Make sure that all information entered here is included in the Methods section of the manuscript.**	experiment is field research.

Powered by Editorial Manager® and ProduXion Manager® from Aries Systems Corporation

**Format for specific study types**<br><br>**Human Subject Research (involving human participants and/or tissue)**<br>• Give the name of the institutional review board or ethics committee that approved the study<br>• Include the approval number and/or a statement indicating approval of this research<br>• Indicate the form of consent obtained (written/oral) or the reason that consent was not obtained (e.g. the data were analyzed anonymously)<br><br>**Animal Research (involving vertebrate animals, embryos or tissues)**<br>• Provide the name of the Institutional Animal Care and Use Committee (IACUC) or other relevant ethics board that reviewed the study protocol, and indicate whether they approved this research or granted a formal waiver of ethical approval<br>• Include an approval number if one was obtained<br>• If the study involved *non-human primates*, add *additional details* about animal welfare and steps taken to ameliorate suffering<br>• If anesthesia, euthanasia, or any kind of animal sacrifice is part of the study, include briefly which substances and/or methods were applied<br><br>**Field Research**<br><br>Include the following details if this study involves the collection of plant, animal, or other materials from a natural setting:<br>• Field permit number<br>• Name of the institution or relevant body that granted permission	
**Data Availability**<br><br>Authors are required to make all data underlying the findings described fully available, without restriction, and from the time of publication. PLOS allows rare exceptions to address legal and ethical concerns. See the PLOS Data Policy and FAQ for detailed information.	Yes - all data are fully available without restriction

A Data Availability Statement describing where the data can be found is required at submission. Your answers to this question constitute the Data Availability Statement and **will be published in the article**, if accepted.<br><br>**Important:** Stating 'data available on request from the author' is not sufficient. If your data are only available upon request, select 'No' for the first question and explain your exceptional situation in the text box.<br><br>Do the authors confirm that all data underlying the findings described in their manuscript are fully available without restriction?	
**Describe where the data may be found in full sentences. If you are copying our sample text, replace any instances of XXX with the appropriate details.**<br><br>• If the data are **held or will be held in a public repository**, include URLs, accession numbers or DOIs. If this information will only be available after acceptance, indicate this by ticking the box below. For example: *All XXX files are available from the XXX database (accession number(s) XXX, XXX.*).<br>• If the data are all contained **within the manuscript and/or Supporting Information files**, enter the following: *All relevant data are within the manuscript and its Supporting Information files.*<br>• If neither of these applies but you are able to provide **details of access elsewhere**, with or without limitations, please do so. For example:<br><br>*Data cannot be shared publicly because of [XXX]. Data are available from the XXX Institutional Data Access / Ethics Committee (contact via XXX) for researchers who meet the criteria for access to confidential data.*<br><br>*The data underlying the results presented in the study are available from (include the name of the third party*	all nessary dats avilable in the manuscript

and contact information or URL).<br>• This text is appropriate if the data are owned by a third party and authors do not have permission to share the data.<br><br>* typeset	
Additional data availability information:	Tick here if your circumstances are not covered by the questions above and you need the journal's help to make your data available.

Powered by Editorial Manager® and ProduXion Manager® from Aries Systems Corporation

Click here to access/download;Manuscript;reviewed plose one Manuscript.docx

Effects of dry-bio-slurry supplemented with chemical nitrogen fertilizer on growth and yield of potato and wheat under Potato-wheat cropping system

Zelalem Addis*[1],Tadele Amare[1], Bitewlgn Kerebih[1], Antenh Abewa[1], Tesfaye Feysa[2] ,Abrham Awoke[1] and Abere Tenagne[1]

1 Adet Agricultural Research Center P.O.BOX 08, Bahir dar Ethiopia

2 Amhara Agricultural Research Institute

*Corresponding Author zelalemaddis660@gmail.com

ABSTRACT

The experiment was conducted to determine the main and residual effect of dry bio-slurry with equivalence N rates on soil properties and yield components of Potato and wheat in the potato-wheat cropping system at Yelmana Denesa district to improve soil fertility through balanced fertilizer application with the objective of optimizing the rate for yield and soil properties. The experiment comprising ten levels of treatments Control (0.0), RN(recommended nitrogen), 50%DBS(dry-bioslurry), 100%DBS(dry-bioslurry), 75%DBS(dry-bioslurry), 75%DBS(dry-bioslurry)+25%RN(recommended nitrogen), 50%DBS(dry-bioslurry)+50%RN(recommended nitrogen), 25%DBS(dry-bioslurry)+75%RN(recommended nitrogen), 100%DBS(dry-bioslurry)+25%RN(recommended nitrogen), and 100%DBS(dry-bioslurry)+50%RN (recommended nitrogen) were laid out in RCBD design with three replications for three years (2019-2021). Data on soil Physico-chemical properties and yield components of potato and wheat were collected and subjected to ANOVA using SAS software. The results indicated that the application of different rates of dry bio-slurry with N fertilizer significantly affected the yield components of both potato and wheat. Soil property except pH is not significantly affected by the main effect of dry bio-slurry. Applying 25% DBS with 75 % RN gives the highest tuber yield

(27.6 tha-1) compared to the control. Similarly, an application of sole 100% and 75% dry bio-slurry scored the highest grain yield (3.85tha^{-1}) and above-ground biomass (9.59tha^{-1}) respectively due to the residual effect on wheat. Similarly, 25%DBS with 75% RN scored the highest net benefit with acceptable marginal return (above 100%) next to 50%DBS with 50% RN. Generally, the application of dry bio-slurry with nitrogen rates can be improved the yield and yield components of potato and wheat in the study area. To attain the highest net benefit, as DBS within a short period of time application of 50%DBS with 50%RN can be preferable for yield improvement in the study area and similar agro ecological environments. But further research should be done widely and repeatedly in the areas and similar agro –ecologies in permanent plots.

Key Words: dry bio-slurry, nitrogen, soil properties, potato, wheat

1. Introduction

A well planned cropping system can enhance the efficiency of nutrient use, reduce the need for fertilizers and lessen the impact of pests and diseases (1&2). Potato and wheat production under a crop rotation system in the region of Amhara, Ethiopia is commonly practiced by farmers. These raw materials are intended for human and animal consumption. The system comprises a cradle-to-farm analysis for 3-years rotation cycle. It allows mitigating the impact of certain pests and diseases, which significantly affects the yield of the main crop: the potato. In addition to this, it is possible to take full advantage of the residual fertility that the potato leaves in the soil (a crop that is usually abundantly fertilized). This rotation system for potato and wheat crops was chosen by many producers as it is considered the most efficient from an economic, quality and soil health point of view.Potato (*Solanum tuberosum*) is the world's third most important food crop after wheat and rice (3). In Ethiopia, the area under potato cultivation reaches about

73,677.64 ha and its production was 1,044,436.359 tons (4). The productivity of potato in Ethiopia is 13.9 t ha^{-1}(5), which is relatively low compared to other African countries (6). Bread wheat (*Triticum aestivum* L.) is also is one of the major cereal crops grown in the highlands of Ethiopia, and the country is regarded as the largest wheat producer in Sub-Saharan Africa (7).

Out of the total grain crop area, wheat ranked 4th after tef (Eragrostis tef), maize (Zea mays) and sorghum (Sorghum bicolor), while third in total production after maize and tef (5). Despite the long history of wheat cultivation and its importance to Ethiopian agriculture, its average yield is still very low, not exceeding 2.4 t ha^{-1} (5) which is below the world's average of 3.4 t ha^{-1} (6) this phenomenon is common in our region. Low level of potato-wheat productivity is mainly due to soil fertility degradation, improper fertilization, poor pest management practices, use of the low-quality seed, and soil nutrient depletion (8).Enhancing fertility is a precondition for a practical crop production system through organic manure like bio-slurry that can achieve sustainability soil fertility and crop production (9&10). The bio-slurry that obtained after extraction of the energy content of animal manure is an excellent fertilizer, rich in major nutrients (nitrogen, phosphorous and potassium) and organic matter that determine the soil fertility and yield of different crops and vegetables (11). It also improves the physical and biological quality of soil besides providing both macro and micro-nutrients to crops and vegetables. The application of bio-slurry also helps in the reduction of dependence on mineral fertilizers (12). Because both Potato and wheat are highly responsive to N fertilization; it is usually the most limiting essential nutrient for growth and development of those crops (13).In addition, supplying Nitrogen plays an important role in the balance between vegetative and reproductive growth for both crops (14&15). Various studies have shown that N fertilizer applications can increase the dry matter-

protein content of wheat and potato tubers (16). Furthermore, most of the time the available nitrogen in most organic source fertilizers including bio-slurry is high as compared to other nutrients in concentration (11) Due to this dry bio-slurry and nitrogen fertilizers were applied through nitrogen equivalence.Besside this Neither organic manure nor Chemical fertilizer alone can meet the nutrient demand of a given crop because their own negative impact in terms of sustainability for crops in the different cropping system via soil-plant interaction (17).For instance continuous addition of chemical fertilizers can cause soil quality deterioration such as increasing of soil acidity, loss of organic matter and depletion of nutrients that are not supplied in the fertilizer formulation (43). On the other hand organic fertilizers are required in large quantities and there nutrients released slowly and less effect on soil properties and crop yield within short period of time as compared to inorganic fertilizers (44). Because of this, the study was done to determine the main and residual integrated effects of dry-bio slurry and nitrogen fertilizer on potato–wheat cropping system (potato and wheat rotation) for production and soil physic-chemical properties at Yilemana Densa district with flowing specific objectives to determine the optimum rate of dried bio-slurry and nitrogen fertilizer on potato and wheat yield under potato –wheat cropping system and soil Physico-chemical properties improvement in northwestern Amhara region Ethiopia

2. Material and methods

2.1 Description of Study Area

The experiment was conducted at Yilemana Densa district on Farmer's field across three sites (Y1S1,Y2S1and Y2S2) for three consecutive years (2019-2022) as potato and wheat rotation (potato-wheat cropping system) at Debremewi Keble West Gojam Zone North Western Amhara Region of Ethiopia. Geographically the area lies at 11^0 21' 22" N and 37^0 25' 43" E (Figure 1)

with a mean altitude of 2304 m above sea level. It receives a mean annual rainfall of 1421 mm with mean minimum and maximum temperatures of 12.29 and 27.56°C, respectively. The landforms of the area are characterized by undulating to rolling plateaus, scattered moderate hills, dissected side slopes, and river gorges (18). Based on the district Bureau of Agriculture, the major land use comprises cultivated land (57%), forest and bushes (2%), grazing land (33%), and others (8%). Major crops, grown in the study area are Maize, Tef, wheat, barley, potato, and field pea which take the lion's share. Soil types in the area are Nitisols (45%) Vertisols (30%) and Luvisols (25%). This on-farm experiment was conducted on Luvic Nitisols which is the most dominant soil in the study area

2.2 Experimental procedure

The experiment was contain ten treatments which includes control (without N), recommended N (138N),dry-bio slurry 50% equivalence (5.3tha^{-1}),dry-bio slurry 100% equivalence (10.6 tha^{-1}), dry-bio slurry75%equivalence(7.95 tha^{-1}),dry-bio slurry75% equivalence (7.95tha^{-1})+25% recommended N (34.5N), dry-bio slurry 50% equivalence (5.3tha^{-1})+50% recommended N (69N),dry-bio slurry 25% equivalence (2.65tha^{-1})+75% recommended N(103.5N),dry-bio slurry100% equivalence (10.6tha^{-1})+25% recommended N(34.5N) and dry-bio slurry 100% equivalence (10.6 tha^{-1}) +50% recommended N (69 N) that laid out in Randomized Complete Block Design (RCBD) with three replications. The rates of dry-bio slurry were adjusted based on potato recommended rate 138 kg N equivalency corresponding to its N content. Urea was used as a source of synthetic 138N whereas 69P_2O_5 was applied in the form of TSP to all plots. The experiment was carried out under rain-fed conditions *Gudenie* and TAY varieties were used as test crops for Potato and wheat respectively in each rainy season one after the other. Total area of each plot was 4.5 m x 3 m (13.5 m^2) having 1m space between plots and 1.5m between blocks.

The potato was spaced 0.3m between plants and each plot consisted of six rows at 0.75m intervals and the data were collected from the middle four rows. Wheat was planted as a rotated crop in 0.2m and harvested from middle rows by avoiding four rows as a border.

2.2.1 Dry bio-slurry

It was collected from farmers biogas plants and stored under well protected shade for better drying and avoid nutrient loss especially nitrogen. After it drying make fine and breake int small pices for nice application and soil reaction. After this during planting time this well prepared dry-bio slurry was incorporated into the soil at planting period time. Representative composite sample dry-bioslurry was taken from the whole collected dried pit of slurry that was collected from different biogas plants across individual farmers for analysis of (pH), organic carbon (OC%), cation exchange capacity (CEC), total nitrogen (TN%) and available phosphorus (P) by following laboratory procedures (19).

Table 1. Physico-chemical analysis of dry-bio slurry before incorporation in year1and 2 and its average

	Dry bio-slurry	Concentration
**Year 1**		
	Dry matter%	11.2
	OC%	27.8
	TN%	1.2
	Av P (mg kg^{-1})	112.1
	pH (H_2O;1:2.5)	7.9
	C:N ratio	22.9
	CEC (cmol kg^{-1})	59.8
**Year 2**		
	Dry matter%	11.8
	OC%	16.3
	TN%	1.4

Av P (mg kg^{-1})	91.6
pH (H_2O;1:2.5)	7.7
C:N ratio	11.6
CEC (cmol kg^{-1})	67.4
**Average result of Year1andYear 2**	
Dry matter%	11.5
OC%	22.1
TN%	1.3
Av P (mg kg^{-1})	101.9
pH (H_2O;1:2.5)	7.8
C: N ratio	17.3
CEC (cmol kg^{-1})	63.6

OC%=organic carbon percent, TN%=total nitrogen percent, C: N=carbon to nitrogen ratio, CEC = cation exchange capacity, AvP=available phosphorus, and pH= Power of hydrogen concentration.

2.2.2Gudenie potato variety

This varieties is used as attest crop for potato and it was planted as main crop or precursor crop of wheat

2.2.3TAY variety of Wheat

It was planted in the next cropping season after potato which was tested for the determination of dry bioslurry residual effect in potato-wheat rotation cropping system . P_2O_5 was applied during the planting period of time as basal whereas inorganic N for potato was applied in three; one-third third at planting, one-third about 30 days after planting, and the remaining one-third at the beginning of the flowering. While for wheat recommended NP was applied in all plots except control which only received P_2O_5 to see the residual effect of dry bio-slurry in the next crop and nitrogen was supplied, in two split.

2.3 Data Collection, Preparation, and Analysis

2.3.1 soil sampling and analysis before planting

In each every year before planting, representative soil samples were collected from 0-20 cm depth in a random sampling method from 10 spots in the field by using an auger. All samples were mixed together and one composite sample was formed. The composite sample was grounded using a mortar and passed through a 2 mm sieve for analysis of soil texture, CEC, pH, and available P; whereas a 0.5 mm sieve was used for determining the organic carbon (OC) and total N. Bulk density was determined by the core sampling method.

2.3.2 Soil sampling and analysis after harvest in potato–wheat cropping system

The major chemical properties of soil such as OC, pH, CEC, total N and available P were analyzed following the compiled laboratory manual of (19). Soil pH was measured in water at the ratio of 1:2.5 using glass electrode pH meter. The soil OC content was determined following the wet digestion method as outlined by Walkley and Black which involves the digestion of OC in the soil samples with potassium dichromate ($K_2Cr_2O_7$) in a sulphuric acid solution. AvP was determined by Olsen extracting method. The total N content in the soil samples was determined following the Kjeldahl method. CEC was determined by extracting the soil samples with ammonium acetate ($1NNH_4OAc$) followed by repeated washing with ethanol (96%) to remove the excess ammonium ions in the soil solution. Percolating the $NH4^+$ saturated soil with sodium chloride would displace the ammonium ions adsorbed in the soil and the ammonium liberated from the distillation was titrated using 0.1N NaOH.

Crop data collection

Plant height was measured for both potato and wheat at the maturity stage by taking five randomly selected plants from ground to the top apex and averaging for a single reading.

Similarly spike length was measured for wheat at the maturity stage by taking five randomly selected plants from staring point of spike grain to the end of spike grain and averaging for a single reading. While number of plants per hill (stem number per hill) was cllected by counting number stems from five hill of potato at 70% maturity stage and averaging for mean reading

The number of tubers per plant potato was taken by counting tubers from five randomly taken plants at the maturity stage from the middle six rows of experimental plots ($9m^2$) and averaged for a single estimation of the mean value. While thousand seed weight for wheat was taken from each treatment hrevestable plot ($11.7m^2$) by counting of 1000 seeds at maturity stage after tershing and make them weighting under sensitive balance for weight reading . Total tuber yield of potato was measured at harvesting by taking all tubers from the middle six rows ($9m^2$) plot area and weighted through balancing balance for total weight reading via avoiding of border effects. Grain yield, and above-ground biomass of wheat was taking at maturity stage from the net harvestable plot($11.7m^2$) area and both of the exposed for balancing balance for measuring and reading weight

2.4 Economic Analysis

Economic analysis was performed to make rational choice among the applied variables in the production of potato. The partial budget and marginal rate of return (MRR) were used for evaluating the change in farming methods that affect partially rather than the whole farm practice and also concerned with planning tool to estimate the profit change within a farm (CIMMYT, 1988). This was computed by adjusting yield downward by a 10% and multiplying it with the local field price (6 Ethiopian Birr per kg of potato). Dominance analysis was done by listing of treatments in an increasing order of cost and that has net benefit less than or equal to treatments with the lower costs that vary is dominated (20).

2.5 Statistical Analysis

All data were subjected to analysis of variance through GLM procedure by using SAS software program version 9.4 (SAS Institute, 2002). List significant difference (LSD) at 0.05 probability level was employed to differentiate treatments means t (21).

3. Results and discussion

3.1 Soil Chemical Properties before planting and After Harvesting of potato

The results of soil chemical properties before and after harvest from each experimental site are indicated in Table 2, 3 & 4. Before-planting soil was acidic in reaction with a pH (H_2O 1:2.5) value of 5.1Y1S1(year one site one) and 5.2Y2S1(year two site one), which is within the range of soil pH for potato production (22) (table 2). The total N, available P, OC, C: N ratio and CEC of the soil Y1S1 and Y2S1 before planting were (0.16&0.16%), (6.9&6.3 mg kg^{-1}), (1.8 &1.5%),(11.1&9.3) and (33.9&30.3cmol (+) kg^{-1}), respectively (Table 2). The total N content of the soil was within the range of medium according to (22) who classified the range of total N < 0.1, 0.1- 0.15, 0.15-0.25 and > 0.25% as very low, low, medium and high, respectively. (23) classified available P content of the range < 5 as very low, 5 -15 as low, 15 -25 as medium and > 25 mg kg^{-1} as high. Hence the available P of the soil before planting lies under the low range. According to (24) the soil OC content ranges of 1-2, 2-4, and 4-6% are rated as low, medium and high, respectively while cation exchange capacity (CEC) ranges of 5-15, 15-25 and 25-40 cmol kg^{-1} are rated as low, medium and high, respectively.

Based on these ratings the OC (1.4, 1.5 &2.0%) and CEC (33.9, 30.3&30.9 cmol kg^{-1}) before planting of the experimental fields of (Y1S1,Y2S1 and Y2S2) were in the low and high ranges, respectively. Generally, the nutrient contents of the study site Y2S1 is not good in terms of

availability of major plant nutrients beside its nice CEC. On the other hand on Y2S2 pH, TN, avP, OC, C: N and CEC pre planting values were 5.5, 0.17%, 6.8ppm, 2.4%, 14.1 and 33.9 respectively. Based on (22) rating the pH value was under moderate while total nitrogen was medium. Similarly, according to (23) available P was under low while OC and CEC were medium and high respectively (24). Y2S2 rather than Y1S1and Y2S1 have good soil fertility status based on its values of soil chemical properties. However, the acidity of soil Y1S1andY2S2 may be cause the sorption of available P. Thus, application of OM like bio-slurry is very essential in order to neutralize soil solution for the availability of nutrients. On the other hands, after harvest all soil chemical properties except soil pH (on Y2S2) were not affected by the application of dry bio-slurry with N equivalence rates (Table3).This might be hydrogen replacement through substitution reaction of basic cations from dry bio slurry in soil solution or rhizosphere

The non-significant effects of the applied treatments on these parameters might be due to the slow release of nutrients from dry bio-slurry that was applied during the experimentation period to soil solution related to short cropping season potato cultivation. However, applied dry bio-slurry and N rates had a significant ($P < 0.05$) effect on pH at site2 in 2020-21soil after harvest as compared to the control (Table 2). Numerically the highest soil pH content was obtained by the application of 100% and 100%+25%N dry bio-slurry as compared to the control (Table 3). The increment of soil pH in treated plots may be related due to the releasing of basic cations from dry bio-slurry into soil solution that makes substitute of acid cations.These results were in agreement with the investigation of (25) they reported that the combined application of compost with inorganic NPSB in maize increased the soil pH content after harvest as compared to control.

3.2 Residual effect of dry bio-slurry on soil chemical properties after harvesting of wheat

The residual effect of dry bio-slurry on selected soil chemical properties was significantly explained at ($p<0.05$) across sites and years in table 4. Related to this an addition of 100% (10.6tha^{-1}) dry bio-slurry gives the highest TN% (0.207and 0.223) on Y1S1 and YS2 respectively than the control. This might be due to the gradual releasing of Nitrogen dry bios-slurry into soil solution beyond its chelating capacity. The finding lined with (26)they found that an application of dry bio-slurry with inorganic fertilizers gives the maximum value of total nitrogen than control. Similarly, the study conducted by (27) also indicated that an application of 70cm^3 scored the highest (1.36%) TN as compared to the control which gives 0.07%

On the other hand, OC, C: N and CECwere significantly affected at Y2S1(Table 4) While in Y2S2 only CEC is significant at ($p<0.05$) Table 4.Based on this numerically the highest value of CEC(34.6), C: N (12.82)&OC% (2.20) was observed by application 50%DBS and 100% DBS with 25%N as compared to control on Y2S1.On Y2S2 the maximum value of CEC(cmolkg^{-1}) 36.06 was obtained in plots that receive 100%DBS with 25%N than control. This might be due to the positive effect applied to DBS that enables increasing organic matter and holding capacity of positive cation in the soil exchangeable site. The finding agreed with (28) who revealed that an application of 41.3m^{-3} liquid bio-slurry with 20.5kgha^{-1}N significantly increased soil organic carbon than untreated plots. The study conducted by (29) also indicated that an application of 10tha^{-1} with 50%Nkgha^{-1} significantly increased OC% by scoring the maximum value of 0.67 than the control. Organic amendments significantly enhanced SOC thus had a considerable effect on soil microbes and nutrient availability and uptake this may alter the C: N ratio. However, the addition of external organic matter with a low C: N ratio may induce the mineralization of OM. This makes nitrogen trapped by organic matter; a phenomenon known as the priming effect (30). The application of the 50% DBS and 100% DBS with 25%N gives the highest CEC at Y2S1

AND Y2S2 respectively as compared to the control (Table 3) Such increment in CEC might be due to the application of DBS on soil that makes a negatively charged colloidal site and storehouse of basic cations. The finding agreed with (31) who reported that the use of organic FYM and inorganic fertilizers significantly increased CEC over the control.

Table 2.intial soil properties of the experimental sites

				**2019-20 (Y1S1)**			
**Texture**	BD	pH	TN%	Av P (ppm)	CEC (cmol kg^{-1})	C:N	OC%
**SCL**	1.22	5.1	0.16	6.9	33.9	8.8	1.4
				**2020-21 (Y2S1)**			
**Texture**	BD	pH	TN%	Av P (ppm)	CEC (cmol kg-1)	C:N	OC%
**SCL**	1.33	5.2	0.16	6.3	30.3	9.3	1.5
				**2020-21 (Y2S2)**			
**Texture**	BD	pH	TN%	Av P (ppm)	CEC (cmol kg-1)	C:N	OC%
**SCL**	1.26	5.5	0.17	6.8	33.9	11.8	2.0

NB: SCL= sandy clay loam; BD= bulk density Table 3.main effect of dry bio slurry with nitrogen on soil chemical properties after harvest of potato

			**2019-20 (Y1S1)**			
**Treatment**	pH	TN%	Av P (ppm)	CEC (cmol kg^{-1})	C:N	OC%
Control (0,0)	5.13	0.12	9.3	29.1	11.6	1.4
RN (138Nkgha^{-1})	5.11	0.12	7.8	29.9	11.61	1.3
50% DBS (5.3tha^{-1})	5.19	0.13	8.8	29.6	11.59	1.4
100% DBS (10.6tha^{-1})	5.18	0.13	9.6	29.7	11.58	1.5
75%DBS(7.95tha^{-1})	5.19	0.13	10.2	29.1	11.62	1.5
75%DBS+25%N (7.95tha^{-1}+34.5Nkgha^{-1})	5.22	0.16	10.1	28.3	11.57	1.9
50%DBS+50%N (5.3tha^{-1}+69Nkgha^{-1})	5.10	0.15	9.1	28.7	11.59	1.7

25%DBS+75%N (2.65tha^{-1}+103.5Nkgha^{-1})	5.18	0.15	7.5	29.2	11.61	1.7
100%DBS+25%N (10.6tha^{-1}+34.5Nkgha^{-1})	5.12	0.13	12.3	28.3	11.62	1.5
100%DBS+50%N (10.6tha^{-1}+69Nkgha^{-1})	5.15	0.13	11.2	27.6	11.60	1.5
**LSD**	**NS**	**NS**	**NS**	**NS**	**NS**	**NS**
**CV%**	**1.2**	**14.3**	**22.43**	**3.3**	**0.21**	**14.19**
			**2020-21 (Y2S1)**			
**Treatment**	pH	TN%	Av P (ppm)	CEC (cmol kg^{-1})	C:N	OC%
Control (0,0)	5.2	0.15	4.4	27.3	11.59	1.7
RN (138Nkgha^{-1})	5.3	0.16	4.3	27.7	11.59	1.8
50% DBS (5.3tha^{-1})	5.3	0.14	6.5	30.8	11.61	1.7
100% DBS (10.6tha^{-1})	5.4	0.15	6.2	27.4	11.60	1.8
75%DBS(7.95tha^{-1})	5.4	0.15	5.8	28.6	11.58	1.7
75%DBS+25%N (7.95tha^{-1}+34.5Nkgha^{-1})	5.3	0.14	5.4	24.4	11.63	1.7
50%DBS+50%N (5.3tha^{-1}+69Nkgha^{-1})	5.3	0.14	4.5	26.7	11.61	1.6
25%DBS+75%N (2.65tha^{-1}+103.5Nkgha^{-1})	5.2	0.14	4.6	27.5	11.59	1.7
100%DBS+25%N (10.6tha^{-1}+34.5Nkgha^{-1})	5.4	0.17	4.4	28.4	11.59	1.9
100%DBS+50%N (10.6tha^{-1}+69Nkgha^{-1})	5.4	0.16	4.4	28.5	11.61	1.9
**LSD**	**NS**	**NS**	**NS**	**NS**	**NS**	**NS**
**CV%**	**2.12**	**13.5**	**21.6**	**9.0**	**0.17**	**13.5**
			**2020-21 (Y2S2)**			
**Treatment**	pH	TN%	Av P (ppm)	CEC (cmol kg^{-1})	C:N	OC%
Control (0,0)	5.3^{cd}	0.17	12.2	30.6	12.10	2.1

RN (138Nkgha^{-1})	5.4b^{cd}	0.17	7.1	31.9	11.60	1.9
50% DBS (5.3tha^{-1})	5.5^{abc}	0.18	11.5	30.4	11.60	2.0
100% DBS (10.6tha^{-1})	5.6^{ab}	0.18	9.5	32.1	12.62	2.3
75%DBS(7.95tha^{-1})	5.6^{ab}	0.17	13.2	32.1	12.00	2.0
75%DBS+25%N (7.95tha^{-1}+34.5Nkgha^{-1})	5.5^{abc}	0.18	13.3	33.5	11.60	2.1
50%DBS+50%N (5.3tha^{-1}+69Nkgha^{-1})	5.5^{abcd}	0.17	13.1	32.6	12.01	2.1
25%DBS+75%N (2.65tha^{-1}+103.5Nkgha^{-1})	5.3^{d}	0.16	9.5	30.9	11.60	1.9
100%DBS+25%N (10.6tha^{-1}+34.5Nkgha^{-1})	5.6^{a}	0.18	11.6	31.8	11.80	2.1
100%DBS+50%N (10.6tha^{-1}+69Nkgha^{-1})	5.4^{bcd}	0.17	15.3	33.4	11.60	2.1
**LSD**	**2.3**	**NS**	**NS**	**NS**	**NS**	**NS**
**CV%**	**0.22**	**9.9**	**29.1**	**6.1**	**5.9**	**13.8**

Means followed by the same letter (s) within the column are not significantly different at ($P \leq 0.05$). control = is no nitrogen and dry bioslurry,RNP=percent of recommended nitrogen and phosphorus, DBS =dry bio-slurry, pH= power of hydrogen concentration, TN% = total nitrogen percent, AvP = available phosphorus, OC% = organic carbon percent, C: N ratio = carbon to nitrogen ratio, CEC = cation exchange capacity, OM% = organic matter percent, Y1S1= year one site one, Y2S1= year two site one and Y2S2 = year two site two.

Table 4. The residual effect of dry bio-slurry on soil chemical properties after wheat harvesting in the potato-wheat cropping system

**2020-21 (Y1S1)**						
Treatment	pH	TN%	Av P (ppm)	CEC (cmol kg^{-1})	C:N	OC%
Control (0,0)	5.40	0.174bcd	8.97	21.39	9.68	1.69
RN (138Nkgha^{-1})	5.40	0.196a	10.12	21.69	10.01	1.95
50% DBS (5.3tha^{-1})	5.40	0.197a	9.25	26.13	9.64	1.88

100% DBS (10.6tha^{-1})	5.38	0.168d	11.04	23.51	11.81	1.98
75%DBS(7.95tha^{-1})	5.40	0.192ab	9.34	25.60	9.42	1.82
75%DBS+25%N (7.95tha^{-1}+34.5Nkgha^{-1})	5.42	0.172cd	9.07	24.51	11.98	2.06
50%DBS+50%N (5.3tha^{-1}+69Nkgha^{-1})	5.36	0.188abc	10.06	22.09	9.61	1.80
25%DBS+75%N (2.65tha^{-1}+103.5Nkgha^{-1})	5.35	0.202a	10.08	25.11	10.14	2.04
100%DBS+25%N (10.6tha^{-1}+34.5Nkgha^{-1})	5.31	0.194ab	10.46	21.81	11.01	2.13
100%DBS+50%N (10.6tha^{-1}+69Nkgha^{-1})	5.37	0.205a	10/76	23.78	9.35	1.89
**LSD**	NS	0,020	NS	NS	NS	NS
**CV%**	1.3	6.2	12.7	11.9	14.8	13.3

2021-22 (Y2S1)

Treatment	pH	TN%	Av P (ppm)	CEC (cmol kg^{-1})	C:N	OC%
Control (0,0)	5.61	0.178	10.15	27.70d	7.61d	1.36b
RN (138Nkgha^{-1})	5.53	0.179	10.22	30.52bc	10.31abc	1.85a
50% DBS (5.3tha^{-1})	5.69	0.180	11.56	34.67a	12.25abc	2.20a
100% DBS (10.6tha^{-1})	5.67	0.160	9.85	31.77bc	12.42ab	1.98a
75%DBS(7.95tha^{-1})	5.64	0.194	9.85	31.09bc	9.74cd	1.88a
75%DBS+25%N (7.95tha^{-1}+34.5Nkgha^{-1})	5.70	0.178	11.76	29.56cd	10.79abc	1.90a
50%DBS+50%N (5.3tha^{-1}+69Nkgha^{-1})	5.62	0.167	10.69	30.63bc	11.38abc	1.90a
25%DBS+75%N (2.65tha^{-1}+103.5Nkgha^{-1})	5.54	0.186	9.59	30.37bc	10.00bcd	1.86a
100%DBS+25%N (10.6tha^{-1}+34.5Nkgha^{-1})	5.58	0.172	9.88	31.99bc	12.82a	2.20a
100%DBS+50%N (10.6tha^{-1}+69Nkgha^{-1})	5.44	0.185	10.19	32.91ab	11.78abc	2.17a

**LSD**	NS	NS	NS	2.60	2.61	0.41
**CV%**	2.0	6.7	14.4	4.8	14.1	12.6
			**2021-22 (Y2S2)**			
Treatment	pH	TN%	Av P (ppm)	CEC (cmol kg^{-1})	C:N	OC%
Control (0,0)	5.51	0.176de	10.03	29.43c	12.03	2.12
RN (138Nkgha^{-1})	5.65	0.190bcd	8.76	30.69bc	12.19	2.29
50% DBS (5.3tha^{-1})	5.62	0.186bcde	13.49	35.92 a	12.78	2.38
100% DBS (10.6tha^{-1})	5.62	0.209ab	14.08	33.68ab	11.63	2.43
75%DBS(7.95tha^{-1})	5.76	0.178cde	11.74	34.47a	13.41	2.39
75%DBS+25%N (7.95tha^{-1}+34.5Nkgha^{-1})	5.68	0.207ab	12.93	34.81a	12.29	2.54
50%DBS+50%N (5.3tha^{-1}+69Nkgha^{-1})	5.57	0.202abc	13.08	35.88a	11.47	2.30
25%DBS+75%N (2.65tha^{-1}+103.5Nkgha^{-1})	5.59	0.164e	11.07	33.27ab	13.52	2.19
100%DBS+25%N (10.6tha^{-1}+34.5Nkgha^{-1})	5.66	0.223a	11.51	36.06a	10.09	2.24
100%DBS+50%N (10.6tha^{-1}+69Nkgha^{-1})	5.66	0.207ab	11.21	35.95a	11.75	2.42
**LSD**	NS	0.025	NS	3.78	NS	NS
**CV%**	2.3	7.6	17.8	6.5	10.5	7.3

Means followed by the same letter (s) within the column are not significantly different at ($P \leq 0.05$). RNP=percent of recommended nitrogen and phosphorus, DBS =dry bio-slurry, pH= power of hydrogen concentration, TN% = total nitrogen percent, AvP = available phosphorus, OC% = organic carbon percent, C: N ratio = carbon to nitrogen ratio, CEC = cation exchange capacity, OM% = organic matter percent, Y1S1= year one site one, Y2S1= year two site one and Y2S2 = year two site two.

3.2 Effects of dry bio slurry on potato and wheat yield and yield attributers in the potato-wheat cropping system

3.2.1 Plant height and number of Stem per hill of potato

Integration of dry bio slurry with N rates was significantly ($P < 0.05$) affects plant height (Table 5). Numerically,the highest values of plant height (52.1 and 48.8cm) was achieved with addition of RNP and 25%DBS with 75%N recpectively as compare control and 50%DBS that gives minimum values (Table 5).Increasing of plant height in response to application of DBS with N fertilizer may be due to the improvement of physico- chemical properties of the soil that resulted increased water absorption and nutrient utilization of the plant.Moreover, application of DBS may deliver balanced micro and macronutrients as well as enhanced availability of plant nutrients,which would help to enhance the metabolic activity of microorganisms and improvement of plant growth.The result in agreement with the findings of (32) who observed that longer plants when potato plants were applied with farm yared manure (13.5tha^{-1}) and NPS (245.1kgha^{-1}).lt is also harmonized with the findings of (33) who recorded maximum plant height of french bean from the application of 120 kg N ha^{-1} while, the minimum value was recorded from the control treatment. Another study that was conducted by (34) reflected that the highest value of mung bean plant height (78.08 cm) was recorded from the treatment which received 20:50 NP kg ha^{-1} with inoculation Rhizobium as compared to the lowest value 68 cm on control treatment. On the other hand, a combined analysis of variance revealed that DBS with N fertilizers had not significant effects on the stem numbers of potato per hill (Table5). This may be due to the parameter more favors genetic makeup, physiological age and tuber seed size rather than a nutrient supplement. This line with finding of (35) shoot number of potato is mostly

determined by the genetic makeup, the physiological age, and the size of potato seed tubers rather than mineral nutrients added in the form of fertilize

3.2.2 Plant height and spike length of wheat

Over year residual analysis of dry-bio slurry on wheat indicated that it was significantly affects both plant height and spike length at ($P < 0.05$) in (Table 5) .The highest values of plant height and spike (93.2 and 9.3 cm) were achieved By application of 100% DBS as compared to untreated plot (Table 5). This might be from the positive effect of DBS for delivering balanced micro and macronutrients as well as enhanced availability of plant nutrients via improving of soil properties. The result agreed with the findings of (36) who observed that the longer plants in plots which are received75%cow dung with 25 %vermicompost than non-treated plots .lt is also harmonized with (37) who recorded maximum plant height(101.5cm) of Boro rice from the application of 5tha-1 trichocompost while the minimum value(78.6) was recorded from control treatment .The result related to spike length also much with (38) who said that an application of 15tha^{-1} bio-gas slurry gives the highest spike length of wheat than checked treatment or control

Table 5. Main and residual effects of dry bio-slurry with equivalence N on growth parameters of potato and wheat

**Treatments**	**Main effect of DBS on Potato**		**Residual effect DBS on Wheat**	
	**PH (cm)**	**NSPH**	**PH(cm)**	**SL(cm)**
Control (0,0)	29.4^{de}	3.3	68.2d	6.7d
RN (138Nkgha^{-1})	52.1^{a}	4.8	89.1c	8.5c
50% DBS (5.3tha^{-1})	28.2^{e}	4.4	92.3ab	8.7bc
100% DBS (10.6tha^{-1})	30.4^{de}	4.4	93.2a	9.3a
75%DBS(7.95tha^{-1})	36.6^{cd}	4.5	93.0ab	9.0abc
75%DBS+25%N (7.95tha^{-1}+34.5Nkgha^{-1})	41.9^{bc}	4.6	92.1abc	9.0abc
50%DBS+50%N (5.3tha^{-1}+69Nkgha^{-1})	42.7^{bc}	3.7	90.2bc	8.9abc

25%DBS+75%N (2.65tha^{-1}+103.5Nkgha^{-1})	48.8^{ab}	4.0	90.2bc	8.7bc
100%DBS+25%N (10.6tha^{-1}+34.5Nkgha^{-1})	44.0^{bc}	4.3	92.1ab	9.0abc
100%DBS+50%N (10.6tha^{-1}+69Nkgha^{-1})	47.1^{ab}	4.2	90.6abc	9.1ab
**LSD**	**7.9**	**NS**	3.0	0.5
**CV%**	**20.9**	**30.1**	3.6	6.6

Means followed by the same letter (s) within the column are not significantly different at ($P \leq 0.05$). DBS= dry bio slurry, N= nitrogen, PH= plant height, NSPH = number of stems per hill, SL=spike length.

3.2.3 Number of tubers per plant and total tuber yield of potato

The combined analysis result in both years across sites indicates that yield and yield a component of potato was significantly differ at (P<0.05) due to the effects of DBS with equivalence N (Table 6). The application of 75% N with 25% DBS gives the highest fresh total tuber yield (27.6tha^{-1}) while the lowest fresh tuber yield (8.6tha^{-1}) was observed at control. This might be due to the release of N from dry bio-slurry (DBS) and Urea to soil solution that makes for plant better growth and development. Moreover, it could be due to the addition of both macro and micro nutrients from the dry bio-slurry (DBS) by improving of soil pH, organic carbon,total nitrogen phosphorus and cation exchange capacity. This study is in line with the findings of (11) who revealed that, the supplying of recommended inorganic fertilizer (100kg DAP, 50kg Urea and 50kg Murate potash per hectare) with 8tha^{-1}bio-slurry gives maximum (266.7 t ha^{-1}) yield of cabbage as compare to the lowest (160 t ha^{-1}) from the control treatment and it gives about 66.7% yield increment due to the combination both bio-slurry and recommended fertilizers over control. On the other hand the study done by (39) revealed that the lowest value of fresh shoot biomass and marketable yield of potato tuber were achieved from control while the highest values were obtained in plots that treated combined application of farm yard manure and recommended nitrogen and phosphorus.

Similarly number of tubers per plant (NTPP) was significantly affected at ($P<0.05$) by the application DBS with equi-valence nitrogen. Maximum value of NTPP was observed through the application of recommended NP as compare to control treatment (Table 6).Even if the maximum value occurs at Recommended NP the other dry bio slurry-nitrogen combination treatments also give better yield advantage than control. This might be due to; the harmonization of organic and inorganic fertilizers for uptake and assimilation of nutrients to potato tubers by increasing the availability of native soil nutrients through higher biological activities. The result coincides with (40) who observed that an application of combined organic and inorganic fertilizers increasing number of tubers per plant in treated plots than control or untreated plots. Another study done by (26) showed that an addition of dry bio-slurry with recommended nitrogen phosphorus increase 40 to 73% number of fruits per plant on tomato.

3.2.4 Grain yield and aboveground biomass of wheat

Yield and aboveground biomass of wheat was significantly differ at ($P<0.05$) (Table 6) by residual effect of dry bio slurry. The application of 100%DBS gives the highest grain yield (3.85tha^{-1}) as compare to control. Similarly, addition of 75%DBS gives the highest aboveground biomass yield of wheat (9.59tha^{-1}) as compare to control (Table 6). Releasing N from dry bio-slurry (DBS) to soil solution may makes for plant better growth and development. Moreover, it could be due to the addition of both macro and micro nutrients from the dry bio-slurry in to soil solution as residual time friend. Beyond its positive effect on soil improvement by increasing the availability of native soil nutrients. The result coincides with (38) who reported the supplement of 10 and15 t ha^{-1} increasing grain yield of wheat significantly by scored maximum yield than control. Similarly study done by (41) revealed that he application of combined use of biogas slurry and chemical fertilizer @ 50% has a good strategy for sustainable crop yield by improving

soil health. Beside to this (42) also showed that an addition of 100% dry bio-slurry increase Stover and stalk yield of maize by 45.5 and 42.2% than control treatments through biological activities

Table6.Response of potato and wheat yield parameters for dry bio slurry and equivalence nitrogen main and residual effect

Treatments	**Main effect of DBS on Potato**		**Residual effect DBS on Wheat**		
	NTPP	TYD t ha^{-1}	GY(t ha^{-1})	BY(tha^{-1})	1000 SW(g)
Control (0,0)	4.2^{e}	8.6^{d}	1.23d	3.11d	30.1
RN (138$Nkgha^{-1}$)	10.9^{a}	26.2^{ab}	3.17c	7.9c	32.4
50% DBS (5.3tha^{-1})	5.0^{de}	12.0^{cd}	3.33bc	8.37bc	32.8
100% DBS (10.6tha^{-1})	6.4^{cd}	13.4^{c}	3.85a	9.52ab	33.6
75%DBS(7.95tha^{-1})	7.3^{bc}	14.9^{c}	3.83a	9.59a	33.9
75%DBS+25%N (7.95tha^{-1}+34.5$Nkgha^{-1}$)	6.5^{bcd}	23.0^{b}	3.47abc	9.08ab	33.2
50%DBS+50%N (5.3tha^{-1}+69$Nkgha^{-1}$)	6.4^{cd}	24.6^{ab}	3.47abc	8.53abc	32.1
25%DBS+75%N (2.65tha^{-1}+103.5$Nkgha^{-1}$)	6.8^{bc}	27.6^{a}	3.28bc	8.43abc	32.6
100%DBS+25%N (10.6tha^{-1}+34.5$Nkgha^{-1}$)	7.0^{bc}	25.2^{ab}	3.70ab	9.56a	33.0
100%DBS+50%N (10.6tha^{-1}+69$Nkgha^{-1}$)	8.1^{b}	26.8^{a}	3.59abc	9.06abc	32.4
**LSD**	1.7	3.7	0.5	1.2	NS
**CV%**	25.6	19.4	15.6	14.8	10.1

Means followed by the same letter (s) within the column are not significantly different at ($P \leq 0.05$).
DBS= dry bio slurry, N= nitrogen, PH= plant height, NTPP = number of tubers per plant, TYD, = total tuber yield, GY= grain yield, BY= above ground biomass and SW = seed weight.

3.3 Economic Analysis

Net benefits were calculated by current fertilizer (Urea) cost of 0.27US$ kg^{-1}, cost of DBS kg^{-1} 0.004US$, field price of potato was 0.12US$$kg^{-1}$, cost of labor per man day in the area is 1.4US$ The marginal rate of returns 100% was used to determine the acceptability of treatments. This economic analysis indicated that most of treatments give high net benefit than control (Table 7).

Addition of 50%DBS with 50%RN and 25%DBS with 75%RN gave (84.5US$) and (91.6US$) net benefit with marginal rate of return of 8461.1% and 4486.3% respectively. This indicate that for every 1US$ invested for 50%DBS with 50%RN and 25%DBS with 75%RN in field, farmers can obtain an additional 84.611 and 44.863 US$ respectively (20). All Un-dominated treatment rates could be acceptable for potato producers in the study area except the dominated one's.Therefore, the most economical rate for producers with low cost and higher benefit was 25%DBS with 75%RN.As second option farmers can used 50%DBS with 50%RN for potato production because of it also have promising net benefit with marginal rate of return

Table 7. Partial budget and marginal analysis of potato as affected by the application of dry bio-slurry with nitrogen at Yilemana Densa District.

Treatments (RN +DBS kgha^{-1})	10% Adjusted tuber Yield t ha^{-1}	Total variable Cost US$ha^{-1}	Net Benefits US$ ha^{-1}	MRR%
Control	7.74	0	928.8	-
50% DBS	10.8	35.2	1260.8	943.2
75%DBS	13.41	52.8	1556.4	1679.6
100% DBS	12.06	70.4	1376.8	D
50%DBS+50%N	22.14	84.5	2572.3	8461.1
25%DBS+75%N	24.84	91.6	2889.2	4486.3
100%DBS+25%N	22.68	91.1	2626.5	D
RNP	23.58	98.7	2730.9	D
100%DBS+50%N	24.12	119.7	2774.7	D
75%DBS+25%N	20.7	217.5	2266.5	D

RNP=percent of recommended nitrogen and phosphorus in kg per hectare, DBS=dry bio-slurry in kilogram per hectare, MRR= marginal rate of return; D= is dominated treatments

4. Conclusion and Recommendation

Yield and yield components of potato and wheat were significantly affected by main and residual effect of dry bio slurry with nitrogen supplement. Both Potato and wheat grown on by addition

of dry bio-slurry with nitrogen was improved in yield and yield components as compared to control. Applied dry bio-slurry with N (nitrogen) had a positive impacts on plant height, number of tubers per plant, total tuber yield, grain yield and above ground biomass. Supplement of Dry bio-slurry with N rate in most treatments gives higher net benefit when compared to control. Among treatments used for this experiment undominated once gave better net benefit response with over 100% marginal rate of return than control in the main crop (potato) and residual crop (wheat). Therefore, resource poor producers or small scale farmers can benefited, if they apply these soil improvement rates depending on their convenience. Related to this application 25%DBS with 75% N gives a higher net benefit with 4486.3% marginal return. As a result, small-scale farmers could use it for potato-wheat production system in the area. In a mixed farming system like the Yilemana Densa district livestock production is a component of their livelihood; cattle manure and biodigester plantation is available. Hence, farmers should be encouraged to practice these fertilizers in order to produce vegetables and cereal crops in their backyards and nearest farms. Finally, similar studies will be done in different locations, crops, years and forms of bio-slurry in order to confirm the current findings and to give a concrete recommendation for crop production and soil health amendment in the study area.

Competing of interest

There is no conflict of interest between Authors

Acknowledgment

The author is grateful to Adet Agricultural research center for its administrative and budget support

Financial disclosure

The research supported by budget by Amhara Agricultural Research Institute (ARARI) and the funder hand no role in data collection and designs and none of the authors had not receive salary from the funder

Reference

1. Brankatschk,G, Finkbeiner, M. Modeling crop rotation in agricultural LCAs - challenges and potential solutions. Agric.Syst.2015;138,6676.https://doi.org/10.1016/j.agsy.2015.05.008.

2. Nemecek, T., Hayer, F., Bonnin, E., Carrou´ee, B., Schneider, A., Vivier, C. Designing eco-efficient crop rotations using life cycle assessment of crop combinations. Eur. J. Agron.2015; 65, 40–51. https://doi.org/10.1016/j.eja.2015.01.005.

3. Birch PRJ, Bryan G, Fenton B, Gilroy EM, Hein I and Jones JT. Crops that feed the world 8: potato: are the trends of increased global production sustainable? Food Secur.2012;4(4):477–508. https://doi.org/10.1007/s12571-012-0220-1.

4. CSA (Central Statistical Agency). Agricultural sample survey 2019/20 (2012 E.C) volume I. Report on area and production of major crops meher season crops for private peasant holdings. 2020;CSA, Addis Ababa.

5. CSA (Central Statistical Agency). Agricultural sample survey: Area and production of major crops. 2021;Addis Ababa, Ethiopia.

6. FAOSTAT. Food and Agriculture Organization corporate statistical database of the United Nations. 2021;Retrieved from http://www.fao.org/faostat/en/#data/QC

7. Efrem B, Hirut K, Getachew B. Durum wheat in Ethiopia: an old crop in an ancient land. 2000;Addis Ababa: Institute of Biodiversity Conservation and Research.

8. Chanie, C., Teshome, T., Temesgen, T., & Berihun, B. Characterization of potato production, marketing, and utilization in North Western Amhara Region, Ethiopia. Journal of Horticulture and Forestry.2017;9(3), 17–25. doi:10.5897/JHF

9. Shankarappa TH. Gurumurthy SB, Patil SV and Lokesh MS. (2012). Influence of phosphorus enriched biogas spent slurry (BSS) on growth and yield of sunflower (Helianthus annuus). Journal of Plant Science.2012; 7: 253-258

10. Khan SA, Malav LC, Kumar S, Malav MK and Gupta N Resource utilization of biogas slurry for better yield and nutritional quality of baby corn. Advances in Environmental and Agricultural Science.2015;382-394.

11. Yalemtsehay D and Fisseha I. Comparative study on the effect of applying biogas slurry and inorganic fertilizer on soil properties, growth and yield of White Cabbage (Brassica oleracea var. capitata f. alba). Journal of Biology, Agriculture and Healthcare. 2016;6(19).

12. Karki KB. Estimation of plant nutrient loss from biogas slurry. Biogas Support Programme.1997;

13. Errebhi, M., Rosen, C.J., Gupta, S.C. and Birong, D.E. 1998. Potato Yield Response and Nitrate Leaching as Influenced by Nitrogen Management. Agron. J. 1998;90:10–15.

14. Alva, A. 2004. Potato nitrogen management. J. Veg. Crop Prod.2004; 10: 97-130.

15. White, P.J., Wheatley, R.E., Hammond, J.P. and Zhang, K. Minerals, soils and roots. In: Vreugdenhil D (ed) Potato biology and biotechnology, advances and perspectives. Elsevier, Amsterdam.2007;pp: 739-752.

16. Zelalem, A., Tekalign, T. and Nigussie, D. Response of potato (Solanum tuberosum L.) to different rates of nitrogen and phosphorus fertilization on vertisols at DebreBerhan, in the central highlands of Ethiopia. African journal of plant science.2009;3: 16-24.

17. Rhaman M.S., Kibria M.G., Hossain M., and Hoque M.A. Effects of organic manure and bio-slurries with chemical fertilizers on growth and yield of rice (cv. BRRI dhan28). Int. J. Expt. Agric.2016;6 (3): 36-42

18. Eyasu E . Soils of the Ethiopian highlands: Geomorphology and Properties. CASCAPE Project, ALTERA, Wageningen University and Research Centre (Wageningen UR), the Netherlands.2016; p. 385

19. Sahlemedihn S and Taye B. Procedures for soil and plant analysis.2000; Addis Ababa, Ethiopia.

20. CIMMYT. From Agronomic Data to Farmer's Recommendation: an Economic Training Manual. 1988;Revised Edition. Mexico, D.F.

21. Gomez K.A and A.A. Gomez. Statistical procedures for agricultural research, 2nd edition. John Wiley and Sons, New York. International Congress Series.1984; 1291: 42-52.

22. Tekalign T. Soil, plant, water, fertilizer, animal manure and compost analysis. Working Document No. 13. 1991;International Livestock Research Center for Africa, Addis Ababa.

23. Olsen S.R, Cole C.V, Watanabe F.S and Dean L.A. Estimation of available phosphorus in soils by extraction with sodium carbonate. USDA Circular.1954;939: 1-19.

24. Landon J.R. Booker tropical soil manual: a handbook for soil survey and agricultural land evaluation in the tropics and subtropics. New York;1991.

25. Workineh E,Yihenew G, Eyasu E and , Matebe D.. Integrated fertilizer application improves soil properties and maize (Zea mays L.) yield on Nitisols in Northwestern Ethiopia. Journal of Helyion.2021;7-e06074

26. Geremew B , Girma A and Birhanu B. Effects of dry bioslurry and chemical fertilizers on tomato growth performance, fruit yield, and soil properties under irrigated conditions in Southern Ethiopia.African Journal of Research.2019; 14(33), pp. 1685-1692.

27. Tsegaye T, Tewodros A and Hussien M.2018. Combined Application of Bioslurry and Inorganic Fertilizers on Quality Traits of Cabbage and Soil Properties. Asian Journal of Biological Sciences.2018;11 (1): 24-32

28. Zelalem A, Tarekegn & Hussien M.2020. Effect of liquid bio-slurry and nitrogen rates on soil physico-chemical properties and quality of green bean (Phaseolus vulgaris L.) at Hawassa Southern Ethiopia.Journal of plant interaction.2020;15 (1):207–212.

29. Sandeep S & Salwinder S.D. Effect of Sewage Sludge and Rice Straw Compost on Yield, Micronutrient Availability and Soil Quality under Rice–Wheat System. Journal Communications In Soil Science and Plant Analysis.2019; DOI:10.1080/00103624.2019.1648489.

30. Shahzad, K., A. Khan, J.U. Smith, M. Saeed, S.A. Khan and S.M. Khan. Residual effects of different tillage systems, bio slurry and poultry manure on soil properties and subsequent wheat productivity under humid subtropical conditions of Pakistan. International journal Biosci.2015; 6(11): 99-108.

31. Tamado T, Mitiku W. 2017. Effect of combined application of organic and mineral nitrogen and phosphorus fertilizer on soil physico-chemical properties and grain yield of food Barley (Hordeum vulgare L.) in Kaffa Zone, South-western Ethiopia. MEJS.2017; 9(2):242–261.

32. Melkamu A, Minwyelet J, Tadele Y & Masho A.2020. Integrated application of compound NPS fertilizer and farmyard manure for economical production

33. Moniruzzaman M, Halim G.M.A and Firoz Z A. Performances of the French bean as influenced by plant density and nitrogen application. Bangladesh. JAR.2009;34(1): 105-111.

34. Muhammad U.C, Muhammad U.H, Imran K. Effect of different nitrogen and phosphorus fertilizer levels in combination with nitrogen and phosphorus solubilizing inoculants on the growth and yield of mung bean. PJLSS. 207;15(1): 31-36.

35. De La, I., Guillen, I. A., & Del Moral, L. F. G. Yield development in potatoes as influenced by cultivar and the timing and level of nitrogen fertilization. American Potato Journal,1994; 71, 165–173. Doi: 10.1007/ BF02849051

36. Balasubramanian A, P. Stalin, M.Saravanaperumal and S.R.Vinoth kumar. Residual effect of Integrated Nutrient Management practices on growth and yield of rice fallow blackgram (Vigna mugno L.). Journal of Emerging Technologies and Innovative Research,2016; 3(5): 2349-5162.

37. Bilkis S, M.R. Islam, M. Jahiruddin, M.M. Rahaman. Integrated Use Of Manure And Fertilizers Increases Rice Yield, Nutrient Uptake And Soil Fertility In The Boro-Fallow-T.Aman Rice Cropping Pattern. SAARC J. Agri,2017; 15(2): 147-161.

38. Pandey B.P, N. Khatri, M. Yadav, K. R. Pant, R. P. Poudel, and A. H. Khan. Effect of Digestate /Biogas Slurry In Wheat Under Rice – Wheat Cropping System. Journal of Agriculture and Forestry University.2020;4: 67-75.

39. Tsegaye G,Birhanu B, S B & Berga L.2020. Integrated Application of Organic and Blended Mineral Fertilizers Improves Potato Productivity and Income for Smallholder Farmers in Acidic Soils. Journal of fertilizer and pesticides.2020;8(3),2471-2728.

40. Haque M.A, M. Jahiruddin, M. S. Islam, M. M. Rahman, M. A. Saleque. Effect of Bio slurry on the Yield of Wheat and Rice in the Wheat– Rice Cropping System. Journal of Agric Res.2018. 7(4):432–442.

41.Shahid, M., A. K. Shukla, P. Bhattacharyya, R. Tripathi, S. Mohanty, A. Kumar, B. Lal, P. Gautam, R. Raja, B. B. Panda., et al. Micronutrients (Fe, Mn, Zn and Cu) balance under long-term application of fertilizer and manure in a tropical rice–rice system. Journal of Soils and Sediments.2016; 16 (3):737–47. doi: 10.1007/s11368-015- 1272-6.

42. Mercy K, Esther M, James N, Kevin K, Stephen Mailu,Peterson Njeru and Rewe Thomas.2022. Integrated effect of liquid bio slurry and inorganic fertilizer on selected soil chemical properties, maize (Zea mays) growth, yield and grain quality. African Journal of Crop Science, 2022;10 (5): 001-016.

43.Liu, E., Yan, C., Mei, X., He, W., Bing, S.H., Ding, L.Long-term effect of chemical fertilizer, straw, and manure on soil chemical and biological properties in northwest China. Geoderma .2010;158, 173–180

44. Efthimiadou A, Bilalis D, Karkanis A and Froud W.B. Combined Organic and Inorganic Fertilization Enhances Soil Quality and Increased Yield, Photosynthesis and Sustainability of Sweet Maize Crop. AJCS.2010; 4(9):722-729.

Figure

Click here to access/download;Figure;Figure1; Study Area.jpg

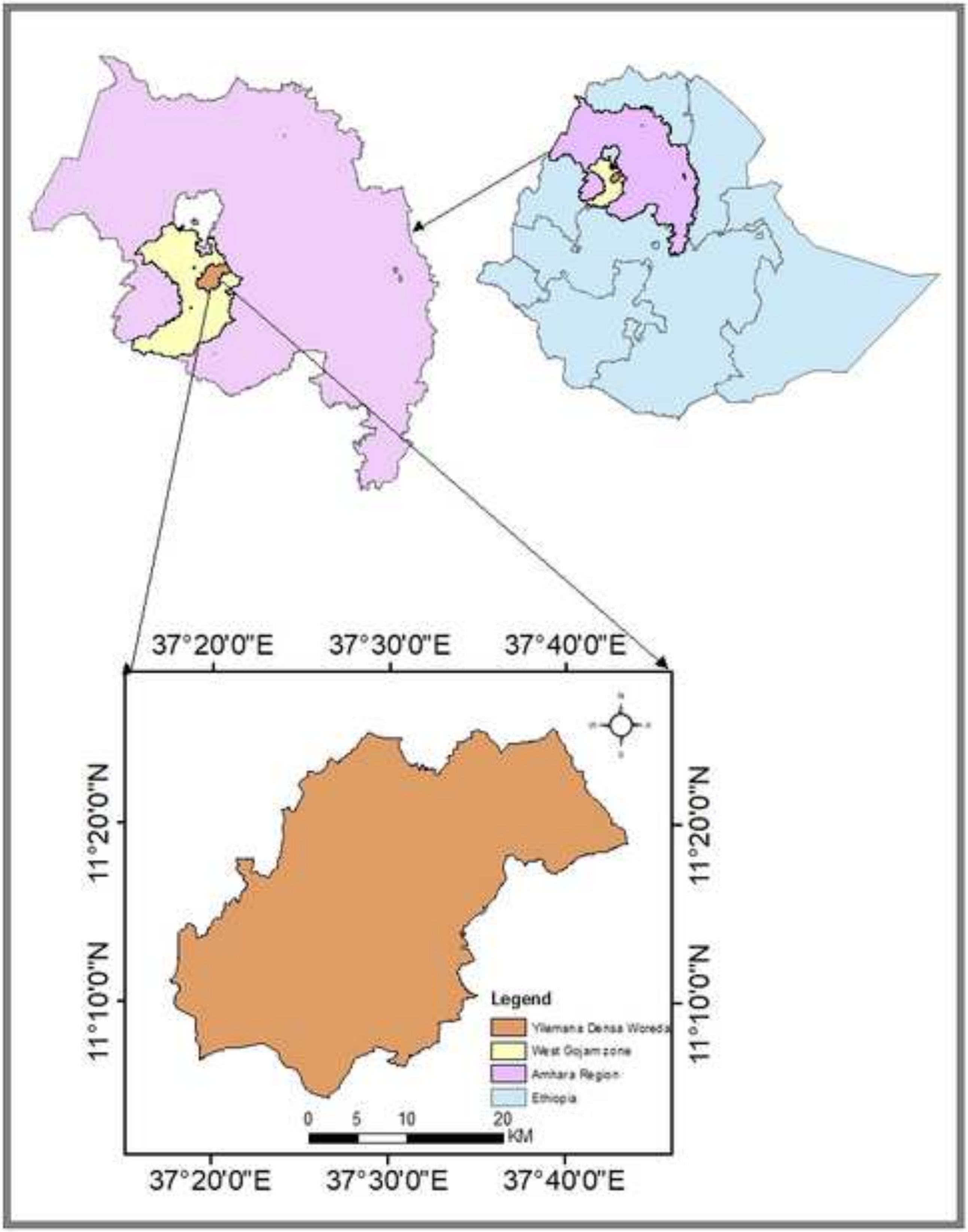

Effects of dry-bio-slurry supplemented with chemical nitrogen fertilizer on growth and yield of potato and wheat under Potato-wheat cropping system

Zelalem Addis*[1],Tadele Amare[1], Bitewlgn Kerebih[1], Antenh Abewa[1], Tesfaye Feysa[2] ,Abrham Awoke[1] and Abere Tenagne[1]

1 Adet Agricultural Research Center P.O.BOX 08, Bahir dar Ethiopia

2 Amhara Agricultural Research Institute

*Corresponding Author zelalemaddis660@gmail.com

ABSTRACT

The experiment was conducted to determine the main and residual effect of dry bio-slurry with equivalence N rates on soil properties and yield components of Potato and wheat in the potato-wheat cropping system at Yelmana Denesa district to improve soil fertility through balanced fertilizer application with the objective of optimizing the rate for yield and soil properties. The experiment comprising ten levels of treatments ~~(~~Control (0.0), RN~~P~~(recommended nitrogen), 50%DBS(dry-bioslurry), 100%DBS(dry-bioslurry), 75%DBS(dry-bioslurry), 75%DBS(dry-bioslurry)+25%RN(recommended nitrogen), 50%DBS(dry-bioslurry)+50%RN(recommended nitrogen), 25%DBS(dry-bioslurry)+75%RN(recommended nitrogen), 100%DBS(dry-bioslurry)+25%RN(recommended nitrogen), and 100%DBS(dry-bioslurry)+50%RN~~)~~ (recommended nitrogen) were laid out in RCBD design with three replications for three years (2019-2021). Data on soil Physico-chemical properties and yield components of potato and wheat were collected and subjected to ANOVA using SAS software. The results indicated that the application of different rates of dry bio-slurry with N fertilizer significantly affected the yield components of both potato and wheat. Soil property except pH is not significantly affected by the main effect of dry bio-slurry. Applying 25% DBS with 75 % RN gives the highest tuber yield

(27.6 tha-1) compared to the control. Similarly, an application of sole 100% and 75% dry bio-slurry scored the highest grain yield (3.85tha^{-1}) and above-ground biomass (9.59tha^{-1}) respectively due to the residual effect on wheat. Similarly, 25%DBS with 75% RN scored the highest net benefit with acceptable marginal return (above 100%) next to 50%DBS with 50% RN. Generally, the application of dry bio-slurry with nitrogen rates can be improved the yield and yield components of potato and wheat in the study area. To attain the highest net benefit, as ~~the main effect of~~ DBS within a short period of time application of 50%DBS with 50%RN can be preferable for yield improvement in the study area and similar agro ecological environments. But ~~to be consistent~~ further research should be done widely and repeatedly in the areas and similar agro –ecologies in permanent plots.

Key Words: dry bio-slurry, nitrogen, soil properties, potato, wheat

1. Introduction

A well planned cropping system can enhance the efficiency of nutrient use, reduce the need for fertilizers and lessen the impact of pests and diseases (1&2). Potato and wheat production under a crop rotation system in the region of Amhara, Ethiopia is commonly practiced by farmers. These raw materials are intended for human and animal consumption. The system comprises a cradle-to-farm analysis for 3-years rotation cycle. It allows mitigating the impact of certain pests and diseases, which significantly affects the yield of the main crop: the potato. In addition to this, it is possible to take full advantage of the residual fertility that the potato leaves in the soil (a crop that is usually abundantly fertilized). This rotation system for potato and wheat crops was chosen by many producers as it is considered the most efficient from an economic, quality and soil health point of view.Potato (*Solanum tuberosum*) is the world's third most important food crop after wheat and rice (3). In Ethiopia, the area under potato cultivation reaches about

73,677.64 ha and its production was 1,044,436.359 tons (4). The productivity of potato in Ethiopia is 13.9 t ha^{-1}(5), which is relatively low compared to other African countries (6). Bread wheat (*Triticum aestivum* L.) is also is one of the major cereal crops grown in the highlands of Ethiopia, and the country is regarded as the largest wheat producer in Sub-Saharan Africa (7).

Out of the total grain crop area, wheat ranked 4th after tef (Eragrostis tef), maize (Zea mays) and sorghum (Sorghum bicolor), while third in total production after maize and tef (5). Despite the long history of wheat cultivation and its importance to Ethiopian agriculture, its average yield is still very low, not exceeding 2.4 t ha^{-1} (5) which is below the world's average of 3.4 t ha^{-1} (6) this phenomenon is common in our region. Low level of potato-wheat productivity is mainly due to soil fertility degradation, improper fertilization, poor pest management practices, use of the low-quality seed, and soil nutrient depletion (8).Enhancing fertility is a precondition for a practical crop production system through organic manure like bio-slurry that can achieve sustainability soil fertility and crop production (9&10). The bio-slurry that obtained after extraction of the energy content of animal manure is an excellent fertilizer, rich in major nutrients (nitrogen, phosphorous and potassium) and organic matter that determine the soil fertility and yield of different crops and vegetables (11). It also improves the physical and biological quality of soil besides providing both macro and micro-nutrients to crops and vegetables. The application of bio-slurry also helps in the reduction of dependence on mineral fertilizers (12). Because both Potato and wheat are highly responsive to N fertilization; it is usually the most limiting essential nutrient for growth and development of those crops (13).In addition, supplying Nitrogen plays an important role in the balance between vegetative and reproductive growth for both crops (14&15). Various studies have shown that N fertilizer applications can increase the dry matter-

protein content of wheat and potato tubers (16). Furthermore, most of the time the available nitrogen in most organic source fertilizers including bio-slurry is high as compared to other nutrients in concentration (11) Due to this dry bio-slurry and nitrogen fertilizers were applied through nitrogen equivalence.Besside ~~Neither~~this Neither organic manure nor Chemical fertilizer alone can meet the nutrient demand of a given crop because ~~it has its own~~their own negative impact in terms of sustainability for crops in the different cropping system via soil-plant interaction (17).~~Forinstance~~For instance continuous addition of ~~chemical fertilizers~~chemical fertilizers can cause soil quality deterioration such as increasing of soil acidity, loss of organic matter and depletion of nutrients that are not supplied in the fertilizer formulation (43). On the other hand organic fertilizers are required in large quantities and there nutrients released slowly and less effect on soil properties and crop yield within short period of time as compared to inorganic fertilizers (44). Because of this, the study was done to determine the main and residual integrated effects of dry-bio slurry and nitrogen fertilizer on potato–wheat cropping system (potato and wheat rotation) for production and soil physic-chemical properties at Yilemana Densa district with flowing specific objectives to determine the optimum rate of dried bio-slurry and nitrogen fertilizer on potato and wheat yield under potato –wheat cropping system and soil Physico-chemical properties improvement in northwestern Amhara region Ethiopia

2. Material and m~~M~~ethods

2.1 Description of Study Area

The experiment was conducted at Yilemana Densa district on Farmer's field across three sites (Y1S1,Y2S1and Y2S2) for three consecutive years (2019-2022) as potato and wheat rotation (potato-wheat cropping system) at Debremewi Keble West Gojam Zone North Western Amhara Region of Ethiopia. Geographically the area lies at 11^0 21' 22" N and 37^0 25' 43" E (Figure 1)

with a mean altitude of 2304 m above sea level. It receives a mean annual rainfall of 1421 mm with mean minimum and maximum temperatures of 12.29 and 27.56°C, respectively Bureau of Agriculture (BOA). The landforms of the area are characterized by undulating to rolling plateaus, scattered moderate hills, dissected side slopes, and river gorges (18). Based on the district Bureau of Agriculture, the major land use comprises cultivated land (57%), forest and bushes (2%), grazing land (33%), and others (8%). Major crops, grown in the study area are Maize, Tef, wheat, barley, potato, and field pea which take the lion's share. Soil types in the area are Nitisols (45%) Vertisols (30%) and Luvisols (25%). This on-farm experiment was conducted on Luvic Nitisols which is the most dominant soil in the study area

2.2 Experimental procedure

The experiment was -contain ten treatments which includes control (without N), recommended N (138N),dry-bio slurry 50% equivalence (5.3tha^{-1}),dry-bio slurry 100% equivalence (10.6 tha^{-1}), dry-bio slurry75%equivalence(7.95 tha^{-1}),dry-bio slurry75% equivalence (7.95tha^{-1})+25% recommended N (34.5N), dry-bio slurry 50% equivalence (5.3tha^{-1})+50% recommended N (69N),dry-bio slurry 25% equivalence (2.65tha^{-1})+75% recommended N(103.5N),dry-bio slurry100% equivalence (10.6tha^{-1})+25% recommended N(34.5N) and dry-bio slurry 100% equivalence (10.6 tha^{-1}) +50% recommended N (69 N) that laid out in Randomized Complete Block Design (RCBD) with three replications. The rates of dry-bio slurry were adjusted based on potato recommended rate 138 kg N equivalency corresponding to its N content. Urea was used as a source of synthetic 138N whereas 69P_2O_5 was applied in the form of TSP to all plots. The experiment was carried out under rain-fed conditions *Gudenie* and TAY varieties were used as test crops for Potato and wheat respectively in each rainy season one after the other. Total area of each plot was 4.5 m x 3 m (13.5 m^2) having 1m space between plots and 1.5m between blocks.

Commented [A1]: Here recommended nitrogen for potato is 138N that was recommended by our research center in 2018

Formatted: Subscript

The potato was spaced 0.3m between plants and each plot consisted of six rows at 0.75m intervals and the data were collected from the middle four rows. Wheat was planted as a rotated crop in 0.2m and harvested from middle rows by avoiding four rows as a border.

2.2.1 Dry bio-slurry

It was collected from farmers biogas plants and stored under well protected shade for better drying and avoid nutrient loss especially nitrogen. After it drying make fine and breake int small pices for nice application and soil reaction. After this during planting time this well prepared dry-bio slurry was incorporated into the soil at planting period time. Representative composite sample dry-bioslurry was taken from the whole collected dried pit of slurry that was collected from different biogas plants across individual farmers for analysis of (pH), organic carbon (OC%), cation exchange capacity (CEC), total nitrogen (TN%) and available phosphorus (P) by following laboratory procedures (19).

Table 1. Physico-chemical analysis of dry-bio slurry before incorporation in year1and 2 and its average

Dry bio-slurry	Concentration
**Year 1**	
Dry matter%	11.2
OC%	27.8
TN%	1.2
Av P (mg kg^{-1})	112.1
pH (H_2O;1:2.5)	7.9
C:N ratio	22.9
CEC (cmol kg^{-1})	59.8
**Year 2**	
Dry matter%	11.8
OC%	16.3
TN%	1.4

Av P (mg kg^{-1})	91.6
pH (H_2O;1:2.5)	7.7
C:N ratio	11.6
CEC (cmol kg^{-1})	67.4
**Average result of Year1andYear 2**	
Dry matter%	11.5
OC%	22.1
TN%	1.3
Av P (mg kg^{-1})	101.9
pH (H_2O;1:2.5)	7.8
C: N ratio	17.3
CEC (cmol kg^{-1})	63.6

OC%=organic carbon percent, TN%=total nitrogen percent, C: N=carbon to nitrogen ratio, CEC = cation exchange capacity, AvP=available phosphorus, and pH= Power of hydrogen concentration.

2.2.2Gudenie potato variety

Formatted: Line spacing: Double

This varieties is used as attest crop for potato and it was planted as main crop or precursor crop of wheat

2.2.3TAY variety of Wheat

It was planted in the next cropping season after potato which was tested for the determination of dry bioslurry residual effect in potato-wheat rotation cropping system . P_2O_5 was applied during the planting period of time as basal whereas inorganic N for potato was applied in three; one-third third at planting, one-third about 30 days after planting, and the remaining one-third at the beginning of the flowering. While for wheat recommended NP was applied in all plots except control which only received P_2O_5 to see the residual effect of dry bio-slurry in the next crop and nitrogen was supplied, in two split.

2.3 Data Collection, Preparation, and Analysis

2.3.1 **sSoil sampling and analysis before planting**

In each every year before planting, representative soil samples were collected from 0-20 cm depth in a random sampling method from 10 spots in the field by using an auger. All samples were mixed together and one composite sample was formed. The composite sample was grounded using a mortar and passed through a 2 mm sieve for analysis of soil texture, CEC, pH, and available P; whereas a 0.5 mm sieve was used for determining the organic carbon (OC) and total N. Bulk density was determined by the core sampling method.

2.3.2 **Soil sampling and analysis after harvest in potato–wheat cropping system**

The major chemical properties of soil such as OC, pH, CEC, total N and available P were analyzed following the compiled laboratory manual of (19). Soil pH was measured in water at the ratio of 1:2.5 using glass electrode pH meter. The soil OC content was determined following the wet digestion method as outlined by Walkley and Black which involves the digestion of OC in the soil samples with potassium dichromate ($K_2Cr_2O_7$) in a sulphuric acid solution. AvP was determined by Olsen extracting method. The total N content in the soil samples was determined following the Kjeldahl method. CEC was determined by extracting the soil samples with ammonium acetate (1NNH_4OAc) followed by repeated washing with ethanol (96%) to remove the excess ammonium ions in the soil solution. Percolating the $NH4^+$ saturated soil with sodium chloride would displace the ammonium ions adsorbed in the soil and the ammonium liberated from the distillation was titrated using 0.1N NaOH.

Crop data collection

~~Growth parameters~~

Plant height was measured for both potato and wheat at the maturity stage by taking five randomly selected plants from ground~~ level~~ to the top apex and averaging for a single reading. Similarly spike length was measured for wheat at the maturity stage by taking five randomly selected plants from staring point of spike grain to the end of spike grain and averaging for a single reading. While number of plants per hill (stem number per hill) was cllected by counting number stems from five hill of potato at 70% maturity stage and averaging for mean reading

~~**Yield and yield components**~~

The number of tubers per plant potato was taken by counting tubers from five randomly taken plants at the maturity stage from the middle six rows of experimental plots ($9m^2$) and averaged for a single estimation of the mean value. While thousand seed weight for wheat was taken from each treatment hrevestable plot ($11.7m^2$) by counting of 1000 seeds at maturity stage after tershing and make them weighting under sensitive balance for weight reading . Total tuber yield of potato was measured at harvesting by taking all tubers from the middle six rows ($9m^2$) plot area and weighted through balancing balance for total weight reading via avoiding of border effects. Grain yield, and above-ground biomass of wheat was taking at maturity stage from the net harvestable plot($11.7m^2$) area and both of the exposed for balancing balance for measuring and reading weight

2.4 Economic Analysis

Economic analysis was performed to make rational choice among the applied variables in the production of potato. The partial budget and marginal rate of return (MRR) were used for evaluating the change in farming methods that affect partially rather than the whole farm practice and also concerned with planning tool to estimate the profit change within a farm (CIMMYT,

1988). This was computed by adjusting yield downward by a 10% and multiplying it with the local field price (6 Ethiopian Birr per kg of potato). Dominance analysis was done by listing of treatments in an increasing order of cost and that has net benefit less than or equal to treatments with the lower costs that vary is dominated (20).

2.5 Statistical Analysis

All data were subjected to analysis of variance through GLM procedure by using SAS software program version 9.4 (SAS Institute, 2002). List significant ~~test~~ difference (LSD) at 0.05 probability level was employed to ~~separate~~ differentiate treatments means ~~where significant differences exis~~t (21).

3. Results and d~~D~~iscussion

3.1 Soil Chemical Properties before planting and After Harvesting ~~of potato~~of potato

The results of soil chemical properties before and after harvest from each experimental site are indicated in Table 2, 3 & 4. Before-planting soil was acidic in reaction with a pH (H_2O 1:2.5) value of 5.1Y1S1(year one site one) and 5.2Y2S1(year two site one), which is within the range of soil pH for potato production (22) (table 2). The total N, available P, OC, C: N ratio and CEC of the soil Y1S1 and Y2S1 before planting were (0.16&0.16%), (6.9&6.3 mg kg^{-1}), (1.8 &1.5%),(11.1&9.3) and (33.9&30.3cmol (+) kg^{-1}), respectively (Table 2). The total N content of the soil was within the range of medium according to (22) who classified the range of total N < 0.1, 0.1- 0.15, 0.15-0.25 and > 0.25% as very low, low, medium and high, respectively. (23) classified available P content of the range < 5 as very low, 5 -15 as low, 15 -25 as medium and > 25 mg kg^{-1} as high. Hence the available P of the soil before planting lies under the low range. According to (24) the soil OC content ranges of 1-2, 2-4, and 4-6% are rated as low, medium and

high, respectively while cation exchange capacity (CEC) ranges of 5-15, 15-25 and 25-40 cmol kg^{-1} are rated as low, medium and high, respectively.

Based on these ratings the OC (1.4, 1.5 &2.0%) and CEC (33.9, 30.3&30.9 cmol kg^{-1}) before planting of the experimental fields of (Y1S1,Y2S1 and Y2S2) were in the low and high ranges, respectively. Generally, the nutrient contents of the study site Y2S1 is not good in terms of availability of major plant nutrients beside its nice CEC. On the other hand on Y2S2 pH, TN, avP, OC, C: N and CEC pre planting values were 5.5, 0.17%, 6.8ppm, 2.4%, 14.1 and 33.9 respectively. Based on (22) rating the pH value was under moderate while total nitrogen was medium. Similarly, according to (23) available P was under low while OC and CEC were medium and high respectively (24). Y2S2 rather than Y1S1and Y2S1 have good soil fertility status based on its values of soil chemical properties. However, the acidity of soil Y1S1andY2S2 may be cause the sorption of available P. Thus, application of OM like bio-slurry is very essential in order to neutralize soil solution for the availability of nutrients. On the other hands, after harvest all soil chemical properties except soil pH (on Y2S2) were not affected by the application of dry bio-slurry with N equivalence rates (Table3).This might be hydrogen replacement through substitution reaction of basic cations from dry bio slurry in soil solution or rhizosphere

The non-significant effects of the applied treatments on these parameters might be due to the slow release of nutrients from dry bio-slurry that was applied during the experimentation period to soil solution related to short cropping season potato cultivation. However, applied dry bio-slurry and N rates had a significant ($P < 0.05$) effect on pH at site2 in 2020-21soil after harvest as compared to the control (Table 2). Numerically the highest soil pH content was obtained by the application of 100% and 100%+25%N dry bio-slurry as compared to the control (Table 3).

Commented [A2]: Yes here I conduct ANOVA analysis I discuss based on that

The increment of soil pH in treated plots may be related due to the releasing of basic cations from dry bio-slurry into soil solution that makes substitute of acid cations.These results were in agreement with the investigation of (25) who they reported that the combined application of compost with inorganic NPSB in maize increased the soil pH content after harvest as compared to control.

3.2 Residual effect of dry bio-slurry on soil chemical properties after harvesting of wheat

The residual effect of dry bio-slurry on selected soil chemical properties was significantly explained at ($p<0.05$) across sites and years in table 4. Related to this an addition of 100% (10.6tha^{-1}) dry bio-slurry gives the highest TN% (0.207and 0.223) on Y1S1 and YS2 respectively than the control. This might be due to the gradual releasing of Nitrogen dry bios-slurry into soil solution beyond its chelating capacity. The finding lined with (26) who they found that an application of dry bio-slurry with inorganic fertilizers gives the maximum value of total nitrogen than control. Similarly, the study conducted by (27) also indicated that an application of 70cm^3 scored the highest (1.36%) TN as compared to the control which gives 0.07%

On the other hand, OC, C: N and CECwere significantly affected at Y2S1(Table 4) While in Y2S2 only CEC is significant at ($p<0.05$) Table 4.Based on this numerically the highest value of CEC(34.6), C: N (12.82)&OC% (2.20) was observed by application 50%DBS and 100% DBS with 25%N as compared to control on Y2S1.On Y2S2 the maximum value of CEC(cmolkg^{-1}) 36.06 was obtained in plots that receive 100%DBS with 25%N than control. This might be due to the positive effect applied to DBS that enables increasing organic matter and holding capacity of positive cation in the soil exchangeable site. The finding agreed with (28) who revealed that an application of 41.3m^{-3} liquid bio-slurry with 20.5kgha^{-1}N significantly increased soil organic

carbon than untreated plots. The study conducted by (29) also indicated that an application of $10tha^{-1}$ with $50\%Nkgha^{-1}$ significantly increased OC% by scoring the maximum value of 0.67 than the control. Organic amendments significantly enhanced SOC thus had a considerable effect on soil microbes and nutrient availability and uptake this may alter the C: N ratio. However, the addition of external organic matter with a low C: N ratio may induce the mineralization of OM. This makes nitrogen trapped by organic matter; a phenomenon known as the priming effect (30). The application of the 50% DBS and 100% DBS with 25%N gives the highest CEC at Y2S1 AND Y2S2 respectively as compared to the control (Table 3) Such increment in CEC might be due to the application of DBS on soil that makes a negatively charged colloidal site and storehouse of basic cations. The finding agreed with (31) who reported that the use of organic FYM and inorganic fertilizers significantly increased CEC over the control.

Table 2.~~Soil physico-chemical properties before planting~~intial soil properties of the experimental sites

			**2019-20 (Y1S1)**				
**Texture**	BD	pH	TN%	Av P (ppm)	CEC (cmol kg^{-1})	C:N	OC%
**SCL**	1.22	5.1	0.16	6.9	33.9	8.8	1.4
			**2020-21 (Y2S1)**				
**Texture**	BD	pH	TN%	Av P (ppm)	CEC (cmol kg-1)	C:N	OC%
**SCL**	1.33	5.2	0.16	6.3	30.3	9.3	1.5
			**2020-21 (Y2S2)**				
**Texture**	BD	pH	TN%	Av P (ppm)	CEC (cmol kg-1)	C:N	OC%
**SCL**	1.26	5.5	0.17	6.8	33.9	11.8	2.0

NB: SCL= sandy clay loam; BD= bulk density

Table 3.main effect of dry bio slurry with nitrogen on soil chemical properties after harvest of potato

			**2019-20 (Y1S1)**			
**Treatment**	pH	TN%	Av P (ppm)	CEC (cmol kg^{-1})	C:N	OC%
Control (0,0)	5.13	0.12	9.3	29.1	11.6	1.4
RN (138Nkgha^{-1})	5.11	0.12	7.8	29.9	11.61	1.3
50% DBS (5.3tha^{-1})	5.19	0.13	8.8	29.6	11.59	1.4
100% DBS (10.6tha^{-1})	5.18	0.13	9.6	29.7	11.58	1.5
75%DBS(7.95tha^{-1})	5.19	0.13	10.2	29.1	11.62	1.5
75%DBS+25%N (7.95tha^{-1}+34.5Nkgha^{-1})	5.22	0.16	10.1	28.3	11.57	1.9
50%DBS+50%N (5.3tha^{-1}+69Nkgha^{-1})	5.10	0.15	9.1	28.7	11.59	1.7

	pH	TN%	Av P (ppm)	CEC (cmol kg^{-1})	C:N	OC%
25%DBS+75%N (2.65tha^{-1}+103.5Nkgha^{-1})	5.18	0.15	7.5	29.2	11.61	1.7
100%DBS+25%N (10.6tha^{-1}+34.5Nkgha^{-1})	5.12	0.13	12.3	28.3	11.62	1.5
100%DBS+50%N (10.6tha^{-1}+69Nkgha^{-1})	5.15	0.13	11.2	27.6	11.60	1.5
**LSD**	**NS**	**NS**	**NS**	**NS**	**NS**	**NS**
**CV%**	**1.2**	**14.3**	**22.43**	**3.3**	**0.21**	**14.19**
			**2020-21 (Y2S1)**			
**Treatment**	pH	TN%	Av P (ppm)	CEC (cmol kg^{-1})	C:N	OC%
Control (0,0)	5.2	0.15	4.4	27.3	11.59	1.7
RN (138Nkgha^{-1})	5.3	0.16	4.3	27.7	11.59	1.8
50% DBS (5.3tha^{-1})	5.3	0.14	6.5	30.8	11.61	1.7
100% DBS (10.6tha^{-1})	5.4	0.15	6.2	27.4	11.60	1.8
75%DBS(7.95tha^{-1})	5.4	0.15	5.8	28.6	11.58	1.7
75%DBS+25%N (7.95tha^{-1}+34.5Nkgha^{-1})	5.3	0.14	5.4	24.4	11.63	1.7
50%DBS+50%N (5.3tha^{-1}+69Nkgha^{-1})	5.3	0.14	4.5	26.7	11.61	1.6
25%DBS+75%N (2.65tha^{-1}+103.5Nkgha^{-1})	5.2	0.14	4.6	27.5	11.59	1.7
100%DBS+25%N (10.6tha^{-1}+34.5Nkgha^{-1})	5.4	0.17	4.4	28.4	11.59	1.9
100%DBS+50%N (10.6tha^{-1}+69Nkgha^{-1})	5.4	0.16	4.4	28.5	11.61	1.9
**LSD**	**NS**	**NS**	**NS**	**NS**	**NS**	**NS**
**CV%**	**2.12**	**13.5**	**21.6**	**9.0**	**0.17**	**13.5**
			**2020-21 (Y2S2)**			
**Treatment**	pH	TN%	Av P (ppm)	CEC (cmol kg^{-1})	C:N	OC%
Control (0,0)	5.3^{cd}	0.17	12.2	30.6	12.10	2.1

RN (138Nkgha^{-1})	5.4b^{cd}	0.17	7.1	31.9	11.60	1.9
50% DBS (5.3tha^{-1})	5.5^{abc}	0.18	11.5	30.4	11.60	2.0
100% DBS (10.6tha^{-1})	5.6^{ab}	0.18	9.5	32.1	12.62	2.3
75%DBS(7.95tha^{-1})	5.6^{ab}	0.17	13.2	32.1	12.00	2.0
75%DBS+25%N (7.95tha^{-1}+34.5Nkgha^{-1})	5.5^{abc}	0.18	13.3	33.5	11.60	2.1
50%DBS+50%N (5.3tha^{-1}+69Nkgha^{-1})	5.5^{abcd}	0.17	13.1	32.6	12.01	2.1
25%DBS+75%N (2.65tha^{-1}+103.5Nkgha^{-1})	5.3^{d}	0.16	9.5	30.9	11.60	1.9
100%DBS+25%N (10.6tha^{-1}+34.5Nkgha^{-1})	5.6^{a}	0.18	11.6	31.8	11.80	2.1
100%DBS+50%N (10.6tha^{-1}+69Nkgha^{-1})	5.4^{bcd}	0.17	15.3	33.4	11.60	2.1
**LSD**	**2.3**	**NS**	**NS**	**NS**	**NS**	**NS**
**CV%**	**0.22**	**9.9**	**29.1**	**6.1**	**5.9**	**13.8**

Means followed by the same letter (s) within the column are not significantly different at ($P \leq 0.05$). control = is no nitrogen and dry bioslurry,RNP=percent of recommended nitrogen and phosphorus, DBS =dry bio-slurry, pH= power of hydrogen concentration, TN% = total nitrogen percent, AvP = available phosphorus, OC% = organic carbon percent, C: N ratio = carbon to nitrogen ratio, CEC = cation exchange capacity, OM% = organic matter percent, Y1S1= year one site one, Y2S1= year two site one and Y2S2 = year two site two.

Table 4. The residual effect of dry bio-slurry on soil chemical properties after wheat harvesting in the potato-wheat cropping system

**2020-21 (Y1S1)**						
Treatment	pH	TN%	Av P (ppm)	CEC (cmol kg^{-1})	C:N	OC%
Control (0,0)	5.40	0.174bcd	8.97	21.39	9.68	1.69
RN (138Nkgha^{-1})	5.40	0.196a	10.12	21.69	10.01	1.95
50% DBS (5.3tha^{-1})	5.40	0.197a	9.25	26.13	9.64	1.88

100% DBS (10.6tha^{-1})	5.38	0.168d	11.04	23.51	11.81	1.98
75%DBS(7.95tha^{-1})	5.40	0.192ab	9.34	25.60	9.42	1.82
75%DBS+25%N (7.95tha^{-1}+34.5Nkgha^{-1})	5.42	0.172cd	9.07	24.51	11.98	2.06
50%DBS+50%N (5.3tha^{-1}+69Nkgha^{-1})	5.36	0.188abc	10.06	22.09	9.61	1.80
25%DBS+75%N (2.65tha^{-1}+103.5Nkgha^{-1})	5.35	0.202a	10.08	25.11	10.14	2.04
100%DBS+25%N (10.6tha^{-1}+34.5Nkgha^{-1})	5.31	0.194ab	10.46	21.81	11.01	2.13
100%DBS+50%N (10.6tha^{-1}+69Nkgha^{-1})	5.37	0.205a	10/76	23.78	9.35	1.89
**LSD**	NS	0,020	NS	NS	NS	NS
**CV%**	1.3	6.2	12.7	11.9	14.8	13.3
			**2021-22 (Y2S1)**			
Treatment	pH	TN%	Av P (ppm)	CEC (cmol kg^{-1})	C:N	OC%
Control (0,0)	5.61	0.178	10.15	27.70d	7.61d	1.36b
RN (138Nkgha^{-1})	5.53	0.179	10.22	30.52bc	10.31abc	1.85a
50% DBS (5.3tha^{-1})	5.69	0.180	11.56	34.67a	12.25abc	2.20a
100% DBS (10.6tha^{-1})	5.67	0.160	9.85	31.77bc	12.42ab	1.98a
75%DBS(7.95tha^{-1})	5.64	0.194	9.85	31.09bc	9.74cd	1.88a
75%DBS+25%N (7.95tha^{-1}+34.5Nkgha^{-1})	5.70	0.178	11.76	29.56cd	10.79abc	1.90a
50%DBS+50%N (5.3tha^{-1}+69Nkgha^{-1})	5.62	0.167	10.69	30.63bc	11.38abc	1.90a
25%DBS+75%N (2.65tha^{-1}+103.5Nkgha^{-1})	5.54	0.186	9.59	30.37bc	10.00bcd	1.86a
100%DBS+25%N (10.6tha^{-1}+34.5Nkgha^{-1})	5.58	0.172	9.88	31.99bc	12.82a	2.20a
100%DBS+50%N (10.6tha^{-1}+69Nkgha^{-1})	5.44	0.185	10.19	32.91ab	11.78abc	2.17a

**LSD**	NS	NS	NS	2.60	2.61	0.41
**CV%**	2.0	6.7	14.4	4.8	14.1	12.6
			**2021-22 (Y2S2)**			
Treatment	pH	TN%	Av P (ppm)	CEC (cmol kg^{-1})	C:N	OC%
Control (0,0)	5.51	0.176de	10.03	29.43c	12.03	2.12
RN (138Nkgha^{-1})	5.65	0.190bcd	8.76	30.69bc	12.19	2.29
50% DBS (5.3tha^{-1})	5.62	0.186bcde	13.49	35.92 a	12.78	2.38
100% DBS (10.6tha^{-1})	5.62	0.209ab	14.08	33.68ab	11.63	2.43
75%DBS(7.95tha^{-1})	5.76	0.178cde	11.74	34.47a	13.41	2.39
75%DBS+25%N (7.95tha^{-1}+34.5Nkgha^{-1})	5.68	0.207ab	12.93	34.81a	12.29	2.54
50%DBS+50%N (5.3tha^{-1}+69Nkgha^{-1})	5.57	0.202abc	13.08	35.88a	11.47	2.30
25%DBS+75%N (2.65tha^{-1}+103.5Nkgha^{-1})	5.59	0.164e	11.07	33.27ab	13.52	2.19
100%DBS+25%N (10.6tha^{-1}+34.5Nkgha^{-1})	5.66	0.223a	11.51	36.06a	10.09	2.24
100%DBS+50%N (10.6tha^{-1}+69Nkgha^{-1})	5.66	0.207ab	11.21	35.95a	11.75	2.42
**LSD**	NS	0.025	NS	3.78	NS	NS
**CV%**	2.3	7.6	17.8	6.5	10.5	7.3

Means followed by the same letter (s) within the column are not significantly different at ($P \leq 0.05$). RNP=percent of recommended nitrogen and phosphorus, DBS =dry bio-slurry, pH= power of hydrogen concentration, TN% = total nitrogen percent, AvP = available phosphorus, OC% = organic carbon percent, C: N ratio = carbon to nitrogen ratio, CEC = cation exchange capacity, OM% = organic matter percent, Y1S1= year one site one, Y2S1= year two site one and Y2S2 = year two site two.

3.2 ~~Main and residual~~ Eeffects of dry bio slurry on potato and wheat yield and yield ~~components~~ attributers in the potato-wheat cropping system

Formatted: Line spacing: Double

3.2.1 Plant height and number of Stem per hill of potato

Integration of dry bio slurry with N rates was significantly ($P < 0.05$) affects plant height (Table 5). Numerically,the highest values of plant height (52.1 and 48.8cm) was achieved with addition of RNP and 25%DBS with 75%N recpectively as compare control and 50%DBS that gives minimum values (Table 5).Increasing of plant height in response to application of DBS with N fertilizer may be due to the improvement of physico- chemical properties of the soil that resulted increased water absorption and nutrient utilization of the plant.Moreover, application of DBS may deliver balanced micro and macronutrients as well as enhanced availability of plant nutrients,which would help to enhance the metabolic activity of microorganisms and improvement of plant growth.The result in agreement with the findings of (32) who observed that longer plants when potato plants were applied with farm yared manure (13.5tha^{-1}) and NPS (245.1kgha^{-1}).It is also harmonized with the findings of (33) who recorded maximum plant height of french bean from the application of 120 kg N ha^{-1} while, the minimum value was recorded from the control treatment. Another study that was conducted by (34) reflected that the highest value of mung bean plant height (78.08 cm) was recorded from the treatment which received 20:50 NP kg ha^{-1} with inoculation Rhizobium as compared to the lowest value 68 cm on control treatment. On the other hand, a combined analysis of variance revealed that DBS with N fertilizers had not significant effects on the stem numbers of potato per hill (Table5). This may be due to the parameter more favors genetic makeup, physiological age and tuber seed size rather than a nutrient supplement. This line with finding of (35) shoot number of potato is mostly

determined by the genetic makeup, the physiological age, and the size of potato seed tubers rather than mineral nutrients added in the form of fertilize

3.2.2 Plant height and spike length of wheat

Over year residual analysis of dry-bio slurry on wheat indicated that it was significantly affects both plant height and spike length at ($P < 0.05$) in (Table 5) .The highest values of plant height and spike (93.2 and 9.3 cm) were achieved By application of 100% DBS as compared to untreated plot (Table 5). This might be from the positive effect of DBS for delivering balanced micro and macronutrients as well as enhanced availability of plant nutrients via improving of soil properties. The result agreed with the findings of (36) who observed that the longer plants in plots which are received75%cow dung with 25 %vermicompost than non-treated plots .It is also harmonized with (37) who recorded maximum plant height(101.5cm) of Boro rice from the application of 5tha-1 trichocompost while the minimum value(78.6) was recorded from control treatment .The result related to spike length also much with (38) who said that an application of 15tha^{-1} bio-gas slurry gives the highest spike length of wheat than checked treatment or control

Table 5. Main and residual effects of dry bio-slurry with equivalence N on growth parameters of potato and wheat

**Treatments**	**Main effect of DBS on Potato**		**Residual effect DBS on Wheat**	
	**PH (cm)**	**NSPH**	**PH(cm)**	**SL(cm)**
Control (0,0)	29.4^{de}	3.3	68.2d	6.7d
RN (138Nkgha^{-1})	52.1^{a}	4.8	89.1c	8.5c
50% DBS (5.3tha^{-1})	28.2^{e}	4.4	92.3ab	8.7bc
100% DBS (10.6tha^{-1})	30.4^{de}	4.4	93.2a	9.3a
75%DBS(7.95tha^{-1})	36.6^{cd}	4.5	93.0ab	9.0abc
75%DBS+25%N (7.95tha^{-1}+34.5Nkgha^{-1})	41.9^{bc}	4.6	92.1abc	9.0abc
50%DBS+50%N (5.3tha^{-1}+69Nkgha^{-1})	42.7^{bc}	3.7	90.2bc	8.9abc

25%DBS+75%N (2.65tha^{-1}+103.5Nkgha^{-1})	48.8^{ab}	4.0	90.2bc	8.7bc
100%DBS+25%N (10.6tha^{-1}+34.5Nkgha^{-1})	44.0^{bc}	4.3	92.1ab	9.0abc
100%DBS+50%N (10.6tha^{-1}+69Nkgha^{-1})	47.1^{ab}	4.2	90.6abc	9.1ab
**LSD**	**7.9**	**NS**	3.0	0.5
**CV%**	**20.9**	**30.1**	3.6	6.6

Means followed by the same letter (s) within the column are not significantly different at ($P \leq 0.05$). DBS= dry bio slurry, N= nitrogen, PH= plant height, NSPH = number of stems per hill, SL=spike length.

3.2.3 Number of tubers per plant and total tuber yield of potato

Formatted: Line spacing: Double

The combined analysis result in both years across sites indicates that yield and yield a component of potato was significantly differ at (P<0.05) due to the effects of DBS with equivalence N (Table 6). The application of 75% N with 25% DBS gives the highest fresh total tuber yield (27.6tha^{-1}) while the lowest fresh tuber yield (8.6tha^{-1}) was observed at control. This might be due to the release of N from dry bio-slurry (DBS) and Urea to soil solution that makes for plant better growth and development. Moreover, it could be due to the addition of both macro and micro nutrients from the dry bio-slurry (DBS) by improving of soil pH, organic carbon,total nitrogen phosphorus and cation exchange capacity. This study is in line with the findings of (11) who revealed that, the supplying of recommended inorganic fertilizer (100kg DAP, 50kg Urea and 50kg Murate potash per hectare) with 8tha^{-1}bio-slurry gives maximum (266.7 t ha^{-1}) yield of cabbage as compare to the lowest (160 t ha^{-1}) from the control treatment and it gives about 66.7% yield increment due to the combination both bio-slurry and recommended fertilizers over control. On the other hand the study done by (39) revealed that the lowest value of fresh shoot biomass and marketable yield of potato tuber were achieved from control while the highest values were obtained in plots that treated combined application of farm yard manure and recommended nitrogen and phosphorus.

Similarly number of tubers per plant (NTPP) was significantly affected at ($P<0.05$) by the application DBS with equi-valence nitrogen. Maximum value of NTPP was observed through the application of recommended NP as compare to control treatment (Table 6).Even if the maximum value occurs at Recommended NP the other dry bio slurry-nitrogen combination treatments also give better yield advantage than control. This might be due to; the harmonization of organic and inorganic fertilizers for uptake and assimilation of nutrients to potato tubers by increasing the availability of native soil nutrients through higher biological activities. The result coincides with (40) who observed that an application of combined organic and inorganic fertilizers increasing number of tubers per plant in treated plots than control or untreated plots. Another study done by (26) showed that an addition of dry bio-slurry with recommended nitrogen phosphorus increase 40 to 73% number of fruits per plant on tomato.

3.2.4 Grain yield and above-ground biomass of wheat

Yield and above-ground biomass of wheat was significantly differ at ($P<0.05$) (Table 6) by residual effect of dry bio slurry. The application of 100%DBS gives the highest grain yield (3.85tha^{-1}) as compare to control. Similarly, addition of 75%DBS gives the highest above-ground biomass yield of wheat (9.59tha^{-1}) as compare to control (Table 6). Releasing N from dry bio-slurry (DBS) to soil solution may makes for plant better growth and development. Moreover, it could be due to the addition of both macro and micro nutrients from the dry bio-slurry in to soil solution as residual time friend. Beyond its positive effect on soil improvement by increasing the availability of native soil nutrients. The result coincides with (38) who reported the supplement of 10 and15 t ha^{-1} increasing grain yield of wheat significantly by scored maximum yield than control. Similarly study done by (41) revealed that he application of combined use of biogas slurry and chemical fertilizer @ 50% has a good strategy for sustainable crop yield by improving

Commented [A3]: Her control is treatment zero (0,0)

soil health. Beside to this (42) also showed that an addition of 100% dry bio-slurry increase Stover and stalk yield of maize by 45.5 and 42.2% than control treatments through biological activities

Table6.Response of potato and wheat yield parameters for dry bio slurry and equivalence nitrogen main and residual effect

Treatments	**Main effect of DBS on Potato**		**Residual effect DBS on Wheat**		
	NTPP	TYD t ha^{-1}	GY(t ha^{-1})	BY(tha^{-1})	1000 SW(g)
Control (0,0)	4.2^{e}	8.6^{d}	1.23d	3.11d	30.1
RN (138Nkgha^{-1})	10.9^{a}	26.2^{ab}	3.17c	7.9c	32.4
50% DBS (5.3tha^{-1})	5.0^{de}	12.0^{cd}	3.33bc	8.37bc	32.8
100% DBS (10.6tha^{-1})	6.4^{cd}	13.4^{c}	3.85a	9.52ab	33.6
75%DBS(7.95tha^{-1})	7.3^{bc}	14.9^{c}	3.83a	9.59a	33.9
75%DBS+25%N (7.95tha^{-1}+34.5Nkgha^{-1})	6.5^{bcd}	23.0^{b}	3.47abc	9.08ab	33.2
50%DBS+50%N (5.3tha^{-1}+69Nkgha^{-1})	6.4^{cd}	24.6^{ab}	3.47abc	8.53abc	32.1
25%DBS+75%N (2.65tha^{-1}+103.5Nkgha^{-1})	6.8^{bc}	27.6^{a}	3.28bc	8.43abc	32.6
100%DBS+25%N (10.6tha^{-1}+34.5Nkgha^{-1})	7.0^{bc}	25.2^{ab}	3.70ab	9.56a	33.0
100%DBS+50%N (10.6tha^{-1}+69Nkgha^{-1})	8.1^{b}	26.8^{a}	3.59abc	9.06abc	32.4
**LSD**	1.7	3.7	0.5	1.2	NS
**CV%**	25.6	19.4	15.6	14.8	10.1

Means followed by the same letter (s) within the column are not significantly different at ($P \leq 0.05$). DBS= dry bio slurry, N= nitrogen, PH= plant height, NTPP = number of tubers per plant, TYD, = total tuber yield, GY= grain yield, BY= above ground biomass and SW = seed weight.

3.3 ~~Partial Budget~~~~Econmic~~Economic Analysis

Net benefits were calculated by current fertilizer (Urea) cost of ~~13.643 Birr~~0.27US$ kg^{-1}, cost of DBS kg^{-1} ~~0.2Birr~~0.004US$, field price of potato was ~~6 Birr~~ 0.12US$kg^{-1}, cost of labor per man day in the area is 1.4US$~~70~~ Birr. The marginal rate of returns 100% was used to determine the acceptability of treatments. This economic analysis indicated that most of treatments give high

Commented [A4]: This three consecutive years yield potato follow wheat as rotation in permanent plot

Formatted: Line spacing: Double

Commented [A5]: Is the reality during that growing sean not under estimate

net benefit than control (Table 7). Addition of 50%DBS with 50%RN and 25%DBS with 75%RN gave (~~128613.6 birr~~84.5US$) and (~~144460.3 birr~~91.6US$) net benefit with marginal rate of return of 8461.1% and 4486.3% respectively. This indicate that for every ~~1birr~~ 1US$ invested for 50%DBS with 50%RN and 25%DBS with 75%RN in field, farmers can obtain an additional 84.611 and 44.863 ~~Birr~~ US$ respectively (20). All Un-dominated treatment rates could be acceptable for potato producers in the study area except the dominated one's. ~~Even if enough un-dominated alternatives treatments have been available as choice for potato cultivation farmers in the study area. Application of 25%DBS with75%RN and 50%DBS and 50%N scored the most promising result 144460.3 and 128613.6 Birr net benefit respectively as compare to other treatments (Table 7).~~ Therefore, the most economical rate for producers with low cost and higher benefit was 25%DBS with 75%RN.As second option farmers can used 50%DBS with 50%RN for potato production because of it also have promising net benefit with marginal rate of return

Table 7. Partial budget and marginal analysis of potato as affected by the application of dry bio-slurry with nitrogen at Yilemana Densa District.

Treatments (RN +DBS kgha^{-1})	10% Adjusted tuber Yield t ha^{-1}	Total variable Cost ~~Birr~~ US$ha^{-1}	Net Benefits US$~~Birr~~ ha^{-1}	MRR%
Control	7.74	0	~~46440~~928.8	-
50% DBS	10.8	35.2~~1760~~	~~63040~~1260.8	943.2
75%DBS	13.41	~~2640~~52.8	~~77820~~1556.4	1679.6
100% DBS	12.06	~~3520~~70.4	~~68840~~1376.8	D
50%DBS+50%N	22.14	~~4226.45~~84.5	~~128613.6~~2572.3	8461.1
25%DBS+75%N	24.84	~~4579.675~~91.6	~~144460.3~~2889.2	4486.3
100%DBS+25%N	22.68	~~4753.225~~91.1	~~131326.8~~2626.5	D
RNP	23.58	~~4932.9~~98.7	~~136547.1~~2730.9	D
100%DBS+50%N	24.12	~~5986.45~~119.7	~~138733.6~~2774.7	D

Commented [A6]: I have check it in economics analysis procedure data its fully correct

75%DBS+25%N	20.7	~~10873.23~~217.5	~~113326.8~~2266.5	D

RNP=percent of recommended nitrogen and phosphorus in kg per hectare, DBS=dry bio-slurry in kilogram per hectare, MRR= marginal rate of return; D= is dominated treatments

4. Conclusion and Recommendation

Formatted: Line spacing: Double

Yield and yield components of potato and wheat were significantly affected by main and residual effect of dry bio slurry with nitrogen supplement. Both Potato and wheat grown on by addition of dry bio-slurry with nitrogen was improved in yield and yield components as compared to control. Applied dry bio-slurry with N (nitrogen) had a positive impacts on plant height, number of tubers per plant, total tuber yield, grain yield and above ground biomass. Supplement of Dry bio-slurry with N rate in most treatments gives higher net benefit when compared to control. Among treatments used for this experiment undominated once gave better net benefit response with over 100% marginal rate of return than control in the main crop (potato) and residual crop (wheat). Therefore, resource poor producers or small scale farmers can benefited, if they apply these soil improvement rates depending on their convenience. Related to this application ~~50~~25%DBS with ~~50~~75% N gives a higher net benefit with ~~8461.1~~4486.3% marginal return. As a result, small-scale farmers could use it for potato-wheat production system in the area. In a mixed farming system like the Yilemana Densa district livestock production is a component of their livelihood; cattle manure and biodigester plantation is available. Hence, farmers should be encouraged to practice these fertilizers in order to produce vegetables and cereal crops in their backyards and nearest farms. Finally, similar studies will be done in different locations, crops, years and forms of bio-slurry in order to confirm the current findings and to give a concrete recommendation for crop production and soil health amendment in the study area.

Commented [A7]: It is correct I have check it

Competing of interest

There is no conflict of interest between Authors

Acknowledgment

The author is grateful to Adet Agricultural research center for its administrative and budget support

Financial disclosure

The research supported by budget by Amhara Agricultural Research Institute (ARARI) and the funder hand no role in data collection and designs and none of the authors had not receive salary from the funder

Reference

1. Brankatschk,G, Finkbeiner, M. Modeling crop rotation in agricultural LCAs - challenges and potential solutions. Agric.Syst.2015;138,6676.https://doi.org/10.1016/j.agsy.2015.05.008.

2. Nemecek, T., Hayer, F., Bonnin, E., Carrou´ee, B., Schneider, A., Vivier, C. Designing eco-efficient crop rotations using life cycle assessment of crop combinations. Eur. J. Agron.2015; 65, 40–51. https://doi.org/10.1016/j.eja.2015.01.005.

3. Birch PRJ, Bryan G, Fenton B, Gilroy EM, Hein I and Jones JT. Crops that feed the world 8: potato: are the trends of increased global production sustainable? Food Secur.2012;4(4):477–508. https://doi.org/10.1007/s12571-012-0220-1.

4. CSA (Central Statistical Agency). Agricultural sample survey 2019/20 (2012 E.C) volume I. Report on area and production of major crops meher season crops for private peasant holdings. 2020;CSA, Addis Ababa.

5. CSA (Central Statistical Agency). Agricultural sample survey: Area and production of major crops. 2021;Addis Ababa, Ethiopia.

6. FAOSTAT. Food and Agriculture Organization corporate statistical database of the United Nations. 2021;Retrieved from http://www.fao.org/faostat/en/#data/QC

7. Efrem B, Hirut K, Getachew B. Durum wheat in Ethiopia: an old crop in an ancient land. 2000;Addis Ababa: Institute of Biodiversity Conservation and Research.

8. Chanie, C., Teshome, T., Temesgen, T., & Berihun, B. Characterization of potato production, marketing, and utilization in North Western Amhara Region, Ethiopia. Journal of Horticulture and Forestry.2017;9(3), 17–25. doi:10.5897/JHF

9. Shankarappa TH. Gurumurthy SB, Patil SV and Lokesh MS. (2012). Influence of phosphorus enriched biogas spent slurry (BSS) on growth and yield of sunflower (Helianthus annuus). Journal of Plant Science.2012; 7: 253-258

10. Khan SA, Malav LC, Kumar S, Malav MK and Gupta N Resource utilization of biogas slurry for better yield and nutritional quality of baby corn. Advances in Environmental and Agricultural Science.2015;382-394.

11. Yalemtsehay D and Fisseha I. Comparative study on the effect of applying biogas slurry and inorganic fertilizer on soil properties, growth and yield of White Cabbage (Brassica oleracea var. capitata f. alba). Journal of Biology, Agriculture and Healthcare. 2016;6(19).

12. Karki KB. Estimation of plant nutrient loss from biogas slurry. Biogas Support Programme.1997;

13. Errebhi, M., Rosen, C.J., Gupta, S.C. and Birong, D.E. 1998. Potato Yield Response and Nitrate Leaching as Influenced by Nitrogen Management. Agron. J. 1998;90:10–15.

14. Alva, A. 2004. Potato nitrogen management. J. Veg. Crop Prod.2004; 10: 97-130.

15. White, P.J., Wheatley, R.E., Hammond, J.P. and Zhang, K. Minerals, soils and roots. In: Vreugdenhil D (ed) Potato biology and biotechnology, advances and perspectives. Elsevier, Amsterdam.2007;pp: 739-752.

16. Zelalem, A., Tekalign, T. and Nigussie, D. Response of potato (Solanum tuberosum L.) to different rates of nitrogen and phosphorus fertilization on vertisols at DebreBerhan, in the central highlands of Ethiopia. African journal of plant science.2009;3: 16-24.

17. Rhaman M.S., Kibria M.G., Hossain M., and Hoque M.A. Effects of organic manure and bio-slurries with chemical fertilizers on growth and yield of rice (cv. BRRI dhan28). Int. J. Expt. Agric.2016;6 (3): 36-42

18. Eyasu E . Soils of the Ethiopian highlands: Geomorphology and Properties. CASCAPE Project, ALTERA, Wageningen University and Research Centre (Wageningen UR), the Netherlands.2016; p. 385

19. Sahlemedihn S and Taye B. Procedures for soil and plant analysis.2000; Addis Ababa, Ethiopia.

20. CIMMYT. From Agronomic Data to Farmer's Recommendation: an Economic Training Manual. 1988;Revised Edition. Mexico, D.F.

21. Gomez K.A and A.A. Gomez. Statistical procedures for agricultural research, 2nd edition. John Wiley and Sons, New York. International Congress Series.1984; 1291: 42-52.

22. Tekalign T. Soil, plant, water, fertilizer, animal manure and compost analysis. Working Document No. 13. 1991;International Livestock Research Center for Africa, Addis Ababa.

23. Olsen S.R, Cole C.V, Watanabe F.S and Dean L.A. Estimation of available phosphorus in soils by extraction with sodium carbonate. USDA Circular.1954;939: 1-19.

24. Landon J.R. Booker tropical soil manual: a handbook for soil survey and agricultural land evaluation in the tropics and subtropics. New York;1991.

25. Workineh E,Yihenew G, Eyasu E and , Matebe D.. Integrated fertilizer application improves soil properties and maize (Zea mays L.) yield on Nitisols in Northwestern Ethiopia. Journal of Helyion.2021;7-e06074

26. Geremew B , Girma A and Birhanu B. Effects of dry bioslurry and chemical fertilizers on tomato growth performance, fruit yield, and soil properties under irrigated conditions in Southern Ethiopia.African Journal of Research.2019; 14(33), pp. 1685-1692.

27. Tsegaye T, Tewodros A and Hussien M.2018. Combined Application of Bioslurry and Inorganic Fertilizers on Quality Traits of Cabbage and Soil Properties. Asian Journal of Biological Sciences.2018;11 (1): 24-32

28. Zelalem A, Tarekegn & Hussien M.2020. Effect of liquid bio-slurry and nitrogen rates on soil physico-chemical properties and quality of green bean (Phaseolus vulgaris L.) at Hawassa Southern Ethiopia.Journal of plant interaction.2020;15 (1):207–212.

29. Sandeep S & Salwinder S.D. Effect of Sewage Sludge and Rice Straw Compost on Yield, Micronutrient Availability and Soil Quality under Rice–Wheat System. Journal Communications In Soil Science and Plant Analysis.2019; DOI:10.1080/00103624.2019.1648489.

30. Shahzad, K., A. Khan, J.U. Smith, M. Saeed, S.A. Khan and S.M. Khan. Residual effects of different tillage systems, bio slurry and poultry manure on soil properties and subsequent wheat productivity under humid subtropical conditions of Pakistan. International journal Biosci.2015; 6(11): 99-108.

31. Tamado T, Mitiku W. 2017. Effect of combined application of organic and mineral nitrogen and phosphorus fertilizer on soil physico-chemical properties and grain yield of food Barley (Hordeum vulgare L.) in Kaffa Zone, South-western Ethiopia. MEJS.2017; 9(2):242–261.

32. Melkamu A, Minwyelet J, Tadele Y & Masho A.2020. Integrated application of compound NPS fertilizer and farmyard manure for economical production

33. Moniruzzaman M, Halim G.M.A and Firoz Z A. Performances of the French bean as influenced by plant density and nitrogen application. Bangladesh. JAR.2009;34(1): 105-111.

34. Muhammad U.C, Muhammad U.H, Imran K. Effect of different nitrogen and phosphorus fertilizer levels in combination with nitrogen and phosphorus solubilizing inoculants on the growth and yield of mung bean. PJLSS. 207;15(1): 31-36.

35. De La, I., Guillen, I. A., & Del Moral, L. F. G. Yield development in potatoes as influenced by cultivar and the timing and level of nitrogen fertilization. American Potato Journal,1994; 71, 165–173. Doi: 10.1007/ BF02849051

36. Balasubramanian A, P. Stalin, M.Saravanaperumal and S.R.Vinoth kumar. Residual effect of Integrated Nutrient Management practices on growth and yield of rice fallow blackgram (Vigna mugno L.). Journal of Emerging Technologies and Innovative Research,2016; 3(5): 2349-5162.

37. Bilkis S, M.R. Islam, M. Jahiruddin, M.M. Rahaman. Integrated Use Of Manure And Fertilizers Increases Rice Yield, Nutrient Uptake And Soil Fertility In The Boro-Fallow-T.Aman Rice Cropping Pattern. SAARC J. Agri,2017; 15(2): 147-161.

38. Pandey B.P, N. Khatri, M. Yadav, K. R. Pant, R. P. Poudel, and A. H. Khan. Effect of Digestate /Biogas Slurry In Wheat Under Rice – Wheat Cropping System. Journal of Agriculture and Forestry University.2020;4: 67-75.

39. Tsegaye G,Birhanu B, S B & Berga L.2020. Integrated Application of Organic and Blended Mineral Fertilizers Improves Potato Productivity and Income for Smallholder Farmers in Acidic Soils. Journal of fertilizer and pesticides.2020;8(3),2471-2728.

40. Haque M.A, M. Jahiruddin, M. S. Islam, M. M. Rahman, M. A. Saleque. Effect of Bio slurry on the Yield of Wheat and Rice in the Wheat– Rice Cropping System. Journal of Agric Res.2018. 7(4):432–442.

41.Shahid, M., A. K. Shukla, P. Bhattacharyya, R. Tripathi, S. Mohanty, A. Kumar, B. Lal, P. Gautam, R. Raja, B. B. Panda., et al. Micronutrients (Fe, Mn, Zn and Cu) balance under long-term application of fertilizer and manure in a tropical rice–rice system. Journal of Soils and Sediments.2016; 16 (3):737–47. doi: 10.1007/s11368-015- 1272-6.

42. Mercy K, Esther M, James N, Kevin K, Stephen Mailu,Peterson Njeru and Rewe Thomas.2022. Integrated effect of liquid bio slurry and inorganic fertilizer on selected soil chemical properties, maize (Zea mays) growth, yield and grain quality. African Journal of Crop Science, 2022;10 (5): 001-016.

43.Liu, E., Yan, C., Mei, X., He, W., Bing, S.H., Ding, L.Long-term effect of chemical fertilizer, straw, and manure on soil chemical and biological properties in northwest China. Geoderma .2010;158, 173–180

44. Efthimiadou A, Bilalis D, Karkanis A and Froud W.B. Combined Organic and Inorganic Fertilization Enhances Soil Quality and Increased Yield, Photosynthesis and Sustainability of Sweet Maize Crop. AJCS.2010; 4(9):722-729.

Response to Authors

Dear respected editorial we have tried to adjust the manuscript by tracking, coloring based on your comments and without tracking

And here we also put the general response to the author's comment

**Comments**	**Response**
Reviewer 1<br>.title: It is suggested to rephrase as "Effects of dry-bio-slurry supplemented with chemical nitrogen fertilizer on growth and yield of potato and wheat under Potato-wheat cropping system<br>Abstract<br>• It is recommended to include problem statement and objective of the study at the beginning of the abstract.<br>• The conclusion should be changed after correcting the MRR analysis in the results and discussion.<br>Introduction<br>• The authors should justify why intergraded application of organic and inorganic fertilizer is better than the solo application of the fertilizers.<br>• Previous research experiences in the integrated application of fertilizers should be reviewed.<br>• Most of the information/references are very old and the authors should change them with recent references<br>• There are sentences that are difficult to understand and unfinished, which should be revised<br>• A lot of grammatical and typographical errors that should be revised<br>• For more information please refer the commented document	• We have are adjust the title based on reviewer **"Effects of dry-bio-slurry supplemented with chemical nitrogen fertilizer on growth and yield of potato and wheat under Potato-wheat cropping system**<br>• Here we also incorporate statement of the problem at the begging of abstract<br>• we have tried to check MRR by back tracing of the data its correct but our recommendation and conclusion adjusted from 50%RN+ 50DBS to 25%DBS +75%RN<br>• we have justified it in the manuscript based on the comment<br>• in case of reference we have tried to explorer recent works and adjust it based on the comment<br>• we also tried adjust the language errors by the application of Grammerly
Methodology<br>This part of the manuscript should be given due attention and requires rigorous revision. Description of the study area, the production system and data collection methods implemented (growth and yield) should be clearly described so that it is understandable and could be repeated by other person (for more information, please refer the attached document).	We are tried to more clarify based on the comment

Results and discussion<br>The way results are presented is not attractive for the reader. It is suggested to present first the results and then forward why it could be and then compare the results with others.<br>Results should be presented in the past tense form.<br>Although no description of site 1 and site 2 in the methodology, results of site 1 and site 2 (soil sample results) are presented, which are confusing.<br>The economic analysis should be done once again as the MRR of 50%DBS+50%N (8461.1%) is not correct. Accordingly, the conclusion and recommendation part should be revised based on the corrected MRR value.<br>For more information, refer commented document<br>Conclusion and recommendation<br>It should be revised after the revision of the MRR values.	In this we make it in main body of the manuscript through tracking
References<br>The way reference listed should be uniform and meet the guideline of the journal.	Based on journal guide line we tried to adjust it
eviewer #2: I must appreciate authors for nice work in which they have tried to understand supplementation of N with DBS on the performance of Potato-wheat cropping system and soil quality. The study has been conducted by following established procedures and methods and is scientifically sound. The data recorded and presented in the MS are also in order. I have thoroughly gone through the MS and realized that there is still scope to further improve its quality, presentation and content. According some suggestions/comments have been tendered on the MS itself. These comments are easy to understand and self explanatory. In addition to these comments, some specific comments are as under. Authors are advised to revise the MS by duly incorporating all the suggestions/comments.<br><br>1. English language polishing is highly needed to bring the information of the MS in presentable form.<br><br>2. Authors are advised to include more yield attributes of both the crops for better and clear understanding of treatment effects on test crops.	•we also tried adjust the language errors by the application of Grammerly and for the sake of soil we only interested the listed yield parameters rather to other attributers .because of that we only use that parameters for those crops

In addition to the above, it is recommended to include system equivalent yield as study has been attempted in system mode.<br><br>3. Include recent and updated works to support findings. Authors are also advised to cross check the work cited in text and presented in the reference section and vice versa.<br><br>Reviewer #3: The research holds importance in the area where its being conducted. Although the program is well planned but it needs to be re written. The introduction is very weak, does not fully justify the topic. It needs to be revised keeping in view all the important parameters in the title. The results too are poorly written and discussed with most of the incomplete sentences.<br>the abstract also needs to be rewritten..	.Yes we check it and adjust based on yours comment for all reference
Reviewer #4: Starting from the topic need major and careful modifications. The abstract does not contain the mail elements and it is poor. The result section is not properly interpreted. conclusion and recommendation section are poor. Majority of the references are very old.	.We have already adjust it based on reviewer 1 comment
8. Please extract some data from table and present in good-quality figures. figures must be >300 dpi.	.Here We have think it's better put it in table rather tables
