# Supplementary material for: Effects of dry bio-slurry and nitrogen fertilizer on potato and wheat yields under rotation cropping system
Source: PLoS One. 2024 Jul 31;19(7):e0306625. doi: 10.1371/journal.pone.0306625 (PMC11290689; doi:10.1371/journal.pone.0306625)
Supplement: Attachment — Submitted filename: PONE-D-23-10312_R3.pdf [file pone.0306625.s010.pdf]

PLOS ONE

Effects of dry-bio-slurry with nitrogen fertilizer on yield of potato and wheat under short term crop rotation

--Manuscript Draft--

**Manuscript Number:**	PONE-D-23-10312R3
**Article Type:**	Research Article
**Full Title:**	Effects of dry-bio-slurry with nitrogen fertilizer on yield of potato and wheat under short term crop rotation
**Short Title:**	Effects of dry bioslurry with nitrogen fertilizer on yields of potato and wheat under rotation cropping system
**Corresponding Author:**	Zelalem Addis Musse, Msc<br>Amhara Regional Agricultural Research Institute<br>bahirdar, Amhara ETHIOPIA
**Keywords:**	dry bio-slurry, nitrogen, soil properties, potato, wheat
**Abstract:**	Effective cropping system with nutrient balancing is the key solution in farming practices by improving of nutrient use efficiency, reduce need for inorganic fertilizers and impact of pests and diseases. The experiment was conducted to fill the gap of using balanced nutrient in crop rotation system for the improvement of both soil properties and crop yields. That was done by substitution balancing ratio of dry bio-slurry and nitrogen fertilizers for the enhancement of soil properties and potato-wheat yields. The experiment was contain ten levels of treatments Control (0.0), RN (recommended nitrogen), 50% DBS (dry-bio slurry), 100% DBS (dry-bio slurry), 75% DBS (dry-bio slurry), 75% DBS (dry-bio slurry)+25% RN (recommended nitrogen), 50% DBS (dry-bio slurry)+50% RN (recommended nitrogen), 25% DBS (dry-bio slurry)+75% RN (recommended nitrogen), 100% DBS (dry-bio slurry) +25% RN (recommended nitrogen) and 100% DBS (dry-bio slurry)+50% RN (recommended nitrogen) that were laid out in RCBD design with three replications for three years. Data on soil Physico-chemical properties and yield components of potato and wheat were collected and subjected to ANOVA using SAS software 9.4. The results were indicated that; an application of dry bio-slurry with nitrogen fertilizer was significantly affected the yield and yield components of both potato and wheat in their rotation system. Soil properties also, significantly changed by the residual effect of dry bio-slurry. Applying 25% DBS with 75 % RN was given the highest tuber yield of potato (27.6 tha-1) as compared to control. Similarly, an application of 100% and 75% sole dry bio-slurry scored the highest grain yield (3.85tha-1) and above-ground biomass (9.59tha-1) of wheat respectively. The combination of 25% DBS with 75% RN also, recorded the highest net benefit (2889.2 US$) with acceptable marginal return (4486.2%). So that, to attain the highest net benefit, application of 25% DBS with 75% RN could be promoted for yield improvement in the study area and similar agroecology.
**Order of Authors:**	Zelalem Addis Musse, Msc
	Tadele Amare Kassie, PhD
	Bitewelegn kerebeh Kassahun, BSC
	Antenh Abewa Mogesie, PhD
	Tesfaye Feysa Beyene, PhD
	Abrham Awoke Tshale, Bsc
	Abere Tenagne Molla, Bsc
**Response to Reviewers:**	Response for reviewer 1<br>I.We have been adjusting the title based on reviewer 4 suggestion “Effects of dry bio slurry with nitrogen fertilizer on yields of potato and wheat under short term crop rotation system by considering the comment of reviewer 1<br>II.Here we are done the re -articulation of for statement of the problem in the abstract part based on the reviewer comment<br>III.Based on the comment we in corporate the previous findings that deals the

Powered by Editorial Manager® and ProduXion Manager® from Aries Systems Corporation

	integration effect of organic and inorganic fertilizers on growth and soil properties<br>IV.Here we correct both MRR and net plot area-based reviewer comment<br>V.We have been making it similar in both parts of the manuscript for recommendation<br>VI.Here we want to appreciate all of the reviewers including reviewer1 for their effort as manuscript be well edited by language writing. by fully considering and give attention for there frequent comment we have correct and check it by fluent speaker and language skilled persons in addition to our correction effort .so the manuscript well edited and corrected in terms of language<br>Response for reviewer 4<br>I.We have been adjusting the title based on reviewer suggestion “Effects of dry bio slurry with nitrogen fertilizer on yields of potato and wheat under short term crop rotation system<br>II.we incorporate the background of the problem in abstract part based on this comment (reviewer 4).<br>III.we are correct it in both parts by summarize.<br>IV.we make clear and short in most lines and sentences in the manuscript.<br>V.Here we want to appreciate all of the reviewers including reviewer4 for their effort as manuscript be well edited by language writing. by fully considering and give attention for their frequent comment we have correct and check it by fluent speaker and language skilled persons in addition to our correction effort .so the manuscript well edited and corrected in terms of language.<br>VI.We correct it by 2014.<br>VIII.we also correct it based on the comment
**Additional Information:**	
**Question**	**Response**
**Financial Disclosure**<br><br>Enter a financial disclosure statement that describes the sources of funding for the work included in this submission. Review the submission guidelines for detailed requirements. View published research articles from *PLOS ONE* for specific examples.<br><br>This statement is required for submission and **will appear in the published article** if the submission is accepted. Please make sure it is accurate.	The research supported by budget by Amhara Agricultural Research Institute (ARARI) and the funder hand no role in data collection and designs and none of the authors had not receive salary from the funder.

**Unfunded studies**<br>Enter: *The author(s) received no specific funding for this work.*<br><br>**Funded studies**<br>Enter a statement with the following details:<br>• Initials of the authors who received each award<br>• Grant numbers awarded to each author<br>• The full name of each funder<br>• URL of each funder website<br>• Did the sponsors or funders play any role in the study design, data collection and analysis, decision to publish, or preparation of the manuscript?<br>• **NO** - Include this sentence at the end of your statement: *The funders had no role in study design, data collection and analysis, decision to publish, or preparation of the manuscript.*<br>• **YES** - Specify the role(s) played.<br><br>* typeset	
**Competing Interests**<br><br>Use the instructions below to enter a competing interest statement for this submission. On behalf of all authors, disclose any competing interests that could be perceived to bias this work—acknowledging all financial support and any other relevant financial or non-financial competing interests.<br><br>This statement is required for submission and **will appear in the published article** if the submission is accepted. Please make sure it is accurate and that any funding sources listed in your Funding Information later in the submission form are also declared in your Financial Disclosure statement.<br><br>View published research articles from *PLOS ONE* for specific examples.	The authors have declared that no competing interests exist.

Powered by Editorial Manager® and ProduXion Manager® from Aries Systems Corporation

**NO authors have competing interests**<br><br>Enter: *The authors have declared that no competing interests exist.*<br><br>**Authors with competing interests**<br><br>Enter competing interest details beginning with this statement:<br><br>*I have read the journal's policy and the authors of this manuscript have the following competing interests: [insert competing interests here]*<br><br>* typeset	
**Ethics Statement**<br><br>Enter an ethics statement for this submission. This statement is required if the study involved:<br><br>• Human participants<br>• Human specimens or tissue<br>• Vertebrate animals or cephalopods<br>• Vertebrate embryos or tissues<br>• Field research<br><br>Write "N/A" if the submission does not require an ethics statement.<br><br>General guidance is provided below. Consult the submission guidelines for detailed instructions. **Make sure that all information entered here is included in the Methods section of the manuscript.**	experiment is field research.

**Format for specific study types**<br><br>**Human Subject Research (involving human participants and/or tissue)**<br>• Give the name of the institutional review board or ethics committee that approved the study<br>• Include the approval number and/or a statement indicating approval of this research<br>• Indicate the form of consent obtained (written/oral) or the reason that consent was not obtained (e.g. the data were analyzed anonymously)<br><br>**Animal Research (involving vertebrate animals, embryos or tissues)**<br>• Provide the name of the Institutional Animal Care and Use Committee (IACUC) or other relevant ethics board that reviewed the study protocol, and indicate whether they approved this research or granted a formal waiver of ethical approval<br>• Include an approval number if one was obtained<br>• If the study involved *non-human primates*, add *additional details* about animal welfare and steps taken to ameliorate suffering<br>• If anesthesia, euthanasia, or any kind of animal sacrifice is part of the study, include briefly which substances and/or methods were applied<br><br>**Field Research**<br><br>Include the following details if this study involves the collection of plant, animal, or other materials from a natural setting:<br>• Field permit number<br>• Name of the institution or relevant body that granted permission	
**Data Availability**<br><br>Authors are required to make all data underlying the findings described fully available, without restriction, and from the time of publication. PLOS allows rare exceptions to address legal and ethical concerns. See the PLOS Data Policy and FAQ for detailed information.	Yes - all data are fully available without restriction

A Data Availability Statement describing where the data can be found is required at submission. Your answers to this question constitute the Data Availability Statement and **will be published in the article**, if accepted.<br><br>**Important:** Stating 'data available on request from the author' is not sufficient. If your data are only available upon request, select 'No' for the first question and explain your exceptional situation in the text box.<br><br>Do the authors confirm that all data underlying the findings described in their manuscript are fully available without restriction?	
**Describe where the data may be found in full sentences. If you are copying our sample text, replace any instances of XXX with the appropriate details.**<br><br>• If the data are **held or will be held in a public repository**, include URLs, accession numbers or DOIs. If this information will only be available after acceptance, indicate this by ticking the box below. For example: *All XXX files are available from the XXX database (accession number(s) XXX, XXX.)*.<br>• If the data are all contained **within the manuscript and/or Supporting Information files**, enter the following: *All relevant data are within the manuscript and its Supporting Information files.*<br>• If neither of these applies but you are able to provide **details of access elsewhere**, with or without limitations, please do so. For example:<br><br>*Data cannot be shared publicly because of [XXX]. Data are available from the XXX Institutional Data Access / Ethics Committee (contact via XXX) for researchers who meet the criteria for access to confidential data.*<br><br>*The data underlying the results presented in the study are available from (include the name of the third party*	all nessary dats avilable in the manuscript

Powered by Editorial Manager® and ProduXion Manager® from Aries Systems Corporation

and contact information or URL).<br>• This text is appropriate if the data are owned by a third party and authors do not have permission to share the data.<br><br>* typeset	
Additional data availability information:	Tick here if your circumstances are not covered by the questions above and you need the journal's help to make your data available.

Powered by Editorial Manager® and ProduXion Manager® from Aries Systems Corporation

Manuscript

Click here to access/download;Manuscript;Manuscript best edited.docx

Effects of dry-bio-slurry with nitrogen fertilizer on yield of potato and wheat under short term crop rotation

Zelalem Addis*[1], Tadele Amare[1], Bitewlgn Kerebih[1], Antenh Abewa[1], Tesfaye Feysa[2,] Abrham Awoke[1] and Abere Tenagne[1]

1 Adet Agricultural Research Center P.O.BOX 08, Bahir dar Ethiopia

2 Amhara Agricultural Research Institute

*Corresponding Author zelalemaddis660@gmail.com

ABSTRACT

Effective cropping system with nutrient balancing is the key solution in farming practices by improving of nutrient use efficiency, reduce need for inorganic fertilizers and impact of pests and diseases. The experiment was conducted to fill the gap of using balanced nutrient in crop rotation system for the improvement of both soil properties and crop yields. That was done by substitution balancing ratio of dry bio-slurry and nitrogen fertilizers for the enhancement of soil properties and potato-wheat yields. The experiment was contain ten levels of treatments Control (0.0), RN (recommended nitrogen), 50% DBS (dry-bio slurry), 100% DBS (dry-bio slurry), 75% DBS (dry-bio slurry), 75% DBS (dry-bio slurry)+25% RN (recommended nitrogen), 50% DBS (dry-bio slurry)+50% RN (recommended nitrogen), 25% DBS (dry-bio slurry)+75% RN (recommended nitrogen), 100% DBS (dry-bio slurry) +25% RN (recommended nitrogen) and 100% DBS (dry-bio slurry)+50% RN (recommended nitrogen) that were laid out in RCBD design with three replications for three years. Data on soil Physico-chemical properties and yield components of potato and wheat were collected and subjected to ANOVA using SAS software 9.4. The results were indicated that; an application of dry bio-slurry with nitrogen fertilizer was significantly affected the yield and yield components of both potato and wheat in their rotation system. Soil

properties also, significantly changed by the residual effect of dry bio-slurry. Applying 25% DBS with 75 % RN was given the highest tuber yield of potato (27.6 tha-1) as compared to control. Similarly, an application of 100% and 75% sole dry bio-slurry scored the highest grain yield (3.85tha^{-1}) and above-ground biomass (9.59tha^{-1}) of wheat respectively. The combination of 25% DBS with 75% RN also, recorded the highest net benefit (2889.2 US$) with acceptable marginal return (4486.2%). So that, to attain the highest net benefit, application of 25% DBS with 75% RN could be promoted for yield improvement in the study area and similar agroecology.

Key Words: dry bio-slurry, nitrogen, soil properties, potato, wheat

1. Introduction

A well-planned cropping system can enhance nutrient use efficiency, reduce the need for inorganic fertilizers and lessen the impact of pests and diseases (1&2). Potato and wheat production under a crop rotation system is commonly practiced by farmers in the Amhara region. The system comprises a cradle-to-farm analysis for 3-years rotation cycle. It allows mitigating the impact of certain pests and diseases, which significantly affects the yield of the main crop potato. In addition to this, it enables to take full advantage of residual fertility that comes from potato leaves entire decomposition in the soil. This rotation system was chosen by many producers and it is considered as the most efficient system from an economic, quality and soil health point of view. Potato (*Solanum tuberosum* L.) is the third most important food crop after wheat and rice in the world (3). In Ethiopia, the area coverage under potato cultivation reaches about 73,677.64 ha and its production was estimated around.1,044,436.359 tons (4). The productivity of potato in Ethiopia is reached about 13.9 t ha^{-1} (5); which is relatively low compared to other African countries (6). Bread wheat (*Triticum aestivum* L.) is also is one of the major cereal crops grown in the highlands

of Ethiopia, and it makes the country is regarded as the largest wheat producer in Sub-Saharan Africa (7).

Out of the total grain crop area coverage, wheat ranked 4th after tef (*Eragrostis tef*), maize (*Zea mays*) and sorghum (*Sorghum bicolor*) while, it is the third in total production after maize and tef (5). Despite the long history of wheat cultivation and its importance to Ethiopian agriculture, its average yield also still very low, not exceeding 2.4 t ha^{-1} (5). Which is below the world's average yield of wheat 3.4 t ha^{-1} (6). This phenomenon is common in the Amhara region. Low level of potato-wheat productivity is mainly due to soil fertility degradation, improper fertilization, poor pest management practices, use of the low-quality seed and soil nutrient depletion (8). Enhancing soil fertility is the first precondition for a practical crop production system; that can be achieved through the application of organic manure (like bio-slurry) and inorganic fertilizers integration for sustainable crop productivity (9 & 10). Different studies also indicated that judicious application of both organic and inorganic fertilizers is a key solution for crop productivity and soil fertility enhancement. The study conducted by (11) revealed that integrated application of both organic and inorganic source fertilizers significantly improved yield and soil fertility status under maize - potato cropping system .Another study which done by (12) indicated that the supplement of tricho compost with chemical fertilizer and vermicompost with chemical fertilizer significantly affected the yield of potato,mungbean and T.aman rice by scoring of 6.3-33.7%,8.3-33.8% and 2.9-18.3% respectively over sole application treatment .Similarly the study conducted by (42) also observed that poultry manure bio slurry, poultry manure, cow dung bio slurry, and cow dung gave 11.7, 8.9, 5.4, and 3.1% respective increase in total system productivity over sole chemical fertilizer (46% N, as urea, 20% P as triple superphosphate, 50% K as muriate of potash and 18% S as gypsum).Bio-slurry that obtained after extraction of the energy content of animal manure is an

excellent fertilizer, rich in major nutrients (nitrogen, phosphorous and potassium) and organic matter which is important for soil fertility and yield of crops (13). It also improves the physical and biological quality of soil beyond its role in the providing of plant nutrients. In addition, an application of bio-slurry can help in the reduction of dependence on mineral fertilizers (14). Because of nitrogen is the most limiting essential nutrient for growth and development of crops, both potato and wheat are highly responsive to N fertilization (15). Furthermore; supplying of nitrogen fertilizer plays an important role in the balance between vegetative and reproductive growth of crops (16&17). Various studies showed that N fertilizer applications can increase the dry matter protein content of wheat and potato tubers (18). Moreover, most of the time the available nitrogen concentration in most organic source fertilizers including bio-slurry is high as compared to other major nutrients (13) Due to this the application of dry bio-slurry with nitrogen fertilizer through nitrogen equivalence balancing ratio is very important. Beside this, neither organic manure nor chemical fertilizer alone can meet the nutrient demand of a given crop and also their own negative impact in terms of sustainable crop and soil productivity in different cropping system (19). For instance, continuous addition of chemical fertilizers can cause soil quality deterioration such as increasing of soil acidity, loss of organic matter and depletion of nutrients that are not supplied in the fertilizer formulation (45). On the other hand, organic fertilizers are required in large quantities and their nutrients released slowly that make less efficient on soil properties and crop yields within short period of time as compared to inorganic fertilizers (46). Because of these reasons, the study was conducted to determine the main and residual effects of dry-bio slurry and nitrogen fertilizers on soil and yield of potato and wheat under crop rotation system at Yilemana Densa District in North Western Amhara Region Ethiopia

2. Material and methods

2.1 Description of Study Area

The experiment was conducted at Yilemana Densa district on Farmer's field across three sites (Y1S1, Y2S1and Y2S2) for three consecutive years (2019-2022) as potato and wheat rotation (potato-wheat cropping system) at Debremewi Keble West Gojam Zone North Western Amhara Region of Ethiopia. Geographically the area lies at 11^{0} 21' 22'' N and 37^{0} 25' 43'' E (Figure 1) with a mean altitude of 2304 m above sea level. It receives a mean annual rainfall of 1421 mm with mean minimum and maximum temperatures of 12.29 and 27.56 °C, respectively. The landforms of the area are characterized by undulating to rolling plateaus, scattered moderate hills, dissected side slopes, and river gorges (20). Based on the District Bureau of Agriculture, the major land use comprises cultivated land (57%), forest and bushes (2%), grazing land (33%), and others (8%). Major crops, grown in the study area are Maize, Tef, wheat, Barley, Potato, and Field pea. Soil types in the area are Nitisols (45%) Vertisols (30%) and Luvisols (25%). This on-farm experiment was conducted on Luvic Nitisols which is the most dominant soil in the study area

2.2 Experimental procedure

The experiment was contain ten treatments which includes control (without N and DBS), recommended N (138 N) ,dry-bio slurry 50% equivalence (5.3 tha^{-1}), dry-bio slurry 100% equivalence (10.6 tha^{-1}), dry-bio slurry 75% equivalence (7.95 tha^{-1}), dry-bio slurry 75% equivalence (7.95 tha^{-1}) + 25% recommended N (34.5 N), dry-bio slurry 50% equivalence (5.3 tha^{-1}) + 50% recommended N (69 N), dry-bio slurry 25% equivalence (2.65 tha^{-1}) + 75% recommended N (103.5 N), dry-bio slurry 100% equivalence (10.6 tha^{-1})+25% recommended N (34.5 N) and dry-bio slurry 100% equivalence (10.6 tha^{-1}) +50% recommended N (69 N) that were laid out in Randomized Complete Block Design (RCBD) with three replications. The rates of dry-bio slurry were adjusted based on potato nitrogen recommended rate 138 kgha^{-1} N (47)

equivalency corresponding to its N content. Urea was used as a source of synthetic 138N whereas, phosphorous at the rate of 69 kgha^{-1} P_2O_5 was applied in the form of TSP to all plots uniformly. The experiment was carried out under rain-fed conditions GUDENIE and TAY varieties were used as test crops for potato and wheat respectively in each rainy season one after the other. Total area of each plot was 4.5 m x 3 m (13.5 m^2) having 1 m space between plots and 1.5 m between blocks. Potato was spaced by 0.3 m between plants & 0.75 m between rows and the data were collected from the middle four rows. Whereas; Wheat was planted as a rotated crop in 0.2 m and harvested from middle rows by avoiding four rows as a border.

2.2.1 Dry bio-slurry

It was collected from farmers biogas plants and stored under well-protected shade for better drying and avoiding nutrient loss especially nitrogen. After drying, we make it fine and break into small pieces for nice application and soil reaction. After this; during planting period of time dry-bio slurry was incorporated into the soil. Representative composite sample was taken from the whole collected dried pit of slurry for analysis of hydrogen concentration (pH), organic carbon (OC%), cation exchange capacity (CEC), total nitrogen (TN%) and available phosphorus (P) by following laboratory manual procedures (21).

Table 1. Physico-chemical analysis of dry-bio slurry before incorporation in year1and 2 and its average

	Dry bio-slurry	Concentration
**Year 1**		
	Dry matter%	11.2
	OC%	27.8
	TN%	1.2
	Av P (mg kg^{-1})	112.1
	pH (H_2O; 1:2.5)	7.9
	C:N ratio	22.9
	CEC (cmol kg^{-1})	59.8
**Year 2**		
	Dry matter%	11.8

OC%	16.3
TN%	1.4
Av P (mg kg^{-1})	91.6
pH (H_2O; 1:2.5)	7.7
C:N ratio	11.6
CEC (cmol kg^{-1})	67.4
**Average result of Year1andYear 2**	
Dry matter%	11.5
OC%	22.1
TN%	1.3
Av P (mg kg^{-1})	101.9
pH (H_2O; 1:2.5)	7.8
C: N ratio	17.3
CEC (cmol kg^{-1})	63.6

OC%=organic carbon percent, TN%=total nitrogen percent, C: N=carbon to nitrogen ratio, CEC = cation exchange capacity, AvP=available phosphorus, and pH= Power of hydrogen concentration.

2.2.2 Experimental crops

Gudenie variety is used as attest crop for potato and it was planted as main crop or precursor crop of wheat. TAY variety of wheat also planted in the next cropping season after potato which was used for the determination of dry bio slurry residual effect in potato-wheat rotation system. TSP (P_2O_5) was applied during the planting period of time as basal whereas inorganic N from urea was applied in three splits for potato; one-third third at planting, one-third 30 days after planting, and the remaining one-third at the beginning of the flowering. While for wheat recommended NP was applied in all plots except control which only received P_2O_5 to see the residual effect of dry bio-slurry in the next crop and nitrogen was supplied, in two splits.

2.3 Data Collection, Preparation, and Analysis

2.3.1 **Soil sampling and analysis of before planting**

Before planting, representative soil samples were collected from 0-20 cm depth in the sites via random sampling method from 10 spots using an auger. All samples were mixed together to form one composite sample. The sample was grounded by using a mortar and passed through a 2 mm sieve for analysis of soil texture, CEC, pH, and available P whereas ;a 0.5 mm sieve was used for

determining the organic carbon (OC) and total N.While, bulk density was determined by the core sampling method.

2.3.2 Soil sampling and analysis after harvesting of potato and wheat

The major chemical properties of soil such as OC, pH, CEC, total N and available P were analyzed following the compiled laboratory manual of (21). Soil pH was measured in water with the ratio of 1:2.5 using glass electrode pH meter. The soil OC content was determined following the wet digestion method as outlined by Walkley and Black which involves the digestion of soil OC with potassium dichromate ($K_2Cr_2O_7$) in a sulphuric acid solution. AvP was determined by Olsen extracting method. The total N content in the soil samples was determined following the Kjeldahl method. CEC also, determined by extracting the soil samples with ammonium acetate (1NNH_4OAc) followed by repeated washing with ethanol (96%) to remove the excess ammonium ions in the soil solution. Percolating the $NH4^+$ saturated soil with sodium chloride would displace the ammonium ions adsorbed in the soil and the ammonium liberated from the distillation was titrated using 0.1N NaOH.

2.3.3 Crop data collection

Plant height for both potato and wheat was measured at the maturity stage by taking five randomly selected plants from ground to the top apex and averaging for a single reading. Similarly, spike length of wheat was measured at the maturity stage by taking five randomly selected plants from staring point of spike grain to the end of spike grain and averaging for a single reading. Number of plants per hill (stem number per hill) was recorded by counting the stems that were directly sourced from original seed tuber of five hills of potato at 70% maturity stage.

Number of tubers per plant also taken at the maturity stage by counting potato tubers from five randomly selected plants in the middle four rows of experimental plots (7.2 m^2) and averaged for

single reading. While thousand seed weight for wheat was taken from each treatment harvestable plot (11.7 m^2) by counting of 1000 seeds at maturity stage after threshing and make them weighting under sensitive balance for weight reading. Total tuber yield of potato was measured by using balancing balance during harvesting period of time from the middle four rows (7.2 m^2) for total weight reading. Grain yield, and above-ground biomass of wheat was measured by using balancing balance at maturity stage from the net harvestable plot area (11.7 m^2).

2.4 Economic Analysis

The economic analysis was performed to make rational choice among the applied variables in the production of potato. The partial budget and marginal rate of return (MRR) were used for evaluating the change in farming methods that affect partially rather than the whole farm practice and also concerned with planning tool to estimate the profit change within a farm (22). This was computed by adjusting yield downward by a 10% and multiplying it with the local field price (6 Ethiopian Birr or 0.12 US$ per kg of potato). Dominance analysis was done by listing of treatments in an increasing order of cost and that has net benefit less than or equal to treatments with the lower costs that vary is dominated (22).

2.5 Statistical Analysis

All collected data's was subjected to analysis of variance through GLM procedure by using SAS software program version 9.4 (SAS Institute, 2014). List significant difference (LSD) at 0.05 probability level was employed to differentiate treatments means t (23).

3. Results and discussion

3.1 Soil chemical properties before planting and after harvesting of potato

The results of soil chemical properties before and after harvest from each experimental sites were displayed in Table 2, 3 & 4. Before-planting the soil was acidic in reaction with a pH (H_2O 1:2.5)

value of 5.1 Y1S1 (year one site one) and 5.2 Y2S1 (year two site one), even if, it was within the range of soil pH for potato production (24) (Table 2). The total N, available P, OC, C: N ratio and CEC of the soil Y1S1 and Y2S1 before planting were 0.16&0.16%, 6.9&6.3 mg kg^{-1}, 1.4 &1.5%, 11.1&9.3 and 33.9&30.3 cmol (+) kg^{-1}, respectively (Table 2). The total N content of the soil was within the range of medium according to (24) who was classified the range of total N < 0.1, 0.1-0.15, 0.15-0.25 and > 0.25% as very low, low, medium and high, respectively. (25) also, classified the range of soil available P content was like this < 5 as very low, 5 -15 as low, 15 -25 as medium and > 25 mg kg^{-1} as high. Hence the available P of the soil before planting lies under the low range. According to (26) the soil OC content ranges was rated 1-2, 2-4, and 4-6% as low, medium and high, respectively. Similarly; cation exchange capacity (CEC) was also rated with the ranges of 5-15, 15-25 and 25-40 cmol kg^{-1} as low, medium and high, respectively (26).

Based on these ratings OC (1.4, 1.5 &2.0%) and CEC (33.9, 30.3&30.9 cmol kg^{-1}) before planting of the experimental fields (Y1S1, Y2S1 and Y2S2) were in the low and high ranges, respectively. Generally, the nutrient contents of the study site Y2S1 is not good in terms of availability of major plant nutrients beside its nice CEC. On the other hand, on Y2S2 pre planting values of pH, TN, AvP, OC, C: N and CEC were 5.5, 0.17%, 6.8 ppm, 2.4%, 14.1 and 33.9 respectively. Based on (24) rating the pH value was under moderate while total nitrogen was medium. Similarly, according to (25) available P was under low while OC and CEC were medium and high respectively (26). Y2S2 rather than the two sites (Y1S1 and Y2S1) had a good soil fertility status based on its soil chemical properties. However, the acidity of soil Y1S1andY2S2 may be cause the sorption of available P. Thus, application of OM like bio-slurry is very essential in order to neutralize soil solution for the availability of nutrients. On the other hands, after harvest all soil

chemical properties except soil pH (on Y2S2) were not affected by the application of dry bio-slurry with N equivalence rates (Table3).

The non-significant effects of the applied treatments on the rest soil properties might be the slow release of nutrients from dry bio-slurry that was applied during the experimentation period to soil solution. However, applied dry bio-slurry and N rates had a significant ($P < 0.05$) effect on soil pH at site two in 2020-21 as compared to the control (Table 2). Numerically the highest soil pH was obtained by the application of 100% dry bio-slurry and 100% dry bio-slurry +25% RN as compared to the control (Table 3). The increment of soil pH in treated plots may be related due to the releasing of basic cations from dry bio-slurry into soil solution that makes for the substitution of acid cations. These results were in agreed with the investigation of (27) they reported that the combined application of compost with inorganic NPSB on maize increased the soil pH content after harvest as compared to control.

3.2 Residual effect of dry bio-slurry on soil chemical properties after harvesting of wheat

The residual effect of dry bio-slurry on selected soil chemical properties was significantly explained at ($p < 0.05$) across sites and years in table 4. Related to this an addition of 100% (10.6tha^{-1}) dry bio-slurry with 50% RN (69 kg N) was given the highest TN% (0.205 and 0.223) on Y1S1 and Y2S2 respectively than control. This might be due to the gradual releasing of dry bios-slurry nitrogen into soil solution beyond its chelating capacity. The finding lined with (28) they found that an application of dry bio-slurry with inorganic fertilizers gives the maximum value of total nitrogen than control. Similarly, the study conducted by (29) also indicated that an application of 70cm^3 scored the highest (1.36%) total nitrogen as compared to control which was given 0.07%.

On the other hand, OC, C: N and CEC were significantly affected at Y2S1 (Table 4),While; in Y2S2 only CEC is significant at ($p < 0.05$) Table 4.Based on this; numerically the highest value of CEC (34.6), C: N (12.82) & OC% (2.20) was observed by application 50% dry bio slurry (DBS) and 100% dry bio slurry (DBS) with 25% RN as compared to control on Y2S1.On Y2S2 the maximum value of CEC ($cmolkg^{-1}$) 36.06 was obtained in plots that receive 100% DBS with 25%N than control. This might be the positive effect of applied DBS on the improvement of organic matter and soil holding capacity of positive cations on its exchangeable site. The finding agreed with (30) who revealed that an application of 41.3 m^{-3} liquid bio-slurry with 20.5 $kgha^{-1}$ N significantly increased soil organic carbon than untreated plots. The study conducted by (31) also indicated that an application of 10 tha^{-1} compost with 50% N $kgha^{-1}$ significantly increased OC% by scoring the maximum value of 0.67 than the control treatment. Organic amendments significantly enhanced SOC and they make a considerable effect on soil microbes abundance and nutrient availability and uptake this may alter the C: N ratio of the soil. However, the addition of external organic matter with a low C: N ratio may induce the mineralization of OM. This makes nitrogen trapped by organic matter; a phenomenon known as the priming effect (32). The application of the 50% DBS and 100% DBS with 25% N gives the highest CEC (34.67 & 36.06) at Y2S1 and Y2S2 respectively as compared to the control (Table 4). Such increment in CEC might be due to the application of DBS on soil, that makes the colloidal site of the soil negatively charged for storing of basic cations. The finding in agreed with (33) who reported that the use of organic FYM and inorganic fertilizers significantly increased CEC over the control.

Table 2. Initial soil properties of the experimental sites

**Texture SCL**	**Years**	**Initial soil properties across sites and years**						
		BD	pH	TN%	Av P (ppm)	CEC (cmol kg^{-1})	C: N	OC%
Sandy= 56%								
Clay = 32%	2019-20 (Y1S1	1.22	5.1	0.16	6.9	33.9	8.8	1.4
Loam =12%	2020-21 (Y2S1)	1.33	5.2	0.16	6.3	30.3	9.3	1.5
	2020-21 (Y2S2)	1.26	5.5	0.17	6.8	33.9	11.8	2.0

NB: SCL= sandy clay loam; BD= bulk density; Y1S1= year one site one, Y2S1= year two site one and Y2S2 = year two site two.

Table 3. Main effects of dry bio slurry with nitrogen on soil chemical properties after harvest of potato

**2019-20 (Y1S1)**						
**Treatment**	pH	TN%	Av P (ppm)	CEC (cmol kg^{-1})	C: N	OC%
Control (0,0)	5.13	0.12	9.3	29.1	11.6	1.4
RN (138 N $kgha^{-1}$)	5.11	0.12	7.8	29.9	11.61	1.3
50% DBS (5.3 tha^{-1})	5.19	0.13	8.8	29.6	11.59	1.4
100% DBS (10.6 tha^{-1})	5.18	0.13	9.6	29.7	11.58	1.5
75% DBS (7.95 tha^{-1})	5.19	0.13	10.2	29.1	11.62	1.5
75% DBS+25% N (7.95 tha^{-1}+34.5 N $kgha^{-1}$)	5.22	0.16	10.1	28.3	11.57	1.9
50% DBS+50% N (5.3 tha^{-1}+69 N $kgha^{-1}$)	5.10	0.15	9.1	28.7	11.59	1.7
25% DBS+75% N (2.65 tha^{-1}+103.5 N $kgha^{-1}$)	5.18	0.15	7.5	29.2	11.61	1.7
100% DBS+25% N (10.6 tha^{-1}+34.5 N $kgha^{-1}$)	5.12	0.13	12.3	28.3	11.62	1.5
100% DBS+50% N (10.6 tha^{-1}+69 N $kgha^{-1}$)	5.15	0.13	11.2	27.6	11.60	1.5
**LSD**	**NS**	**NS**	**NS**	**NS**	**NS**	**NS**
**CV%**	**1.2**	**14.3**	**22.43**	**3.3**	**0.21**	**14.19**
**2020-21 (Y2S1)**						
**Treatment**	pH	TN%	Av P (ppm)	CEC (cmol kg^{-1})	C: N	OC%

Control (0,0)	5.2	0.15	4.4	27.3	11.59	1.7
RN (138 N $kgha^{-1}$)	5.3	0.16	4.3	27.7	11.59	1.8
50% DBS (5.3 tha^{-1})	5.3	0.14	6.5	30.8	11.61	1.7
100% DBS (10.6 tha^{-1})	5.4	0.15	6.2	27.4	11.60	1.8
75% DBS (7.95 tha^{-1})	5.4	0.15	5.8	28.6	11.58	1.7
75% DBS+25% N (7.95 tha^{-1}+34.5 N $kgha^{-1}$)	5.3	0.14	5.4	24.4	11.63	1.7
50% DBS+50% N (5.3 tha^{-1}+69 N $kgha^{-1}$)	5.3	0.14	4.5	26.7	11.61	1.6
25% DBS+75% N (2.65 tha^{-1}+103.5 N $kgha^{-1}$)	5.2	0.14	4.6	27.5	11.59	1.7
100% DBS+25% N (10.6 tha^{-1}+34.5 N $kgha^{-1}$)	5.4	0.17	4.4	28.4	11.59	1.9
100% DBS+50%N (10.6 tha^{-1}+69 N $kgha^{-1}$)	5.4	0.16	4.4	28.5	11.61	1.9
**LSD**	**NS**	**NS**	**NS**	**NS**	**NS**	**NS**
**CV%**	**2.12**	**13.5**	**21.6**	**9.0**	**0.17**	**13.5**
	**2020-21 (Y2S2)**					
**Treatment**	pH	TN%	Av P (ppm)	CEC (cmol kg^{-1})	C: N	OC%
Control (0,0)	5.3^{cd}	0.17	12.2	30.6	12.10	2.1
RN (138 N $kgha^{-1}$)	$5.4b^{cd}$	0.17	7.1	31.9	11.60	1.9
50% DBS (5.3 tha^{-1})	5.5^{abc}	0.18	11.5	30.4	11.60	2.0
100% DBS (10.6 tha^{-1})	5.6^{ab}	0.18	9.5	32.1	12.62	2.3
75% DBS (7.95 tha^{-1})	5.6^{ab}	0.17	13.2	32.1	12.00	2.0
75% DBS+25% N (7.95 tha^{-1}+34.5 N $kgha^{-1}$)	5.5^{abc}	0.18	13.3	33.5	11.60	2.1
50% DBS+50% N (5.3 tha^{-1}+69 N $kgha^{-1}$)	5.5^{abcd}	0.17	13.1	32.6	12.01	2.1
25% DBS+75% N (2.65 tha^{-1}+103.5 N $kgha^{-1}$)	5.3^{d}	0.16	9.5	30.9	11.60	1.9
100% DBS+25% N (10.6 tha^{-1}+34.5 N $kgha^{-1}$)	5.6^{a}	0.18	11.6	31.8	11.80	2.1
100% DBS+50% N (10.6 tha^{-1}+69 N $kgha^{-1}$)	5.4^{bcd}	0.17	15.3	33.4	11.60	2.1
**LSD**	**2.3**	**NS**	**NS**	**NS**	**NS**	**NS**
**CV%**	**0.22**	**9.9**	**29.1**	**6.1**	**5.9**	**13.8**

Means followed by the same letter (s) within the column are not significantly different at ($P \leq 0.05$). control = is no nitrogen and dry bio slurry, RNP=percent of recommended nitrogen and phosphorus, DBS =dry bio-slurry, pH= power of hydrogen concentration, TN% = total nitrogen percent, AvP = available phosphorus, OC% = organic carbon percent, C: N ratio = carbon to nitrogen ratio, CEC = cation exchange capacity, OM% = organic matter percent, Y1S1= year one site one, Y2S1= year two site one and Y2S2 = year two site two.

Table 4. The residual effect of dry bio-slurry on soil chemical properties after wheat harvesting in the potato-wheat cropping system

Treatment	pH	TN%	Av P (ppm)	CEC (cmol kg^{-1})	C: N	OC%
**2020-21 (Y1S1)**						
Treatment	pH	TN%	Av P (ppm)	CEC (cmol kg^{-1})	C: N	OC%
Control (0,0)	5.40	0.174bcd	8.97	21.39	9.68	1.69
RN (138 N kgha^{-1})	5.40	0.196a	10.12	21.69	10.01	1.95
50% DBS (5.3 tha^{-1})	5.40	0.197a	9.25	26.13	9.64	1.88
100% DBS (10.6 tha^{-1})	5.38	0.168d	11.04	23.51	11.81	1.98
75% DBS (7.95 tha^{-1})	5.40	0.192ab	9.34	25.60	9.42	1.82
75% DBS+25% N (7.95 tha^{-1}+34.5 N kgha^{-1})	5.42	0.172cd	9.07	24.51	11.98	2.06
50% DBS+50% N (5.3 tha^{-1}+69 N kgha^{-1})	5.36	0.188abc	10.06	22.09	9.61	1.80
25% DBS+75% N (2.65 tha^{-1}+103.5 N kgha^{-1})	5.35	0.202a	10.08	25.11	10.14	2.04
100% DBS+25% N (10.6 tha^{-1}+34.5 N kgha^{-1})	5.31	0.194ab	10.46	21.81	11.01	2.13
100% DBS+50% N (10.6 tha^{-1}+69 N kgha^{-1})	5.37	0.205a	10/76	23.78	9.35	1.89
**LSD**	NS	0.020	NS	NS	NS	NS
**CV%**	1.3	6.2	12.7	11.9	14.8	13.3
**2021-22 (Y2S1)**						
Treatment	pH	TN%	Av P (ppm)	CEC (cmol kg^{-1})	C: N	OC%
Control (0,0)	5.61	0.178	10.15	27.70d	7.61d	1.36b
RN (138 N kgha^{-1})	5.53	0.179	10.22	30.52bc	10.31abc	1.85a
50% DBS (5.3 tha^{-1})	5.69	0.180	11.56	34.67a	12.25abc	2.20a
100% DBS (10.6 tha^{-1})	5.67	0.160	9.85	31.77bc	12.42ab	1.98a
75% DBS (7.95 tha^{-1})	5.64	0.194	9.85	31.09bc	9.74cd	1.88a
75% DBS+25% N (7.95 tha^{-1}+34.5 N kgha^{-1})	5.70	0.178	11.76	29.56cd	10.79abc	1.90a
50% DBS+50% N (5.3 tha^{-1}+69 N kgha^{-1})	5.62	0.167	10.69	30.63bc	11.38abc	1.90a
25% DBS+75% N (2.65 tha^{-1}+103.5 N kgha^{-1})	5.54	0.186	9.59	30.37bc	10.00bcd	1.86a
100% DBS+25% N (10.6 tha^{-1}+34.5 N kgha^{-1})	5.58	0.172	9.88	31.99bc	12.82a	2.20a
100% DBS+50% N (10.6 tha^{-1}+69 N kgha^{-1})	5.44	0.185	10.19	32.91ab	11.78abc	2.17a
**LSD**	NS	NS	NS	2.60	2.61	0.41
**CV%**	2.0	6.7	14.4	4.8	14.1	12.6
**2021-22 (Y2S2)**						
Treatment	pH	TN%	Av P (ppm)	CEC (cmol kg^{-1})	C: N	OC%

Control (0,0)	5.51	0.176de	10.03	29.43c	12.03	2.12
RN (138 N kgha^{-1})	5.65	0.190bcd	8.76	30.69bc	12.19	2.29
50% DBS (5.3 tha^{-1})	5.62	0.186bcde	13.49	35.92 a	12.78	2.38
100% DBS (10.6 tha^{-1})	5.62	0.209ab	14.08	33.68ab	11.63	2.43
75% DBS (7.95 tha^{-1})	5.76	0.178cde	11.74	34.47a	13.41	2.39
75% DBS+25% N (7.95 tha^{-1}+34.5 N kgha^{-1})	5.68	0.207ab	12.93	34.81a	12.29	2.54
50% DBS+50%N (5.3 tha^{-1}+69 N kgha^{-1})	5.57	0.202abc	13.08	35.88a	11.47	2.30
25% DBS+75% N (2.65 tha^{-1}+103.5 N kgha^{-1})	5.59	0.164e	11.07	33.27ab	13.52	2.19
100% DBS+25% N (10.6 tha^{-1}+34.5 N kgha^{-1})	5.66	0.223a	11.51	36.06a	10.09	2.24
100% DBS+50% N (10.6 tha^{-1}+69 N kgha^{-1})	5.66	0.207ab	11.21	35.95a	11.75	2.42
**LSD**	NS	0.025	NS	3.78	NS	NS
**CV%**	2.3	7.6	17.8	6.5	10.5	7.3

Means followed by the same letter (s) within the column are not significantly different at ($P \leq 0.05$). RNP=percent of recommended nitrogen and phosphorus, DBS =dry bio-slurry, pH= power of hydrogen concentration, TN% = total nitrogen percent, AvP = available phosphorus, OC% = organic carbon percent, C: N ratio = carbon to nitrogen ratio, CEC = cation exchange capacity, OM% = organic matter percent, Y1S1= year one site one, Y2S1= year two site one and Y2S2 = year two site two.

3.2 Effects of dry bio slurry and nitrogen on yield and yield components of potato and wheat

3.2.1 Plant height and number of Stem per hill of potato

Integration of dry bio slurry with nitrogen (N) was significantly ($P < 0.05$) affects plant height (Table 5). Numerically,the highest values of plant height (52.1 and 48.8 cm) was achieved with addition of recommended nitrogen (RN) and 25% DBS with 75% N recpectively as compare control (Table 5).Increasing of plant height in response to DBS with N fertilizer may be due to the improvement of physico - chemical properties of the soil interms of water absorption for nutrient utilization of the plant. Moreover, DBS may deliver balanced micro and macronutrients as well as it was enhanced the availability of plant nutrients,which would help to speed up the metabolic activity of microorganisms and promote plant growth.The result in agreed with the findings of (34) who observed the longer plants when potato were applied with farm yard manure (13.5 tha^{-1}) and NPS (245.1 $kgha^{-1}$).lt is also harmonized with the findings of (35) who recorded maximum plant height of french bean from the application of 120 kg N ha^{-1} while, the minimum value was obtained from the control treatment. Another study that was conducted by (36) also,reflected that the highest value of mung bean plant height (78.08 cm) was recorded from the treatment which received 20:50 NP kg ha^{-1} with inoculation of Rhizobium as compared to the lowest value 68 cm on control treatment. On the other hand, a combined analysis of variance revealed that DBS with N fertilizers had not significant effects on the stem numbers of potato per hill (Table 5). This may be due to the parameter more favors for genetic makeup, physiological age and tuber seed size rather than a nutrient supplement. This finding line with the study of (37) who observed that shoot number of potato is mostly determined by the genetic makeup, the physiological age, and the size of potato seed tubers rather than mineral nutrients added in the form of fertilize

3.2.2 Plant height and spike length of wheat

Over years residual analysis of dry-bio slurry on wheat indicated that it was significantly affected both plant height and spike length at ($P < 0.05$) in (Table 5). The respective highest values of plant height and spike length (93.2 and 9.3 cm) were achieved by application of 100% DBS as compared to untreated plots (Table 5). This might be from the positive effect of DBS for delivering balanced micro and macronutrients as well as enhanced availability of plant nutrients via improving of soil properties. The result agreed with the findings of (38) who observed that the longer plants in plots which are received 75% cow dung with 25 % vermicompost than un-treated plots .lt is also harmonized with the findings of (39) who recorded that the maximum plant height (101.5 cm) value of Boro rice from the application of 5 tha^{-1} trichocompost as compared to control that was scored the minimum value (78.6 cm) . Similarly, the result of spike length also much with the study of (40) who said that an application of 15 tha^{-1} bio-gas slurry gives the highest spike length of wheat than checked treatment or control

Table 5. Main and residual effects of dry bio-slurry with equivalence N on growth parameters of potato and wheat

**Treatments**	**Main effect of DBS on Potato**		**Residual effect DBS on Wheat**	
	**PH (cm)**	**NSPH**	**PH (cm)**	**SL (cm)**
Control (0,0)	29.4^{de}	3.3	68.2d	6.7d
RN (138 N kgha^{-1})	52.1^{a}	4.8	89.1c	8.5c
50% DBS (5.3 tha^{-1})	28.2^{e}	4.4	92.3ab	8.7bc
100% DBS (10.6 tha^{-1})	30.4^{de}	4.4	93.2a	9.3a
75% DBS (7.95 tha^{-1})	36.6^{cd}	4.5	93.0ab	9.0abc
75% DBS+25% N (7.95 tha^{-1}+34.5 N kgha^{-1})	41.9^{bc}	4.6	92.1abc	9.0abc
50% DBS+50% N (5.3 tha^{-1}+69 N kgha^{-1})	42.7^{bc}	3.7	90.2bc	8.9abc
25% DBS+75% N (2.65 tha^{-1}+103.5 N kgha^{-1})	48.8^{ab}	4.0	90.2bc	8.7bc
100% DBS+25% N (10.6 tha^{-1}+34.5 N kgha^{-1})	44.0^{bc}	4.3	92.1ab	9.0abc
100% DBS+50% N (10.6 tha^{-1}+69 N kgha^{-1})	47.1^{ab}	4.2	90.6abc	9.1ab
**LSD**	**7.9**	**NS**	3.0	0.5
**CV%**	**20.9**	**30.1**	3.6	6.6

Means followed by the same letter (s) within the column are not significantly different at ($P \leq 0.05$). DBS= dry bio slurry, N= nitrogen, PH= plant height, NSPH = number of stems per hill, SL=spike length.

3.2.3 Number of tubers per plant and total tuber yield of potato

The combined analysis of result across years and sites indicated that yield and yield a component of potato was significantly differ at (P < 0.05) by the effects of DBS and RN (Table 6). The application of 75% N with 25% DBS gives the highest fresh total tuber yield (27.6 tha^{-1}) while the lowest fresh tuber yield (8.6 tha^{-1}) was observed at control. This might be due to the releasement of nitrogen (N) from dry bio-slurry (DBS) and Urea to soil solution that makes for plant better growth and development. Moreover, it could be due to the addition of both macro and micro nutrients from the dry bio-slurry (DBS) by improving of soil pH, organic carbon, total nitrogen phosphorus and cation exchange capacity. This study is in line with the findings of (13) who revealed that, the supplying of recommended inorganic fertilizer (100kg DAP, 50kg Urea and 50kg Murate potash per hectare) with 8 tha^{-1} bio-slurry gives maximum (266.7 tha^{-1}) yield of cabbage as compare to the lowest (160 tha^{-1}) from the control treatment. And it gives about 66.7% yield increment due to the combination both bio-slurry and recommended fertilizers over control. On the other hand, the study done by (41) indicated that the lowest value of fresh shoot biomass and marketable yield of potato tuber were achieved from control while the highest values were obtained in plots that treated combined application of farm yard manure and recommended nitrogen and phosphorus.

Similarly, number of tubers per plant (NTPP) was significantly affected at (P < 0.05) by the application DBS with equi-valence nitrogen. Maximum value of NTPP was observed through the application of recommended NP as compare to control treatment (Table 6). Even if the maximum value occurs at recommended NP, the combination of both dry bio slurry and nitrogen treatment also gives better yield advantage than control. This might be due to; the harmonization of organic and inorganic fertilizers for uptake and assimilation of nutrients to potato tubers by increasing the

availability of native soil nutrients through higher biological activities. The result coincides with study of (42) who observed that an application of organic and inorganic fertilizers increased the number of tubers per plant in treated plots than control or untreated plots. Another study done by (28) showed that an application of dry bio-slurry with recommended nitrogen and phosphorus can increased the number of fruits per plant of tomato up to 40 to 73% than control

3.2.4 Grain yield and aboveground biomass of wheat

Yield and aboveground biomass of wheat was significantly affected at ($P < 0.05$) in (Table 6) by residual effect of dry bio slurry. The application of 100% DBS gives the highest grain yield (3.85 tha^{-1}) as compare to control. Similarly, addition of 75% DBS gives the highest aboveground biomass of wheat (9.59 tha^{-1}) as compare to control (Table 6). Releasing N from dry bio-slurry (DBS) to soil solution may contribute for plant better growth and development. Moreover, it could be due to the addition of both macro and micro nutrients from the dry bio-slurry in to soil solution (rhizosphere) by increasing the availability of native soil nutrients. The result coincides with findings of (40) who reported that the supplement of 10 and 15 tha^{-1} of bio slurry significantly increased the grain yield of wheat as compared to control. Similarly, study done by (43) revealed that the application of both biogas slurry and chemical fertilizer at 50% has a good strategy for sustainable crop yield production and soil health improvement. Similarly, the study conducted by (44) also showed that an addition of 100% dry bio-slurry significantly increased the stover and stalk yield of maize by 45.5 and 42.2% respectively than control treatment via improving of soil biological activities.

Table6.Response of potato and wheat yield parameters for dry bio slurry and equivalence nitrogen as a main and residual effect

Treatments	**Main effect of DBS on Potato**		**Residual effect DBS on Wheat**		
	NTPP	TYD t ha^{-1}	GY (t ha^{-1})	BY (tha^{-1})	1000 SW(g)
Control (0,0)	4.2^{e}	8.6^{d}	1.23d	3.11d	30.1
RN (138 N $kgha^{-1}$)	10.9^{a}	26.2^{ab}	3.17c	7.9c	32.4
50% DBS (5.3 tha^{-1})	5.0^{de}	12.0^{cd}	3.33bc	8.37bc	32.8
100% DBS (10.6 tha^{-1})	6.4^{cd}	13.4^{c}	3.85a	9.52ab	33.6
75% DBS (7.95 tha^{-1})	7.3^{bc}	14.9^{c}	3.83a	9.59a	33.9
75% DBS+25% N (7.95 tha^{-1}+34.5 N $kgha^{-1}$)	6.5^{bcd}	23.0^{b}	3.47abc	9.08ab	33.2
50%DBS+50% N (5.3 tha^{-1}+69 N $kgha^{-1}$)	6.4^{cd}	24.6^{ab}	3.47abc	8.53abc	32.1
25% DBS+75% N (2.65 tha^{-1}+103.5 N $kgha^{-1}$)	6.8^{bc}	27.6^{a}	3.28bc	8.43abc	32.6
100% DBS+25% N (10.6 tha^{-1}+34.5 N $kgha^{-1}$)	7.0^{bc}	25.2^{ab}	3.70ab	9.56a	33.0
100% DBS+50% N (10.6 tha^{-1}+69 N $kgha^{-1}$)	8.1^{b}	26.8^{a}	3.59abc	9.06abc	32.4
**LSD**	1.7	3.7	0.5	1.2	NS
**CV%**	25.6	19.4	15.6	14.8	10.1

Means followed by the same letter (s) within the column are not significantly different at ($P \leq 0.05$). DBS= dry bio slurry, N= nitrogen, PH= plant height, NTPP = number of tubers per plant, TYD, = total tuber yield, GY= grain yield, BY= above ground biomass and SW = seed weight.

3.3 Economic Analysis

Net benefits were done by calculated current fertilizer (Urea) cost of 0.27 US$ kg^{-1}, cost of DBS kg^{-1} 0.004 US$, field price of potato was 0.12 US$ kg^{-1}, cost of labor per man day in the area is 1.4 US$. The marginal rate of return 100% was used to determine the acceptability of treatments.

The economic analysis indicated that most of treatments give high net benefit than control (Table 7). Addition of 25% DBS with 75% RN gave 2889.2 US$ net benefit with 4486.3% marginal rate of return. This replied that for every 1 US$ invested for 25% DBS with 75% RN in field, farmers can obtain an additional 44.863 US$ (22). All Un-dominated treatment rates could be acceptable for potato producers in the study area except the dominated one's. Therefore, the most economical rate for producers with low cost and higher benefit was 25% DBS with 75% RN.

Table 7. Partial budget and marginal analysis of potato as affected by the application of dry bio-slurry with nitrogen at Yilemana Densa District.

Treatments (RN +DBS $kgha^{-1}$)	10% Adjusted tuber Yield t ha^{-1}	Total variable Cost US$$ha^{-1}$	Net Benefits US$ ha^{-1}	MRR%
Control	7.74	0	928.8	-
50% DBS	10.8	35.2	1260.8	943.2
75% DBS	13.41	52.8	1556.4	1679.6
100% DBS	12.06	70.4	1376.8	D
50% DBS+50% N	22.14	84.5	2572.3	D
25% DBS+75% N	24.84	91.6	2889.2	4486.3
100% DBS+25% N	22.68	95.1	2626.5	D
RNP	23.58	98.7	2730.9	D
100% DBS + 50% N	24.12	119.7	2774.7	D
75% DBS + 25% N	20.7	217.5	2266.5	D

RNP=percent of recommended nitrogen and phosphorus in kg per hectare, DBS=dry bio-slurry in kilogram per hectare, MRR= marginal rate of return; D= is dominated treatments

4. Conclusion and Recommendation

Yield and yield components of potato and wheat were significantly affected by main and residual effect of dry bio slurry and nitrogen substitution. Both crops productivity were improved by substitution of dry bio-slurry and nitrogen as compared to control. Applied dry bio-slurry with N (nitrogen) had a positive impact on plant height, number of tubers per plant, total tuber yield, grain

yield and above ground biomass. Substitution of nitrogen by Dry bio-slurry rate in most treatments gives higher net benefit as compared to control. Among treatments undominated once gave better net benefit with acceptable marginal rate of return than control. Therefore, small-scale farmers can benefited, if they apply these soil improvement rates depending on their convenience. Related to this application 25% DBS with 75% N was given the highest net benefit with 4486.3% marginal return. So that, use of 25% DBS with 75% RN could be promoted for the production of potato and wheat in the study area. For the future, similar studies should be done across locations and crops, in permanent plots for sustainable crop production and soil health enhancement .

Competing of interest

There is no conflict of interest between Authors

Acknowledgment

The author is grateful to Adet Agricultural research center for its administrative and budget support

Financial disclosure

The research supported by budget by Amhara Agricultural Research Institute (ARARI) and the funder hand no role in data collection and designs and none of the authors had not receive salary from the funder

Reference

1. Brankatschk,G, Finkbeiner, M. Modeling crop rotation in agricultural LCAs - challenges and potential solutions. Agric.Syst.2015;138,6676.https://doi.org/10.1016/j.agsy.2015.05.008.

2. Nemecek, T., Hayer, F., Bonnin, E., Carrou´ee, B., Schneider, A., Vivier, C. Designing eco-efficient crop rotations using life cycle assessment of crop combinations. Eur. J. Agron.2015; 65, 40–51. https://doi.org/10.1016/j.eja.2015.01.005.

3. Birch PRJ, Bryan G, Fenton B, Gilroy EM, Hein I and Jones JT. Crops that feed the world 8: potato: are the trends of increased global production sustainable? Food Secur.2012;4(4):477–508. https://doi.org/10.1007/s12571-012-0220-1.

4. CSA (Central Statistical Agency). Agricultural sample survey 2019/20 (2012 E.C) volume I. Report on area and production of major crops meher season crops for private peasant holdings. 2020;CSA, Addis Ababa.

5. CSA (Central Statistical Agency). Agricultural sample survey: Area and production of major crops. 2021; Addis Ababa, Ethiopia.

6. FAOSTAT. Food and Agriculture Organization corporate statistical database of the United Nations. 2021; Retrieved from http://www.fao.org/faostat/en/#data/QC

7. Efrem B, Hirut K, Getachew B. Durum wheat in Ethiopia: an old crop in an ancient land. 2000;Addis Ababa: Institute of Biodiversity Conservation and Research.

8. Chanie, C., Teshome, T., Temesgen, T., & Berihun, B. Characterization of potato production, marketing, and utilization in North Western Amhara Region, Ethiopia. Journal of Horticulture and Forestry.2017;9(3), 17–25. doi:10.5897/JHF

9. Shankarappa TH. Gurumurthy SB, Patil SV and Lokesh MS. (2012). Influence of phosphorus enriched biogas spent slurry (BSS) on growth and yield of sunflower (Helianthus annuus). Journal of Plant Science.2012; 7: 253-258

10. Khan SA, Malav LC, Kumar S, Malav MK and Gupta N Resource utilization of biogas slurry for better yield and nutritional quality of baby corn. Advances in Environmental and Agricultural Science.2015;382-394.

11.Nitesh Samadhiya, SP Singh, Girish Goyal, Murlidhar J Sadawarti, SK Sharma and LK Tripathi. Evaluation of organic and inorganic sources of nutrients in maize (Zea mays L.): Potato (Solanum tuberosum L.) cropping system. The Pharma Innovation Journal.2021;10(7):1360-1365.

12. S. Bilkis, M.R. Islam*, M. Jahiruddin, M. M. Rahman and T.S. Hoque. Residual effects of different manures and fertilizers applied to preceding potato crop on succeeding mung bean (vigna radiate l.) crop in potato-mung bean-rice cropping pattern. SAARC J. Agri.2018;16(2):167-179.

13. Yalemtsehay D and Fisseha I. Comparative study on the effect of applying biogas slurry and inorganic fertilizer on soil properties, growth and yield of White Cabbage (Brassica oleracea var. capitata f. alba). Journal of Biology, Agriculture and Healthcare. 2016;6(19).

14. Karki KB. Estimation of plant nutrient loss from biogas slurry. Biogas Support Programme.1997;

15. Errebhi, M., Rosen, C.J., Gupta, S.C. and Birong, D.E. 1998. Potato Yield Response and Nitrate Leaching as Influenced by Nitrogen Management. Agron. J. 1998;90:10–15.

16. Alva, A. 2004. Potato nitrogen management. J. Veg. Crop Prod.2004; 10: 97-130.

17. White, P.J., Wheatley, R.E., Hammond, J.P. and Zhang, K. Minerals, soils and roots. In: Vreugdenhil D (ed) Potato biology and biotechnology, advances and perspectives. Elsevier, Amsterdam.2007;pp: 739-752.

18. Zelalem, A., Tekalign, T. and Nigussie, D. Response of potato (Solanum tuberosum L.) to different rates of nitrogen and phosphorus fertilization on vertisols at DebreBerhan, in the central highlands of Ethiopia. African journal of plant science.2009;3: 16-24.

19. Rhaman M.S., Kibria M.G., Hossain M., and Hoque M.A. Effects of organic manure and bio-slurries with chemical fertilizers on growth and yield of rice (cv. BRRI dhan28). Int. J. Expt. Agric.2016;6 (3): 36-42

20. Eyasu E . Soils of the Ethiopian highlands: Geomorphology and Properties. CASCAPE Project, ALTERA, Wageningen University and Research Centre (Wageningen UR), the Netherlands.2016; p. 385

21. Sahlemedihn S and Taye B. Procedures for soil and plant analysis.2000; Addis Ababa, Ethiopia.

22. CIMMYT. From Agronomic Data to Farmer’s Recommendation: an Economic Training Manual. 1988;Revised Edition. Mexico, D.F.

23. Gomez K.A and A.A. Gomez. Statistical procedures for agricultural research, 2nd edition. John Wiley and Sons, New York. International Congress Series.1984; 1291: 42-52.

24. Tekalign T. Soil, plant, water, fertilizer, animal manure and compost analysis. Working Document No. 13. 1991;International Livestock Research Center for Africa, Addis Ababa.

25. Olsen S.R, Cole C.V, Watanabe F.S and Dean L.A. Estimation of available phosphorus in soils by extraction with sodium carbonate. USDA Circular.1954;939: 1-19.

26. Landon J.R. Booker tropical soil manual: a handbook for soil survey and agricultural land evaluation in the tropics and subtropics. New York;1991.

27. Workineh E,Yihenew G, Eyasu E and , Matebe D.. Integrated fertilizer application improves soil properties and maize (Zea mays L.) yield on Nitisols in Northwestern Ethiopia. Journal of Helyion.2021;7-e06074

28. Geremew B , Girma A and Birhanu B. Effects of dry bioslurry and chemical fertilizers on tomato growth performance, fruit yield, and soil properties under irrigated conditions in Southern Ethiopia.African Journal of Research.2019; 14(33), pp. 1685-1692.

29. Tsegaye T, Tewodros A and Hussien M.2018. Combined Application of Bioslurry and Inorganic Fertilizers on Quality Traits of Cabbage and Soil Properties. Asian Journal of Biological Sciences.2018;11 (1): 24-32

30. Zelalem A, Tarekegn & Hussien M.2020. Effect of liquid bio-slurry and nitrogen rates on soil physico-chemical properties and quality of green bean (Phaseolus vulgaris L.) at Hawassa Southern Ethiopia.Journal of plant interaction.2020;15 (1):207–212.

31. Sandeep S & Salwinder S.D. Effect of Sewage Sludge and Rice Straw Compost on Yield, Micronutrient Availability and Soil Quality under Rice–Wheat System. Journal Communications In Soil Science and Plant Analysis.2019; DOI:10.1080/00103624.2019.1648489.

32. Shahzad, K., A. Khan, J.U. Smith, M. Saeed, S.A. Khan and S.M. Khan. Residual effects of different tillage systems, bio slurry and poultry manure on soil properties and subsequent wheat productivity under humid subtropical conditions of Pakistan. International journal Biosci.2015; 6(11): 99-108.

33. Tamado T, Mitiku W. 2017. Effect of combined application of organic and mineral nitrogen and phosphorus fertilizer on soil physico-chemical properties and grain yield of food Barley (Hordeum vulgare L.) in Kaffa Zone, South-western Ethiopia. MEJS.2017; 9(2):242–261.

34. Melkamu A, Minwyelet J, Tadele Y & Masho A.2020. Integrated application of compound NPS fertilizer and farmyard manure for economical production

35. Moniruzzaman M, Halim G.M.A and Firoz Z A. Performances of the French bean as influenced by plant density and nitrogen application. Bangladesh. JAR.2009;34(1): 105-111.

36. Muhammad U.C, Muhammad U.H, Imran K. Effect of different nitrogen and phosphorus fertilizer levels in combination with nitrogen and phosphorus solubilizing inoculants on the growth and yield of mung bean. PJLSS. 207;15(1): 31-36.

37. De La, I., Guillen, I. A., & Del Moral, L. F. G. Yield development in potatoes as influenced by cultivar and the timing and level of nitrogen fertilization. American Potato Journal,1994; 71, 165–173. Doi: 10.1007/ BF02849051

38. Balasubramanian A, P. Stalin, M.Saravanaperumal and S.R.Vinoth kumar. Residual effect of Integrated Nutrient Management practices on growth and yield of rice fallow blackgram (Vigna mugno L.). Journal of Emerging Technologies and Innovative Research,2016; 3(5): 2349-5162.

39. Bilkis S, M.R. Islam, M. Jahiruddin, M.M. Rahaman. Integrated Use Of Manure And Fertilizers Increases Rice Yield, Nutrient Uptake And Soil Fertility In The Boro-Fallow-T.Aman Rice Cropping Pattern. SAARC J. Agri,2017; 15(2): 147-161.

40. Pandey B.P, N. Khatri, M. Yadav, K. R. Pant, R. P. Poudel, and A. H. Khan. Effect of Digestate /Biogas Slurry In Wheat Under Rice – Wheat Cropping System. Journal of Agriculture and Forestry University.2020;4: 67-75.

41. Tsegaye G,Birhanu B, S B & Berga L.2020. Integrated Application of Organic and Blended Mineral Fertilizers Improves Potato Productivity and Income for Smallholder Farmers in Acidic Soils. Journal of fertilizer and pesticides.2020;8(3),2471-2728.

42. Haque M.A, M. Jahiruddin, M. S. Islam, M. M. Rahman, M. A. Saleque. Effect of Bio slurry on the Yield of Wheat and Rice in the Wheat– Rice Cropping System. Journal of Agric Res.2018. 7(4):432–442.

43.Shahid, M., A. K. Shukla, P. Bhattacharyya, R. Tripathi, S. Mohanty, A. Kumar, B. Lal, P. Gautam, R. Raja, B. B. Panda., et al. Micronutrients (Fe, Mn, Zn and Cu) balance under long-term application of fertilizer and manure in a tropical rice–rice system. Journal of Soils and Sediments.2016; 16 (3):737–47. doi: 10.1007/s11368-015- 1272-6.

44. Mercy K, Esther M, James N, Kevin K, Stephen Mailu,Peterson Njeru and Rewe Thomas.2022. Integrated effect of liquid bio slurry and inorganic fertilizer on selected soil chemical properties, maize (Zea mays) growth, yield and grain quality. African Journal of Crop Science, 2022;10 (5): 001-016.

45.Liu, E., Yan, C., Mei, X., He, W., Bing, S.H., Ding, L. Long-term effect of chemical fertilizer, straw, and manure on soil chemical and biological properties in northwest China. Geoderma .2010;158, 173–180

46. Efthimiadou A, Bilalis D, Karkanis A and Froud W.B. Combined Organic and Inorganic Fertilization Enhances Soil Quality and Increased Yield, Photosynthesis and Sustainability of Sweet Maize Crop. AJCS.2010; 4(9):722-729.

47. Tadele Amare, Zerfu Bazie, Erkihun Alemu, Beamlaku Alemayehu, Abere Tenagne, Bitewlgn Kerebh, Yasin Taye, Abrham Awoke, Tesfaye Feyisa, Selamyihun Kidanu. Yield of potato (Solanum tuberosum L.) increased by more than two-folds through nitrogen and phosphorus fertilizers in the highlands of North-Western Ethiopia. Journal of Heliyon.2022;8(11111).

Figure

Click here to access/download;Figure;Figure1; Study Area.jpg

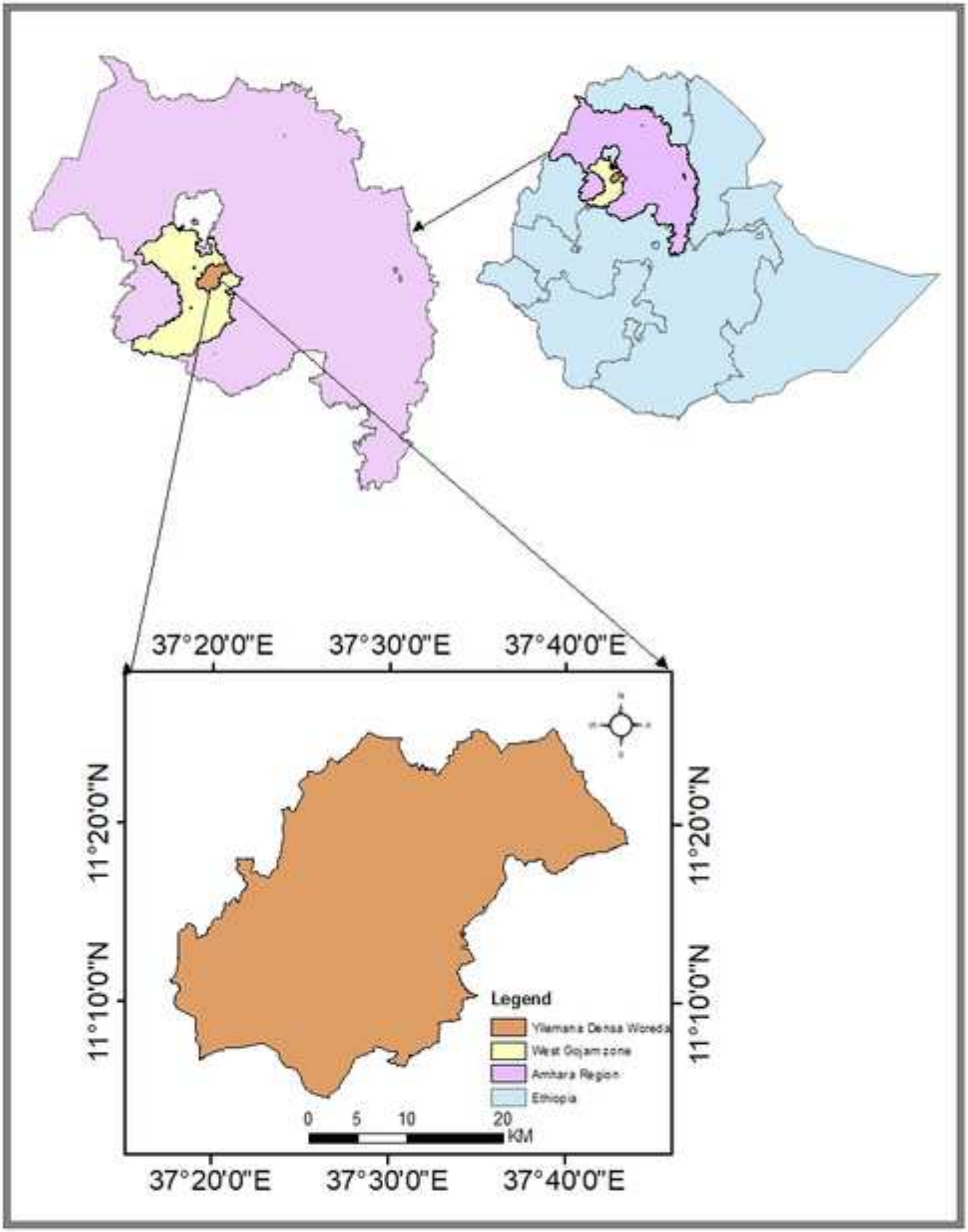

Effects of dry-bio-slurry with nitrogen fertilizer on yield of potato and wheat under short term crop rotation

Zelalem Addis*[1], Tadele Amare[1], Bitewlgn Kerebih[1], Antenh Abewa[1], Tesfaye Feysa[2], Abrham Awoke[1] and Abere Tenagne[1]

1 Adet Agricultural Research Center P.O.BOX 08, Bahir dar Ethiopia

2 Amhara Agricultural Research Institute

*Corresponding Author zelalemaddis660@gmail.com

ABSTRACT

Effective cropping system with nutrient balancing is the key solution in farming practices by improving of nutrient use efficiency, reduce need for inorganic fertilizers and impact of pests and diseases. The experiment was conducted to fill the gap of using balanced nutrient in crop rotation system for the improvement of both soil properties and crop yields. That was done by substitution balancing ratio of dry bio-slurry and nitrogen fertilizers for the enhancement of soil properties and potato-wheat yields. The experiment was contain ten levels of treatments Control (0.0), RN (recommended nitrogen), 50% DBS (dry-bio slurry), 100% DBS (dry-bio slurry), 75% DBS (dry-bio slurry), 75% DBS (dry-bio slurry)+25% RN (recommended nitrogen), 50% DBS (dry-bio slurry)+50% RN (recommended nitrogen), 25% DBS (dry-bio slurry)+75% RN (recommended nitrogen), 100% DBS (dry-bio slurry) +25% RN (recommended nitrogen) and 100% DBS (dry-bio slurry)+50% RN (recommended nitrogen) that were laid out in RCBD design with three replications for three years. Data on soil Physico-chemical properties and yield components of potato and wheat were collected and subjected to ANOVA using SAS software 9.4. The results were indicated that; an application of dry bio-slurry with nitrogen fertilizer was significantly affected the yield and yield components of both potato and wheat in their rotation system. Soil

properties also, significantly changed by the residual effect of dry bio-slurry. Applying 25% DBS with 75 % RN was given the highest tuber yield of potato (27.6 tha-1) as compared to control. Similarly, an application of 100% and 75% sole dry bio-slurry scored the highest grain yield (3.85tha^{-1}) and above-ground biomass (9.59tha^{-1}) of wheat respectively. The combination of 25% DBS with 75% RN also, recorded the highest net benefit (2889.2 US$) with acceptable marginal return (4486.2%). So that, to attain the highest net benefit, application of 25% DBS with 75% RN could be promoted for yield improvement in the study area and similar agroecology.

Key Words: dry bio-slurry, nitrogen, soil properties, potato, wheat

1. Introduction

A well-planned cropping system can enhance nutrient use efficiency, reduce the need for inorganic fertilizers and lessen the impact of pests and diseases (1&2). Potato and wheat production under a crop rotation system is commonly practiced by farmers in the Amhara region. The system comprises a cradle-to-farm analysis for 3-years rotation cycle. It allows mitigating the impact of certain pests and diseases, which significantly affects the yield of the main crop potato. In addition to this, it enables to take full advantage of residual fertility that comes from potato leaves entire decomposition in the soil. This rotation system was chosen by many producers and it is considered as the most efficient system from an economic, quality and soil health point of view. Potato (*Solanum tuberosum* L.) is the third most important food crop after wheat and rice in the world (3). In Ethiopia, the area coverage under potato cultivation reaches about 73,677.64 ha and its production was estimated around.1,044,436.359 tons (4). The productivity of potato in Ethiopia is reached about 13.9 t ha^{-1} (5); which is relatively low compared to other African countries (6). Bread wheat (*Triticum aestivum* L.) is also is one of the major cereal crops grown in the highlands

of Ethiopia, and it makes the country is regarded as the largest wheat producer in Sub-Saharan Africa (7).

Out of the total grain crop area coverage, wheat ranked 4th after tef (*Eragrostis tef*), maize (*Zea mays*) and sorghum (*Sorghum bicolor*) while, it is the third in total production after maize and tef (5). Despite the long history of wheat cultivation and its importance to Ethiopian agriculture, its average yield also still very low, not exceeding 2.4 t ha^{-1} (5). Which is below the world's average yield of wheat 3.4 t ha^{-1} (6). This phenomenon is common in the Amhara region. Low level of potato-wheat productivity is mainly due to soil fertility degradation, improper fertilization, poor pest management practices, use of the low-quality seed and soil nutrient depletion (8). Enhancing soil fertility is the first precondition for a practical crop production system; that can be achieved through the application of organic manure (like bio-slurry) and inorganic fertilizers integration for sustainable crop productivity (9 & 10). Different studies also indicated that judicious application of both organic and inorganic fertilizers is a key solution for crop productivity and soil fertility enhancement. The study conducted by (11) revealed that integrated application of both organic and inorganic source fertilizers significantly improved yield and soil fertility status under maize - potato cropping system .Another study which done by (12) indicated that the supplement of tricho compost with chemical fertilizer and vermicompost with chemical fertilizer significantly affected the yield of potato,mungbean and T.aman rice by scoring of 6.3-33.7%,8.3-33.8% and 2.9-18.3% respectively over sole application treatment .Similarly the study conducted by (42) also observed that poultry manure bio slurry, poultry manure, cow dung bio slurry, and cow dung gave 11.7, 8.9, 5.4, and 3.1% respective increase in total system productivity over sole chemical fertilizer (46% N, as urea, 20% P as triple superphosphate, 50% K as muriate of potash and 18% S as gypsum).Bio-slurry that obtained after extraction of the energy content of animal manure is an

excellent fertilizer, rich in major nutrients (nitrogen, phosphorous and potassium) and organic matter which is important for soil fertility and yield of crops (13). It also improves the physical and biological quality of soil beyond its role in the providing of plant nutrients. In addition, an application of bio-slurry can help in the reduction of dependence on mineral fertilizers (14). Because of nitrogen is the most limiting essential nutrient for growth and development of crops, both potato and wheat are highly responsive to N fertilization (15). Furthermore; supplying of nitrogen fertilizer plays an important role in the balance between vegetative and reproductive growth of crops (16&17). Various studies showed that N fertilizer applications can increase the dry matter protein content of wheat and potato tubers (18). Moreover, most of the time the available nitrogen concentration in most organic source fertilizers including bio-slurry is high as compared to other major nutrients (13) Due to this the application of dry bio-slurry with nitrogen fertilizer through nitrogen equivalence balancing ratio is very important. Beside this, neither organic manure nor chemical fertilizer alone can meet the nutrient demand of a given crop and also their own negative impact in terms of sustainable crop and soil productivity in different cropping system (19). For instance, continuous addition of chemical fertilizers can cause soil quality deterioration such as increasing of soil acidity, loss of organic matter and depletion of nutrients that are not supplied in the fertilizer formulation (45). On the other hand, organic fertilizers are required in large quantities and their nutrients released slowly that make less efficient on soil properties and crop yields within short period of time as compared to inorganic fertilizers (46). Because of these reasons, the study was conducted to determine the main and residual effects of dry-bio slurry and nitrogen fertilizers on soil and yield of potato and wheat under crop rotation system at Yilemana Densa District in North Western Amhara Region Ethiopia

2. Material and methods

2.1 Description of Study Area

The experiment was conducted at Yilemana Densa district on Farmer's field across three sites (Y1S1, Y2S1and Y2S2) for three consecutive years (2019-2022) as potato and wheat rotation (potato-wheat cropping system) at Debremewi Keble West Gojam Zone North Western Amhara Region of Ethiopia. Geographically the area lies at 11^0 21' 22" N and 37^0 25' 43" E (Figure 1) with a mean altitude of 2304 m above sea level. It receives a mean annual rainfall of 1421 mm with mean minimum and maximum temperatures of 12.29 and 27.56 °C, respectively. The landforms of the area are characterized by undulating to rolling plateaus, scattered moderate hills, dissected side slopes, and river gorges (20). Based on the District Bureau of Agriculture, the major land use comprises cultivated land (57%), forest and bushes (2%), grazing land (33%), and others (8%). Major crops, grown in the study area are Maize, Tef, wheat, Barley, Potato, and Field pea. Soil types in the area are Nitisols (45%) Vertisols (30%) and Luvisols (25%). This on-farm experiment was conducted on Luvic Nitisols which is the most dominant soil in the study area

2.2 Experimental procedure

The experiment was contain ten treatments which includes control (without N and DBS), recommended N (138 N) ,dry-bio slurry 50% equivalence (5.3 tha^{-1}), dry-bio slurry 100% equivalence (10.6 tha^{-1}), dry-bio slurry 75% equivalence (7.95 tha^{-1}), dry-bio slurry 75% equivalence (7.95 tha^{-1}) + 25% recommended N (34.5 N), dry-bio slurry 50% equivalence (5.3 tha^{-1}) + 50% recommended N (69 N), dry-bio slurry 25% equivalence (2.65 tha^{-1}) + 75% recommended N (103.5 N), dry-bio slurry 100% equivalence (10.6 tha^{-1})+25% recommended N (34.5 N) and dry-bio slurry 100% equivalence (10.6 tha^{-1}) +50% recommended N (69 N) that were laid out in Randomized Complete Block Design (RCBD) with three replications. The rates of dry-bio slurry were adjusted based on potato nitrogen recommended rate 138 $kgha^{-1}$ N (47)

equivalency corresponding to its N content. Urea was used as a source of synthetic 138N whereas, phosphorous at the rate of 69 kg ha^{-1} P_2O_5 was applied in the form of TSP to all plots uniformly. The experiment was carried out under rain-fed conditions GUDENIE and TAY varieties were used as test crops for potato and wheat respectively in each rainy season one after the other. Total area of each plot was 4.5 m x 3 m (13.5 m^2) having 1 m space between plots and 1.5 m between blocks. Potato was spaced by 0.3 m between plants & 0.75 m between rows and the data were collected from the middle four rows. Whereas; Wheat was planted as a rotated crop in 0.2 m and harvested from middle rows by avoiding four rows as a border.

2.2.1 Dry bio-slurry

It was collected from farmers biogas plants and stored under well-protected shade for better drying and avoiding nutrient loss especially nitrogen. After drying, we make it fine and break into small pieces for nice application and soil reaction. After this; during planting period of time dry-bio slurry was incorporated into the soil. Representative composite sample was taken from the whole collected dried pit of slurry for analysis of hydrogen concentration (pH), organic carbon (OC%), cation exchange capacity (CEC), total nitrogen (TN%) and available phosphorus (P) by following laboratory manual procedures (21).

Table 1. Physico-chemical analysis of dry-bio slurry before incorporation in year1and 2 and its average

Dry bio-slurry	Concentration
**Year 1**	
Dry matter%	11.2
OC%	27.8
TN%	1.2
Av P (mg kg^{-1})	112.1
pH (H_2O; 1:2.5)	7.9
C:N ratio	22.9
CEC (cmol kg^{-1})	59.8
**Year 2**	
Dry matter%	11.8

OC%	16.3
TN%	1.4
Av P (mg kg^{-1})	91.6
pH (H_2O; 1:2.5)	7.7
C:N ratio	11.6
CEC (cmol kg^{-1})	67.4
**Average result of Year1andYear 2**	
Dry matter%	11.5
OC%	22.1
TN%	1.3
Av P (mg kg^{-1})	101.9
pH (H_2O; 1:2.5)	7.8
C: N ratio	17.3
CEC (cmol kg^{-1})	63.6

OC%=organic carbon percent, TN%=total nitrogen percent, C: N=carbon to nitrogen ratio, CEC = cation exchange capacity, AvP=available phosphorus, and pH= Power of hydrogen concentration.

2.2.2 Experimental crops

Gudenie variety is used as attest crop for potato and it was planted as main crop or precursor crop of wheat. TAY variety of wheat also planted in the next cropping season after potato which was used for the determination of dry bio slurry residual effect in potato-wheat rotation system. TSP (P_2O_5) was applied during the planting period of time as basal whereas inorganic N from urea was applied in three splits for potato; one-third third at planting, one-third 30 days after planting, and the remaining one-third at the beginning of the flowering. While for wheat recommended NP was applied in all plots except control which only received P_2O_5 to see the residual effect of dry bio-slurry in the next crop and nitrogen was supplied, in two splits.

2.3 Data Collection, Preparation, and Analysis

2.3.1 **Soil sampling and analysis of before planting**

Before planting, representative soil samples were collected from 0-20 cm depth in the sites via random sampling method from 10 spots using an auger. All samples were mixed together to form one composite sample. The sample was grounded by using a mortar and passed through a 2 mm sieve for analysis of soil texture, CEC, pH, and available P whereas ;a 0.5 mm sieve was used for

determining the organic carbon (OC) and total N.While, bulk density was determined by the core sampling method.

2.3.2 Soil sampling and analysis after harvesting of potato and wheat

The major chemical properties of soil such as OC, pH, CEC, total N and available P were analyzed following the compiled laboratory manual of (21). Soil pH was measured in water with the ratio of 1:2.5 using glass electrode pH meter. The soil OC content was determined following the wet digestion method as outlined by Walkley and Black which involves the digestion of soil OC with potassium dichromate ($K_2Cr_2O_7$) in a sulphuric acid solution. AvP was determined by Olsen extracting method. The total N content in the soil samples was determined following the Kjeldahl method. CEC also, determined by extracting the soil samples with ammonium acetate (1NNH$_4$OAc) followed by repeated washing with ethanol (96%) to remove the excess ammonium ions in the soil solution. Percolating the $NH4^+$ saturated soil with sodium chloride would displace the ammonium ions adsorbed in the soil and the ammonium liberated from the distillation was titrated using 0.1N NaOH.

2.3.3 Crop data collection

Plant height for both potato and wheat was measured at the maturity stage by taking five randomly selected plants from ground to the top apex and averaging for a single reading. Similarly, spike length of wheat was measured at the maturity stage by taking five randomly selected plants from staring point of spike grain to the end of spike grain and averaging for a single reading. Number of plants per hill (stem number per hill) was recorded by counting the stems that were directly sourced from original seed tuber of five hills of potato at 70% maturity stage.

Number of tubers per plant also taken at the maturity stage by counting potato tubers from five randomly selected plants in the middle four rows of experimental plots (7.2 m^2) and averaged for

single reading. While thousand seed weight for wheat was taken from each treatment harvestable plot (11.7 m^2) by counting of 1000 seeds at maturity stage after threshing and make them weighting under sensitive balance for weight reading. Total tuber yield of potato was measured by using balancing balance during harvesting period of time from the middle four rows (7.2 m^2) for total weight reading. Grain yield, and above-ground biomass of wheat was measured by using balancing balance at maturity stage from the net harvestable plot area (11.7 m^2).

2.4 Economic Analysis

The economic analysis was performed to make rational choice among the applied variables in the production of potato. The partial budget and marginal rate of return (MRR) were used for evaluating the change in farming methods that affect partially rather than the whole farm practice and also concerned with planning tool to estimate the profit change within a farm (22). This was computed by adjusting yield downward by a 10% and multiplying it with the local field price (6 Ethiopian Birr or 0.12 US$ per kg of potato). Dominance analysis was done by listing of treatments in an increasing order of cost and that has net benefit less than or equal to treatments with the lower costs that vary is dominated (22).

2.5 Statistical Analysis

All collected data's was subjected to analysis of variance through GLM procedure by using SAS software program version 9.4 (SAS Institute, 2014). List significant difference (LSD) at 0.05 probability level was employed to differentiate treatments means t (23).

3. Results and discussion

3.1 Soil chemical properties before planting and after harvesting of potato

The results of soil chemical properties before and after harvest from each experimental sites were displayed in Table 2, 3 & 4. Before-planting the soil was acidic in reaction with a pH (H_2O 1:2.5)

value of 5.1 Y1S1 (year one site one) and 5.2 Y2S1 (year two site one), even if, it was within the range of soil pH for potato production (24) (Table 2). The total N, available P, OC, C: N ratio and CEC of the soil Y1S1 and Y2S1 before planting were 0.16&0.16%, 6.9&6.3 mg kg^{-1}, 1.4 &1.5%, 11.1&9.3 and 33.9&30.3 cmol (+) kg^{-1}, respectively (Table 2). The total N content of the soil was within the range of medium according to (24) who was classified the range of total N < 0.1, 0.1-0.15, 0.15-0.25 and > 0.25% as very low, low, medium and high, respectively. (25) also, classified the range of soil available P content was like this < 5 as very low, 5 -15 as low, 15 -25 as medium and > 25 mg kg^{-1} as high. Hence the available P of the soil before planting lies under the low range. According to (26) the soil OC content ranges was rated 1-2, 2-4, and 4-6% as low, medium and high, respectively. Similarly; cation exchange capacity (CEC) was also rated with the ranges of 5-15, 15-25 and 25-40 cmol kg^{-1} as low, medium and high, respectively (26).

Based on these ratings OC (1.4, 1.5 &2.0%) and CEC (33.9, 30.3&30.9 cmol kg^{-1}) before planting of the experimental fields (Y1S1, Y2S1 and Y2S2) were in the low and high ranges, respectively. Generally, the nutrient contents of the study site Y2S1 is not good in terms of availability of major plant nutrients beside its nice CEC. On the other hand, on Y2S2 pre planting values of pH, TN, AvP, OC, C: N and CEC were 5.5, 0.17%, 6.8 ppm, 2.4%, 14.1 and 33.9 respectively. Based on (24) rating the pH value was under moderate while total nitrogen was medium. Similarly, according to (25) available P was under low while OC and CEC were medium and high respectively (26). Y2S2 rather than the two sites (Y1S1 and Y2S1) had a good soil fertility status based on its soil chemical properties. However, the acidity of soil Y1S1andY2S2 may be cause the sorption of available P. Thus, application of OM like bio-slurry is very essential in order to neutralize soil solution for the availability of nutrients. On the other hands, after harvest all soil

chemical properties except soil pH (on Y2S2) were not affected by the application of dry bio-slurry with N equivalence rates (Table3).

The non-significant effects of the applied treatments on the rest soil properties might be the slow release of nutrients from dry bio-slurry that was applied during the experimentation period to soil solution. However, applied dry bio-slurry and N rates had a significant ($P < 0.05$) effect on soil pH at site two in 2020-21 as compared to the control (Table 2). Numerically the highest soil pH was obtained by the application of 100% dry bio-slurry and 100% dry bio-slurry +25% RN as compared to the control (Table 3). The increment of soil pH in treated plots may be related due to the releasing of basic cations from dry bio-slurry into soil solution that makes for the substitution of acid cations. These results were in agreed with the investigation of (27) they reported that the combined application of compost with inorganic NPSB on maize increased the soil pH content after harvest as compared to control.

3.2 Residual effect of dry bio-slurry on soil chemical properties after harvesting of wheat

The residual effect of dry bio-slurry on selected soil chemical properties was significantly explained at ($p < 0.05$) across sites and years in table 4. Related to this an addition of 100% ($10.6tha^{-1}$) dry bio-slurry with 50% RN (69 kg N) was given the highest TN% (0.205 and 0.223) on Y1S1 and Y2S2 respectively than control. This might be due to the gradual releasing of dry bios-slurry nitrogen into soil solution beyond its chelating capacity. The finding lined with (28) they found that an application of dry bio-slurry with inorganic fertilizers gives the maximum value of total nitrogen than control. Similarly, the study conducted by (29) also indicated that an application of $70cm^3$ scored the highest (1.36%) total nitrogen as compared to control which was given 0.07%.

On the other hand, OC, C: N and CEC were significantly affected at Y2S1 (Table 4),While; in Y2S2 only CEC is significant at ($p < 0.05$) Table 4.Based on this; numerically the highest value of CEC (34.6), C: N (12.82) & OC% (2.20) was observed by application 50% dry bio slurry (DBS) and 100% dry bio slurry (DBS) with 25% RN as compared to control on Y2S1.On Y2S2 the maximum value of CEC (cmolkg^{-1}) 36.06 was obtained in plots that receive 100% DBS with 25%N than control. This might be the positive effect of applied DBS on the improvement of organic matter and soil holding capacity of positive cations on its exchangeable site. The finding agreed with (30) who revealed that an application of 41.3 m^{-3} liquid bio-slurry with 20.5 kgha^{-1} N significantly increased soil organic carbon than untreated plots. The study conducted by (31) also indicated that an application of 10 tha^{-1} compost with 50% N kgha^{-1} significantly increased OC% by scoring the maximum value of 0.67 than the control treatment. Organic amendments significantly enhanced SOC and they make a considerable effect on soil microbes abundance and nutrient availability and uptake this may alter the C: N ratio of the soil. However, the addition of external organic matter with a low C: N ratio may induce the mineralization of OM. This makes nitrogen trapped by organic matter; a phenomenon known as the priming effect (32). The application of the 50% DBS and 100% DBS with 25% N gives the highest CEC (34.67 & 36.06) at Y2S1 and Y2S2 respectively as compared to the control (Table 4). Such increment in CEC might be due to the application of DBS on soil, that makes the colloidal site of the soil negatively charged for storing of basic cations. The finding in agreed with (33) who reported that the use of organic FYM and inorganic fertilizers significantly increased CEC over the control.

Table 2. Initial soil properties of the experimental sites

**Texture SCL**	**Years**	**Initial soil properties across sites and years**						
		BD	pH	TN%	Av P (ppm)	CEC (cmol kg^{-1})	C: N	OC%
Sandy= 56%	2019-20 (Y1S1	1.22	5.1	0.16	6.9	33.9	8.8	1.4
Clay = 32%	2020-21 (Y2S1)	1.33	5.2	0.16	6.3	30.3	9.3	1.5
Loam =12%	2020-21 (Y2S2)	1.26	5.5	0.17	6.8	33.9	11.8	2.0

NB: SCL= sandy clay loam; BD= bulk density**;** Y1S1= year one site one, Y2S1= year two site one and Y2S2 = year two site two.

Table 3. Main effects of dry bio slurry with nitrogen on soil chemical properties after harvest of potato

			**2019-20 (Y1S1)**			
**Treatment**	pH	TN%	Av P (ppm)	CEC (cmol kg^{-1})	C: N	OC%
Control (0,0)	5.13	0.12	9.3	29.1	11.6	1.4
RN (138 N kg-ha^{-1})	5.11	0.12	7.8	29.9	11.61	1.3
50% DBS (5.3 t-ha^{-1})	5.19	0.13	8.8	29.6	11.59	1.4
100% DBS (10.6 t-ha^{-1})	5.18	0.13	9.6	29.7	11.58	1.5
75% DBS (7.95 t-ha^{-1})	5.19	0.13	10.2	29.1	11.62	1.5
75% DBS+25% N (7.95 tha^{-1}+34.5 N kg-ha^{-1})	5.22	0.16	10.1	28.3	11.57	1.9
50% DBS+50% N (5.3 tha^{-1}+69 N kg-ha^{-1})	5.10	0.15	9.1	28.7	11.59	1.7
25% DBS+75% N (2.65 tha^{-1}+103.5 N kgha^{-1})	5.18	0.15	7.5	29.2	11.61	1.7
100% DBS+25% N (10.6 tha^{-1}+34.5 N kgha^{-1})	5.12	0.13	12.3	28.3	11.62	1.5
100% DBS+50% N (10.6 tha^{-1}+69 N kgha^{-1})	5.15	0.13	11.2	27.6	11.60	1.5
**LSD**	**NS**	**NS**	**NS**	**NS**	**NS**	**NS**
**CV%**	**1.2**	**14.3**	**22.43**	**3.3**	**0.21**	**14.19**
			**2020-21 (Y2S1)**			
**Treatment**	pH	TN%	Av P (ppm)	CEC (cmol kg^{-1})	C: N	OC%

Control (0,0)	5.2	0.15	4.4	27.3	11.59	1.7
RN (138 N kgha^{-1})	5.3	0.16	4.3	27.7	11.59	1.8
50% DBS (5.3 tha^{-1})	5.3	0.14	6.5	30.8	11.61	1.7
100% DBS (10.6 tha^{-1})	5.4	0.15	6.2	27.4	11.60	1.8
75% DBS (7.95 tha^{-1})	5.4	0.15	5.8	28.6	11.58	1.7
75% DBS+25% N (7.95 tha^{-1}+34.5 N kgha^{-1})	5.3	0.14	5.4	24.4	11.63	1.7
50% DBS+50% N (5.3 tha^{-1}+69 N kgha^{-1})	5.3	0.14	4.5	26.7	11.61	1.6
25% DBS+75% N (2.65 tha^{-1}+103.5 N kgha^{-1})	5.2	0.14	4.6	27.5	11.59	1.7
100% DBS+25% N (10.6 tha^{-1}+34.5 N kgha^{-1})	5.4	0.17	4.4	28.4	11.59	1.9
100% DBS+50%N (10.6 tha^{-1}+69 N kgha^{-1})	5.4	0.16	4.4	28.5	11.61	1.9
**LSD**	**NS**	**NS**	**NS**	**NS**	**NS**	**NS**
**CV%**	**2.12**	**13.5**	**21.6**	**9.0**	**0.17**	**13.5**

2020-21 (Y2S2)

**Treatment**	pH	TN%	Av P (ppm)	CEC (cmol kg^{-1})	C: N	OC%
Control (0,0)	5.3[cd]	0.17	12.2	30.6	12.10	2.1
RN (138 N kgha^{-1})	5.4b[cd]	0.17	7.1	31.9	11.60	1.9
50% DBS (5.3 tha^{-1})	5.5[abc]	0.18	11.5	30.4	11.60	2.0
100% DBS (10.6 tha^{-1})	5.6[ab]	0.18	9.5	32.1	12.62	2.3
75% DBS (7.95 tha^{-1})	5.6[ab]	0.17	13.2	32.1	12.00	2.0
75% DBS+25% N (7.95 tha^{-1}+34.5 N kgha^{-1})	5.5[abc]	0.18	13.3	33.5	11.60	2.1
50% DBS+50% N (5.3 tha^{-1}+69 N kgha^{-1})	5.5[abcd]	0.17	13.1	32.6	12.01	2.1
25% DBS+75% N (2.65 tha^{-1}+103.5 N kgha^{-1})	5.3[d]	0.16	9.5	30.9	11.60	1.9
100% DBS+25% N (10.6 tha^{-1}+34.5 N kgha^{-1})	5.6[a]	0.18	11.6	31.8	11.80	2.1
100% DBS+50% N (10.6 tha^{-1}+69 N kgha^{-1})	5.4[bcd]	0.17	15.3	33.4	11.60	2.1
**LSD**	**2.3**	**NS**	**NS**	**NS**	**NS**	**NS**
**CV%**	**0.22**	**9.9**	**29.1**	**6.1**	**5.9**	**13.8**

Formatted Table

Means followed by the same letter (s) within the column are not significantly different at ($P \leq 0.05$). control = is no nitrogen and dry bio slurry, RNP=percent of recommended nitrogen and phosphorus, DBS =dry bio-slurry, pH= power of hydrogen concentration, TN% = total nitrogen percent, AvP = available phosphorus, OC% = organic carbon percent, C: N ratio = carbon to nitrogen ratio, CEC = cation exchange capacity, OM% = organic matter percent, Y1S1= year one site one, Y2S1= year two site one and Y2S2 = year two site two.

Table 4. The residual effect of dry bio-slurry on soil chemical properties after wheat harvesting in the potato-wheat cropping system

Treatment	pH	TN%	Av P (ppm)	CEC (cmol kg^{-1})	C: N	OC%
**2020-21 (Y1S1)**						
Control (0,0)	5.40	0.174bcd	8.97	21.39	9.68	1.69
RN (138 N kgha^{-1})	5.40	0.196a	10.12	21.69	10.01	1.95
50% DBS (5.3 tha^{-1})	5.40	0.197a	9.25	26.13	9.64	1.88
100% DBS (10.6 tha^{-1})	5.38	0.168d	11.04	23.51	11.81	1.98
75% DBS (7.95 tha^{-1})	5.40	0.192ab	9.34	25.60	9.42	1.82
75% DBS+25% N (7.95 tha^{-1}+34.5 N kgha^{-1})	5.42	0.172cd	9.07	24.51	11.98	2.06
50% DBS+50% N (5.3 tha^{-1}+69 N kgha^{-1})	5.36	0.188abc	10.06	22.09	9.61	1.80
25% DBS+75% N (2.65 tha^{-1}+103.5 N kgha^{-1})	5.35	0.202a	10.08	25.11	10.14	2.04
100% DBS+25% N (10.6 tha^{-1}+34.5 N kgha^{-1})	5.31	0.194ab	10.46	21.81	11.01	2.13
100% DBS+50% N (10.6 tha^{-1}+69 N kgha^{-1})	5.37	0.205a	10/76	23.78	9.35	1.89
**LSD**	NS	0.020	NS	NS	NS	NS
**CV%**	1.3	6.2	12.7	11.9	14.8	13.3
**2021-22 (Y2S1)**						
Treatment	pH	TN%	Av P (ppm)	CEC (cmol kg^{-1})	C: N	OC%
Control (0,0)	5.61	0.178	10.15	27.70d	7.61d	1.36b
RN (138 N kgha^{-1})	5.53	0.179	10.22	30.52bc	10.31abc	1.85a
50% DBS (5.3 tha^{-1})	5.69	0.180	11.56	34.67a	12.25abc	2.20a
100% DBS (10.6 tha^{-1})	5.67	0.160	9.85	31.77bc	12.42ab	1.98a
75% DBS (7.95 tha^{-1})	5.64	0.194	9.85	31.09bc	9.74cd	1.88a
75% DBS+25% N (7.95 tha^{-1}+34.5 N kgha^{-1})	5.70	0.178	11.76	29.56cd	10.79abc	1.90a
50% DBS+50% N (5.3 tha^{-1}+69 N kgha^{-1})	5.62	0.167	10.69	30.63bc	11.38abc	1.90a
25% DBS+75% N (2.65 tha^{-1}+103.5 N kgha^{-1})	5.54	0.186	9.59	30.37bc	10.00bcd	1.86a
100% DBS+25% N (10.6 tha^{-1}+34.5 N kgha^{-1})	5.58	0.172	9.88	31.99bc	12.82a	2.20a
100% DBS+50% N (10.6 tha^{-1}+69 N kgha^{-1})	5.44	0.185	10.19	32.91ab	11.78abc	2.17a
**LSD**	NS	NS	NS	2.60	2.61	0.41
**CV%**	2.0	6.7	14.4	4.8	14.1	12.6
**2021-22 (Y2S2)**						
Treatment	pH	TN%	Av P (ppm)	CEC (cmol kg^{-1})	C: N	OC%

Control (0,0)	5.51	0.176de	10.03	29.43c	12.03	2.12
RN (138 N kgha^{-1})	5.65	0.190bcd	8.76	30.69bc	12.19	2.29
50% DBS (5.3 tha^{-1})	5.62	0.186bcde	13.49	35.92 a	12.78	2.38
100% DBS (10.6 tha^{-1})	5.62	0.209ab	14.08	33.68ab	11.63	2.43
75% DBS (7.95 tha^{-1})	5.76	0.178cde	11.74	34.47a	13.41	2.39
75% DBS+25% N (7.95 tha^{-1}+34.5 N kgha^{-1})	5.68	0.207ab	12.93	34.81a	12.29	2.54
50% DBS+50%N (5.3 tha^{-1}+69 N kgha^{-1})	5.57	0.202abc	13.08	35.88a	11.47	2.30
25% DBS+75% N (2.65 tha^{-1}+103.5 N kgha^{-1})	5.59	0.164e	11.07	33.27ab	13.52	2.19
100% DBS+25% N (10.6 tha^{-1}+34.5 N kgha^{-1})	5.66	0.223a	11.51	36.06a	10.09	2.24
100% DBS+50% N (10.6 tha^{-1}+69 N kgha^{-1})	5.66	0.207ab	11.21	35.95a	11.75	2.42
**LSD**	NS	0.025	NS	3.78	NS	NS
**CV%**	2.3	7.6	17.8	6.5	10.5	7.3

Means followed by the same letter (s) within the column are not significantly different at ($P \leq 0.05$). RNP=percent of recommended nitrogen and phosphorus, DBS =dry bio-slurry, pH= power of hydrogen concentration, TN% = total nitrogen percent, AvP = available phosphorus, OC% = organic carbon percent, C: N ratio = carbon to nitrogen ratio, CEC = cation exchange capacity, OM% = organic matter percent, Y1S1= year one site one, Y2S1= year two site one and Y2S2 = year two site two.

3.2 Effects of dry bio slurry and nitrogen on yield and yield components of potato and wheat

3.2.1 Plant height and number of Stem per hill of potato

Integration of dry bio slurry with nitrogen (N) was significantly ($P < 0.05$) affects plant height (Table 5). Numerically,the highest values of plant height (52.1 and 48.8 cm) was achieved with addition of recommended nitrogen (RN) and 25% DBS with 75% N recpectively as compare control (Table 5).Increasing of plant height in response to DBS with N fertilizer may be due to the improvement of physico - chemical properties of the soil interms of water absorption for nutrient utilization of the plant. Moreover, DBS may deliver balanced micro and macronutrients as well as it was enhanced the availability of plant nutrients,which would help to speed up the metabolic activity of microorganisms and promote plant growth.The result in agreed with the findings of (34) who observed the longer plants when potato were applied with farm yard manure (13.5 tha^{-1}) and NPS (245.1 $kgha^{-1}$).lt is also harmonized with the findings of (35) who recorded maximum plant height of french bean from the application of 120 kg N ha^{-1} while, the minimum value was obtained from the control treatment. Another study that was conducted by (36) also,reflected that the highest value of mung bean plant height (78.08 cm) was recorded from the treatment which received 20:50 NP kg ha^{-1} with inoculation of Rhizobium as compared to the lowest value 68 cm on control treatment. On the other hand, a combined analysis of variance revealed that DBS with N fertilizers had not significant effects on the stem numbers of potato per hill (Table 5). This may be due to the parameter more favors for genetic makeup, physiological age and tuber seed size rather than a nutrient supplement. This finding line with the study of (37) who observed that shoot number of potato is mostly determined by the genetic makeup, the physiological age, and the size of potato seed tubers rather than mineral nutrients added in the form of fertilize

3.2.2 Plant height and spike length of wheat

Over years residual analysis of dry-bio slurry on wheat indicated that it was significantly affected both plant height and spike length at ($P < 0.05$) in (Table 5). The respective highest values of plant height and spike length (93.2 and 9.3 cm) were achieved by application of 100% DBS as compared to untreated plots (Table 5). This might be from the positive effect of DBS for delivering balanced micro and macronutrients as well as enhanced availability of plant nutrients via improving of soil properties. The result agreed with the findings of (38) who observed that the longer plants in plots which are received 75% cow dung with 25 % vermicompost than un-treated plots .lt is also harmonized with the findings of (39) who recorded that the maximum plant height (101.5 cm) value of Boro rice from the application of 5 tha^{-1} trichocompost as compared to control that was scored -the minimum value (78.6 cm) . Similarly, the result of spike length also much with the study of (40) who said that an application of 15 tha^{-1} bio-gas slurry gives the highest spike length of wheat than checked treatment or control

Table 5. Main and residual effects of dry bio-slurry with equivalence N on growth parameters of potato and wheat

Treatments	Main effect of DBS on Potato		Residual effect DBS on Wheat	
	PH (cm)	NSPH	PH (cm)	SL (cm)
Control (0,0)	29.4^{de}	3.3	68.2d	6.7d
RN (138 N $kgha^{-1}$)	52.1^{a}	4.8	89.1c	8.5c
50% DBS (5.3 tha^{-1})	28.2^{e}	4.4	92.3ab	8.7bc
100% DBS (10.6 tha^{-1})	30.4^{de}	4.4	93.2a	9.3a
75% DBS (7.95 tha^{-1})	36.6^{cd}	4.5	93.0ab	9.0abc
75% DBS+25% N (7.95 tha^{-1}+34.5 N $kgha^{-1}$)	41.9^{bc}	4.6	92.1abc	9.0abc
50% DBS+50% N (5.3 tha^{-1}+69 N $kgha^{-1}$)	42.7^{bc}	3.7	90.2bc	8.9abc
25% DBS+75% N (2.65 tha^{-1}+103.5 N $kgha^{-1}$)	48.8^{ab}	4.0	90.2bc	8.7bc
100% DBS+25% N (10.6 tha^{-1}+34.5 N $kgha^{-1}$)	44.0^{bc}	4.3	92.1ab	9.0abc
100% DBS+50% N (10.6 tha^{-1}+69 N $kgha^{-1}$)	47.1^{ab}	4.2	90.6abc	9.1ab
**LSD**	**7.9**	**NS**	3.0	0.5
**CV%**	**20.9**	**30.1**	3.6	6.6

Means followed by the same letter (s) within the column are not significantly different at ($P \leq 0.05$). DBS= dry bio slurry, N= nitrogen, PH= plant height, NSPH = number of stems per hill, SL=spike length.

3.2.3 Number of tubers per plant and total tuber yield of potato

The combined analysis of result across years and sites indicated that yield and yield a component of potato was significantly differ at (P < 0.05) by the effects of DBS and RN (Table 6). The application of 75% N with 25% DBS gives the highest fresh total tuber yield (27.6 tha^{-1}) while the lowest fresh tuber yield (8.6 tha^{-1}) was observed at control. This might be due to the releasement of nitrogen (N) from dry bio-slurry (DBS) and Urea to soil solution that makes for plant better growth and development. Moreover, it could be due to the addition of both macro and micro nutrients from the dry bio-slurry (DBS) by improving of soil pH, organic carbon, total nitrogen phosphorus and cation exchange capacity. This study is in line with the findings of (13) who revealed that, the supplying of recommended inorganic fertilizer (100kg DAP, 50kg Urea and 50kg Murate potash per hectare) with 8 tha^{-1} bio-slurry gives maximum (266.7 tha^{-1}) yield of cabbage as compare to the lowest (160 tha^{-1}) from the control treatment. And it gives about 66.7% yield increment due to the combination both bio-slurry and recommended fertilizers over control. On the other hand, the study done by (41) indicated that the lowest value of fresh shoot biomass and marketable yield of potato tuber were achieved from control while the highest values were obtained in plots that treated combined application of farm yard manure and recommended nitrogen and phosphorus.

Similarly, number of tubers per plant (NTPP) was significantly affected at (P < 0.05) by the application DBS with equi-valence nitrogen. Maximum value of NTPP was observed through the application of recommended NP as compare to control treatment (Table 6). Even if the maximum value occurs at recommended NP, the combination of both dry bio slurry and nitrogen treatment also gives better yield advantage than control. This might be due to; the harmonization of organic and inorganic fertilizers for uptake and assimilation of nutrients to potato tubers by increasing the

availability of native soil nutrients through higher biological activities. The result coincides with study of (42) who observed that an application of organic and inorganic fertilizers increased the number of tubers per plant in treated plots than control or untreated plots. Another study done by (28) showed that an application of dry bio-slurry with recommended nitrogen and phosphorus can increased the number of fruits per plant of tomato up to 40 to 73% than control

3.2.4 Grain yield and aboveground biomass of wheat

Yield and aboveground biomass of wheat was significantly affected at ($P < 0.05$) in (Table 6) by residual effect of dry bio slurry. The application of 100% DBS gives the highest grain yield (3.85 tha^{-1}) as compare to control. Similarly, addition of 75% DBS gives the highest aboveground biomass of wheat (9.59 tha^{-1}) as compare to control (Table 6). Releasing N from dry bio-slurry (DBS) to soil solution may contribute -for plant better growth and development. Moreover, it could be due to the addition of both macro and micro nutrients from the dry bio-slurry in to soil solution (rhizosphere) by increasing the availability of native soil nutrients. The result coincides with findings of (40) who reported that the supplement of 10 and 15 tha^{-1} of bio - slurry significantly increased the grain yield of wheat as compared to control. Similarly, study done by (43) revealed that the application of both biogas slurry and chemical fertilizer at 50% has a good strategy for sustainable crop yield production and soil health improvement. Similarly, the study conducted by (44) also showed that an addition of 100% dry bio-slurry significantly increased the stover and stalk yield of maize by 45.5 and 42.2% respectively than control treatments via improving of soil biological activities.

Table6.Response of potato and wheat yield parameters for dry bio slurry and equivalence nitrogen as a main and residual effect

Treatments	**Main effect of DBS on Potato**		**Residual effect DBS on Wheat**		
	NTPP	TYD t ha^{-1}	GY (t ha^{-1})	BY (tha^{-1})	1000 SW(g)
Control (0,0)	4.2^{e}	8.6^{d}	1.23d	3.11d	30.1
RN (138 N kgha^{-1})	10.9^{a}	26.2^{ab}	3.17c	7.9c	32.4
50% DBS (5.3 tha^{-1})	5.0^{de}	12.0^{cd}	3.33bc	8.37bc	32.8
100% DBS (10.6 tha^{-1})	6.4^{cd}	13.4^{c}	3.85a	9.52ab	33.6
75% DBS (7.95 tha^{-1})	7.3^{bc}	14.9^{c}	3.83a	9.59a	33.9
75% DBS+25% N (7.95 tha^{-1}+34.5 N kgha^{-1})	6.5^{bcd}	23.0^{b}	3.47abc	9.08ab	33.2
50%DBS+50% N (5.3 tha^{-1}+69 N kgha^{-1})	6.4^{cd}	24.6^{ab}	3.47abc	8.53abc	32.1
25% DBS+75% N (2.65 tha^{-1}+103.5 N kgha^{-1})	6.8^{bc}	27.6^{a}	3.28bc	8.43abc	32.6
100% DBS+25% N (10.6 tha^{-1}+34.5 N kgha^{-1})	7.0^{bc}	25.2^{ab}	3.70ab	9.56a	33.0
100% DBS+50% N (10.6 tha^{-1}+69 N kgha^{-1})	8.1^{b}	26.8^{a}	3.59abc	9.06abc	32.4
**LSD**	1.7	3.7	0.5	1.2	NS
**CV%**	25.6	19.4	15.6	14.8	10.1

Means followed by the same letter (s) within the column are not significantly different at ($P \leq 0.05$). DBS= dry bio slurry, N= nitrogen, PH= plant height, NTPP = number of tubers per plant, TYD, = total tuber yield, GY= grain yield, BY= above ground biomass and SW = seed weight.

3.3 Economic Analysis

Net benefits were done by calculated current fertilizer (Urea) cost of 0.27 US$ kg^{-1}, cost of DBS kg^{-1} 0.004 US$, field price of potato was 0.12 US$ kg^{-1}, cost of labor per man day in the area is 1.4 US$. The marginal rate of return 100% was used to determine the acceptability of treatments.

The economic analysis indicated that most of treatments give high net benefit than control (Table 7). Addition of 25% DBS with 75% RN gave 2889.2 US$ net benefit with 4486.3% marginal rate of return. This replied that for every 1 US$ invested for 25% DBS with 75% RN in field, farmers can obtain an additional 44.863 US$ (22). All Un-dominated treatment rates could be acceptable for potato producers in the study area except the dominated one's. Therefore, the most economical rate for producers with low cost and higher benefit was 25% DBS with 75% RN.

Table 7. Partial budget and marginal analysis of potato as affected by the application of dry bio-slurry with nitrogen at Yilemana Densa District.

Treatments (RN +DBS kgha^{-1})	10% Adjusted tuber Yield t ha^{-1}	Total variable Cost US$ha^{-1}	Net Benefits US$ ha^{-1}	MRR%
Control	7.74	0	928.8	-
50% DBS	10.8	35.2	1260.8	943.2
75% DBS	13.41	52.8	1556.4	1679.6
100% DBS	12.06	70.4	1376.8	D
50% DBS+50% N	22.14	84.5	2572.3	D
25% DBS+75% N	24.84	91.6	2889.2	4486.3
100% DBS+25% N	22.68	95.1	2626.5	D
RNP	23.58	98.7	2730.9	D
100% DBS + 50% N	24.12	119.7	2774.7	D
75% DBS + 25% N	20.7	217.5	2266.5	D

RNP=percent of recommended nitrogen and phosphorus in kg per hectare, DBS=dry bio-slurry in kilogram per hectare, MRR= marginal rate of return; D= is dominated treatments

4. Conclusion and Recommendation

Yield and yield components of potato and wheat were significantly affected by main and residual effect of dry bio slurry and nitrogen substitution. Both crops productivity were improved by substitution of dry bio-slurry and nitrogen as compared to control. Applied dry bio-slurry with N (nitrogen) had a positive impact on plant height, number of tubers per plant, total tuber yield, grain

yield and above ground biomass. Substitution of nitrogen by Dry bio-slurry rate in most treatments gives higher net benefit as compared to control. Among treatments undominated once gave better net benefit with acceptable marginal rate of return than control. Therefore, small-scale farmers can benefited, if they apply these soil improvement rates depending on their convenience. Related to this application 25% DBS with 75% N was given the highest net benefit with 4486.3% marginal return. So that, use of 25% DBS with 75% RN could be promoted for the production of potato and wheat in the study area. For the future, similar studies should be done across locations and crops, in permanent plots for sustainable crop production and soil health enhancement .

Competing of interest

There is no conflict of interest between Authors

Acknowledgment

The author is grateful to Adet Agricultural research center for its administrative and budget support

Financial disclosure

The research supported by budget by Amhara Agricultural Research Institute (ARARI) and the funder hand no role in data collection and designs and none of the authors had not receive salary from the funder

Reference

1. Brankatschk,G, Finkbeiner, M. Modeling crop rotation in agricultural LCAs - challenges and potential solutions. Agric.Syst.2015;138,6676.https://doi.org/10.1016/j.agsy.2015.05.008.

2. Nemecek, T., Hayer, F., Bonnin, E., Carrou´ee, B., Schneider, A., Vivier, C. Designing eco-efficient crop rotations using life cycle assessment of crop combinations. Eur. J. Agron.2015; 65, 40–51. https://doi.org/10.1016/j.eja.2015.01.005.

3. Birch PRJ, Bryan G, Fenton B, Gilroy EM, Hein I and Jones JT. Crops that feed the world 8: potato: are the trends of increased global production sustainable? Food Secur.2012;4(4):477–508. https://doi.org/10.1007/s12571-012-0220-1.

4. CSA (Central Statistical Agency). Agricultural sample survey 2019/20 (2012 E.C) volume I. Report on area and production of major crops meher season crops for private peasant holdings. 2020;CSA, Addis Ababa.

5. CSA (Central Statistical Agency). Agricultural sample survey: Area and production of major crops. 2021; Addis Ababa, Ethiopia.

6. FAOSTAT. Food and Agriculture Organization corporate statistical database of the United Nations. 2021; Retrieved from http://www.fao.org/faostat/en/#data/QC

7. Efrem B, Hirut K, Getachew B. Durum wheat in Ethiopia: an old crop in an ancient land. 2000;Addis Ababa: Institute of Biodiversity Conservation and Research.

8. Chanie, C., Teshome, T., Temesgen, T., & Berihun, B. Characterization of potato production, marketing, and utilization in North Western Amhara Region, Ethiopia. Journal of Horticulture and Forestry.2017;9(3), 17–25. doi:10.5897/JHF

9. Shankarappa TH. Gurumurthy SB, Patil SV and Lokesh MS. (2012). Influence of phosphorus enriched biogas spent slurry (BSS) on growth and yield of sunflower (Helianthus annuus). Journal of Plant Science.2012; 7: 253-258

10. Khan SA, Malav LC, Kumar S, Malav MK and Gupta N Resource utilization of biogas slurry for better yield and nutritional quality of baby corn. Advances in Environmental and Agricultural Science.2015;382-394.

11.Nitesh Samadhiya, SP Singh, Girish Goyal, Murlidhar J Sadawarti, SK Sharma and LK Tripathi. Evaluation of organic and inorganic sources of nutrients in maize (Zea mays L.): Potato (Solanum tuberosum L.) cropping system. The Pharma Innovation Journal.2021;10(7):1360-1365.

12. S. Bilkis, M.R. Islam*, M. Jahiruddin, M. M. Rahman and T.S. Hoque. Residual effects of different manures and fertilizers applied to preceding potato crop on succeeding mung bean (vigna radiate l.) crop in potato-mung bean-rice cropping pattern. SAARC J. Agri.2018;16(2):167-179.

13. Yalemtsehay D and Fisseha I. Comparative study on the effect of applying biogas slurry and inorganic fertilizer on soil properties, growth and yield of White Cabbage (Brassica oleracea var. capitata f. alba). Journal of Biology, Agriculture and Healthcare. 2016;6(19).

14. Karki KB. Estimation of plant nutrient loss from biogas slurry. Biogas Support Programme.1997;

15. Errebhi, M., Rosen, C.J., Gupta, S.C. and Birong, D.E. 1998. Potato Yield Response and Nitrate Leaching as Influenced by Nitrogen Management. Agron. J. 1998;90:10–15.

16. Alva, A. 2004. Potato nitrogen management. J. Veg. Crop Prod.2004; 10: 97-130.

17. White, P.J., Wheatley, R.E., Hammond, J.P. and Zhang, K. Minerals, soils and roots. In: Vreugdenhil D (ed) Potato biology and biotechnology, advances and perspectives. Elsevier, Amsterdam.2007;pp: 739-752.

18. Zelalem, A., Tekalign, T. and Nigussie, D. Response of potato (Solanum tuberosum L.) to different rates of nitrogen and phosphorus fertilization on vertisols at DebreBerhan, in the central highlands of Ethiopia. African journal of plant science.2009;3: 16-24.

19. Rhaman M.S., Kibria M.G., Hossain M., and Hoque M.A. Effects of organic manure and bio-slurries with chemical fertilizers on growth and yield of rice (cv. BRRI dhan28). Int. J. Expt. Agric.2016;6 (3): 36-42

20. Eyasu E . Soils of the Ethiopian highlands: Geomorphology and Properties. CASCAPE Project, ALTERA, Wageningen University and Research Centre (Wageningen UR), the Netherlands.2016; p. 385

21. Sahlemedihn S and Taye B. Procedures for soil and plant analysis.2000; Addis Ababa, Ethiopia.

22. CIMMYT. From Agronomic Data to Farmer's Recommendation: an Economic Training Manual. 1988;Revised Edition. Mexico, D.F.

23. Gomez K.A and A.A. Gomez. Statistical procedures for agricultural research, 2nd edition. John Wiley and Sons, New York. International Congress Series.1984; 1291: 42-52.

24. Tekalign T. Soil, plant, water, fertilizer, animal manure and compost analysis. Working Document No. 13. 1991;International Livestock Research Center for Africa, Addis Ababa.

25. Olsen S.R, Cole C.V, Watanabe F.S and Dean L.A. Estimation of available phosphorus in soils by extraction with sodium carbonate. USDA Circular.1954;939: 1-19.

26. Landon J.R. Booker tropical soil manual: a handbook for soil survey and agricultural land evaluation in the tropics and subtropics. New York;1991.

27. Workineh E,Yihenew G, Eyasu E and , Matebe D.. Integrated fertilizer application improves soil properties and maize (Zea mays L.) yield on Nitisols in Northwestern Ethiopia. Journal of Helyion.2021;7-e06074

28. Geremew B , Girma A and Birhanu B. Effects of dry bioslurry and chemical fertilizers on tomato growth performance, fruit yield, and soil properties under irrigated conditions in Southern Ethiopia.African Journal of Research.2019; 14(33), pp. 1685-1692.

29. Tsegaye T, Tewodros A and Hussien M.2018. Combined Application of Bioslurry and Inorganic Fertilizers on Quality Traits of Cabbage and Soil Properties. Asian Journal of Biological Sciences.2018;11 (1): 24-32

30. Zelalem A, Tarekegn & Hussien M.2020. Effect of liquid bio-slurry and nitrogen rates on soil physico-chemical properties and quality of green bean (Phaseolus vulgaris L.) at Hawassa Southern Ethiopia.Journal of plant interaction.2020;15 (1):207–212.

31. Sandeep S & Salwinder S.D. Effect of Sewage Sludge and Rice Straw Compost on Yield, Micronutrient Availability and Soil Quality under Rice–Wheat System. Journal Communications In Soil Science and Plant Analysis.2019; DOI:10.1080/00103624.2019.1648489.

32. Shahzad, K., A. Khan, J.U. Smith, M. Saeed, S.A. Khan and S.M. Khan. Residual effects of different tillage systems, bio slurry and poultry manure on soil properties and subsequent wheat productivity under humid subtropical conditions of Pakistan. International journal Biosci.2015; 6(11): 99-108.

33. Tamado T, Mitiku W. 2017. Effect of combined application of organic and mineral nitrogen and phosphorus fertilizer on soil physico-chemical properties and grain yield of food Barley (Hordeum vulgare L.) in Kaffa Zone, South-western Ethiopia. MEJS.2017; 9(2):242–261.

34. Melkamu A, Minwyelet J, Tadele Y & Masho A.2020. Integrated application of compound NPS fertilizer and farmyard manure for economical production

35. Moniruzzaman M, Halim G.M.A and Firoz Z A. Performances of the French bean as influenced by plant density and nitrogen application. Bangladesh. JAR.2009;34(1): 105-111.

36. Muhammad U.C, Muhammad U.H, Imran K. Effect of different nitrogen and phosphorus fertilizer levels in combination with nitrogen and phosphorus solubilizing inoculants on the growth and yield of mung bean. PJLSS. 207;15(1): 31-36.

37. De La, I., Guillen, I. A., & Del Moral, L. F. G. Yield development in potatoes as influenced by cultivar and the timing and level of nitrogen fertilization. American Potato Journal,1994; 71, 165–173. Doi: 10.1007/ BF02849051

38. Balasubramanian A, P. Stalin, M.Saravanaperumal and S.R.Vinoth kumar. Residual effect of Integrated Nutrient Management practices on growth and yield of rice fallow blackgram (Vigna mugno L.). Journal of Emerging Technologies and Innovative Research,2016; 3(5): 2349-5162.

39. Bilkis S, M.R. Islam, M. Jahiruddin, M.M. Rahaman. Integrated Use Of Manure And Fertilizers Increases Rice Yield, Nutrient Uptake And Soil Fertility In The Boro-Fallow-T.Aman Rice Cropping Pattern. SAARC J. Agri,2017; 15(2): 147-161.

40. Pandey B.P, N. Khatri, M. Yadav, K. R. Pant, R. P. Poudel, and A. H. Khan. Effect of Digestate /Biogas Slurry In Wheat Under Rice – Wheat Cropping System. Journal of Agriculture and Forestry University.2020;4: 67-75.

41. Tsegaye G,Birhanu B, S B & Berga L.2020. Integrated Application of Organic and Blended Mineral Fertilizers Improves Potato Productivity and Income for Smallholder Farmers in Acidic Soils. Journal of fertilizer and pesticides.2020;8(3),2471-2728.

42. Haque M.A, M. Jahiruddin, M. S. Islam, M. M. Rahman, M. A. Saleque. Effect of Bio slurry on the Yield of Wheat and Rice in the Wheat– Rice Cropping System. Journal of Agric Res.2018. 7(4):432–442.

43.Shahid, M., A. K. Shukla, P. Bhattacharyya, R. Tripathi, S. Mohanty, A. Kumar, B. Lal, P. Gautam, R. Raja, B. B. Panda., et al. Micronutrients (Fe, Mn, Zn and Cu) balance under long-term application of fertilizer and manure in a tropical rice–rice system. Journal of Soils and Sediments.2016; 16 (3):737–47. doi: 10.1007/s11368-015- 1272-6.

44. Mercy K, Esther M, James N, Kevin K, Stephen Mailu,Peterson Njeru and Rewe Thomas.2022. Integrated effect of liquid bio slurry and inorganic fertilizer on selected soil chemical properties, maize (Zea mays) growth, yield and grain quality. African Journal of Crop Science, 2022;10 (5): 001-016.

45.Liu, E., Yan, C., Mei, X., He, W., Bing, S.H., Ding, L.Long-term effect of chemical fertilizer, straw, and manure on soil chemical and biological properties in northwest China. Geoderma .2010;158, 173–180

46. Efthimiadou A, Bilalis D, Karkanis A and Froud W.B. Combined Organic and Inorganic Fertilization Enhances Soil Quality and Increased Yield, Photosynthesis and Sustainability of Sweet Maize Crop. AJCS.2010; 4(9):722-729.

47. Tadele Amare, Zerfu Bazie, Erkihun Alemu, Beamlaku Alemayehu, Abere Tenagne, Bitewlgn Kerebh, Yasin Taye, Abrham Awoke, Tesfaye Feyisa, Selamyihun Kidanu. Yield of potato (Solanum tuberosum L.) increased by more than two-folds through nitrogen and phosphorus fertilizers in the highlands of North-Western Ethiopia. Journal of Heliyon.2022;8(11111).

Response to Authors

Dear respected editorial we have tried to adjust the manuscript by tracking, coloring based on your comments and without tracking

And here we also put the general response to the author's comment

**Comments**	**Response**
**Reviewer 1**	**Authors**
I. The title shall be revised by considering the replacement/substitution of chemical N with DBS<br>II. Statement of the problem in the abstract requires more articulation,<br>III. Please review previous research findings/practices that showed the improvement of growth and yield of crops and soil properties through application of organic and inorganic fertilizers (only general principles are reviewed).<br>IV. Correct the net plot area and MRR, which create deviations in the collected data and conclusion and recommendation<br>V. The recommendation in the abstract and in conclusion is different. Please revise and make a similar recommendation in both sections of the manuscript.<br>VI. Revise grammatical and editorial errors throughout the document	I. We have been adjusting the title based on reviewer 4 suggestion **"Effects of dry bio slurry with nitrogen fertilizer on yields of potato and wheat under short term crop rotation system** by considering the comment of reviewer 1<br>II. Here we are done the re -articulation of for statement of the problem in the abstract part based on the reviewer comment<br>III. Based on the comment we in corporate the previous findings that deals the integration effect of organic and inorganic fertilizers on growth and soil properties<br>IV. Here we correct both MRR and net plot area-based reviewer comment<br>V. We have been making it similar in both parts of the manuscript for recommendation<br>VI. Here we want to appreciate all of the reviewers including reviewer1 for their effort as manuscript be

	well edited by language writing. by fully considering and give attention for there frequent comment we have correct and check it by fluent speaker and language skilled persons in addition to our correction effort .so the manuscript well edited and corrected in terms of language
**Comments**	**Response**
**Reviewer 4**	**Authors**
I. Suggested title: Effects of dry bioslurry with nitrogen fertilizer on yields of potato and wheat under short term crop rotation system<br>II. The abstract lacks short background of the problem.<br>III. The result section in the abstract is not well written. Better to summarize the key results only.<br>IV. There are too long sentences in the paper (see the first four lines of the abstract, description of study area), difficult to realize the concept.<br>V. The language is generally poor. It should be further improved by subject experts.<br>VI. There are editorial errors like spacing (2.2.2Gudenie potato variety, 2.2.3TAY variety of Wheat), spelling (List significant difference (LSD).	I. We have been adjusting the title based on reviewer suggestion "**Effects of dry bio slurry with nitrogen fertilizer on yields of potato and wheat under short term crop rotation system**<br>II. we incorporate the background of the problem in abstract part based on this comment (reviewer 4).<br>III. we are correct it in both parts by summarize.<br>IV. we make clear and short in most lines and sentences in the manuscript.<br>V. Here we want to appreciate all of the reviewers including reviewer4 for their effort as manuscript be well edited by language writing. by fully considering and give attention for their frequent comment we have correct and check it by fluent speaker and language

VII. SAS software version 9.4 is not available during 2002. You have cited the correct releasing year.<br>VIII. In table 2, describe the clay, sand and silt contents and remove repeated rows containing names of soil properties.	skilled persons in addition to our correction effort .so the manuscript well edited and corrected in terms of language.<br>VI. We correct it by 2014.<br>VIII. we also correct it based on the comment
