# Supplementary material for: Effects of dry bio-slurry and nitrogen fertilizer on potato and wheat yields under rotation cropping system
Source: PLoS One. 2024 Jul 31;19(7):e0306625. doi: 10.1371/journal.pone.0306625 (PMC11290689; doi:10.1371/journal.pone.0306625)
Supplement: Attachment — Submitted filename: Commented manuscript PONE-D-23-10312.docx [file pone.0306625.s001.docx]

**Main and residual effect of dry bio slurry on soil properties and crop production under Potato-wheat system**

Zelalem Addis*^1^,Tadele Amare^1^, Bitewlgn Kerebih^1^, Antenh Abewa^1^, Tesfaye Feysa^2^ ,Abrham Awoke^1^ and Abere Tenagne^1^

1 Adet Agricultural Research Center P.O.BOX 08, Bahir dar Ethiopia

2 Amhara Agricultural Research Institute

*Corresponding Author zelalemaddis660@gmail.com

**ABSTRACT**

The experiment was conducted to determine the main and residual effect of dry bio-slurry with equivalence N rates on soil properties and yield components of Potato and wheat in the potato-wheat cropping system at Yelmana Denesa district. The experiment comprising ten levels of treatments (Control, RNP, 50%DBS, 100%DBS, 75%DBS, 75%DBS+25%RN, 50%DBS+50%RN, 25%DBS+75%RN, 100%DBS+25%RN, and 100%DBS+50%RN) were laid out in RBD design with three replications for three years. Data on soil Physico-chemical properties and yield components of potato and wheat were collected and subjected to ANOVA using SAS software. The results indicated that the application of different rates of dry bio-slurry with N fertilizer significantly affected the yield components of both potato and wheat. Soil property except pH is not significantly affected by the main effect of dry bio-slurry. Applying 25% DBS with 75 % RN gives the highest tuber yield (27.6 tha-1) compared to the control. Similarly, an application of sole 100% and 75% dry bio-slurry scored the highest grain yield (3.85tha^-1^) and above-ground biomass (9.59tha^-1^) respectively due to the residual effect on wheat. Similarly, 25%DBS with 75% RN scored the highest net benefit with acceptable marginal return (above 100%) next to 50%DBS with 50% RN. Generally, the application of dry bio-slurry with nitrogen rates can be improved the yield and yield components of potato and wheat in the study area. To attain the highest net benefit, as the main effect of DBS within a short period of time application of 50%DBS with 50%RN can be preferable for yield improvement in the study area and similar agro ecological environments. But to be consistent further research should be done widely and repeatedly in the areas in permanent plots.

Key Words: dry bio-slurry, nitrogen, soil properties, potato, wheat

1. **Introduction**

Potato (Solanum tuberosum) is the world’s third most important food crop after wheat and rice (Birch et al., 2012). The mean world potato yield In Ethiopia, the area under potato cultivation reaches about 73,677.64 ha and its production was 1,044,436.359 tons (CSA, 2020). The productivity of potato in Ethiopia is 13.9 t ha^-1^ (CSA, 2018), which is relatively low compared to other African countries (FAOSTAT, 2017). Bread wheat (Triticum aestivum L.) is also is one of the major cereal crops grown in the highlands of Ethiopia, and the country is regarded as the largest wheat producer in Sub-Saharan Africa (Efrem et al, 2000). Out of the total grain crop area, wheat ranked 4th after tef (Eragrostis tef), maize (Zea mays) and sorghum (Sorghum bicolor), while third in total production after maize and tef (CSA, 2016). Despite the long history of wheat cultivation and its importance to Ethiopian agriculture, its average yield is still very low, not exceeding 2.4 t ha-1 (CSA,2014) which is below the world’s average of 3.4 t ha^-1^ (FAOSTAT, 2015 this phenomenon is common in our region.

Low level of potato-wheat productivity is mainly due to soil fertility degradation, improper fertilization, poor pest management practices, use of the low-quality seed, and soil nutrient depletion (Chanie *et al*., 2017). Enhancing fertility is a precondition for a practical crop production system through organic manure like bio-slurry that can achieve sustainability soil fertility and crop production (Shankarappa *et al*., 2012; *Khan et al*., 2015). The bio-slurry that obtained after extraction of the energy content of animal manure is an excellent fertilizer, rich in major nutrients (nitrogen, phosphorous and potassium) and organic matter that determine the soil fertility and yield of different crops and vegetables (Yalemtsehay and Fisseha, 2016). It also improves the physical and biological quality of soil besides providing both macro and micro-nutrients to crops and vegetables. The application of bio-slurry also helps in the reduction of dependence on mineral fertilizers (Karki, 1997). Because both Potato and wheat are highly responsive to N fertilization; it is usually the most limiting essential nutrient for growth and development (Errebhi *et al*.,1998).In addition, supplying Nitrogen plays an important role in the balance between vegetative and reproductive growth for both crops (Alva, 2004; White *et al*., 2007). Various studies have shown that N fertilizer applications can increase the dry matter-protein content of wheat and potato tubers (Zelalem *et al*., 2009). Furthermore, most of the time the available nitrogen in most organic source fertilizers including bio-slurry is high as compared to other nutrients in concentration (). Due to this dry bio-slurry and nitrogen fertilizers were applied through nitrogen equivalence. Neither organic manure nor chemical fertilizer alone is not enough to meet the demand for crops in the different cropping system via soil-plant interaction (Rahman, 2016). Because of this, the study was done to determine the main and residual integrated effects of dry-bio slurry and nitrogen fertilizer on potato–wheat cropping system for production and soil physic-chemical properties at Yilemana Densa district with flowing objectives to determine the optimum rate of dried bio-slurry and nitrogen fertilizer on potato and wheat yield and Physico-chemical properties improvement in northwestern Amhara region Ethiopia

2. **Material and Methods**
   2.1 **Description of Study Area**

The experiment was conducted at Yilemana Densa district on Farmer’s field for three consecutive years 2019/20-2021/22 panting seasons at Debremewi Keble West Gojam zone North Western Amhara Region Ethiopia. The site is located 33km eastern away from Bahirdar. Geographically the area lies at 11^0^ 21' 22'' N and 37^0^ 25' 43'' E (Figure 1) with a mean altitude of 2304 m above sea level. It receives a mean annual rainfall of 1421 mm with mean minimum and maximum temperatures of 12.29 and 27.56°C, respectively Bureau of Agriculture (BOA). The landforms of the area are characterized by undulating to rolling plateaus, scattered moderate hills, dissected side slopes, and river gorges (Eyasu, 2016). Based on the district Bureau of Agriculture, the major land use comprises cultivated land (57%), forest and bushes (2%), grazing land (33%), and others (8%). Major crops, grown in the study area are Maize, Tef, wheat, barley, potato, and field pea which take the lion’s share. Soil types in the area are Nitisols (45%) Vertisols (30%) and Luvisols (25%). This on-farm experiment was conducted on Luvic Nitisols which is the most dominant soil in the study area

2.2 **Experimental design and experimentation**

The experiment was laid out in Randomized Complete Block Design (RCBD) with three replications which has ten treatments control (without N), recommended N (138N),dry-bio slurry 50% equivalence (5.3tha^-1^),dry-bio slurry 100% equivalence (10.6 tha^-1^), dry-bio slurry75%equivalence(7.95 tha^-1^),dry-bio slurry75% equivalence (7.95tha^-1^)+25% recommended N (34.5N), dry-bio slurry 50% equivalence (5.3tha^-1^)+50% recommended N (69N),dry-bio slurry 25% equivalence (2.65tha^-1^)+75% recommended N(103.5N),dry-bio slurry100% equivalence (10.6tha^-1^)+25% recommended N(34.5N) and dry-bio slurry 100% equivalence (10.6 tha^-1^) +50% recommended N (69 N). The rates of dry-bio slurry were adjusted based on recommended rate of N equivalency corresponding to its N content. Urea was used as a source of synthetic N whereas P was applied in the form of TSP to all plots. The experiment was carried out under rain-fed conditions *Gudenie* and Tay varieties were used as test crops for Potato and wheat respectively. Total area of each plot was 4.5 m x 3 m (13.5 m^2^) having 1m space between plots and 1.5m between blocks. The potato was spaced 0.3m between plants and each plot consisted of six rows at 0.75m intervals and the data were collected from the middle four rows. Wheat was planted as a rotated crop in 0.2m and harvested from middle rows by avoiding four rows as a border. Dry bio-slurry and P_2_O_5_ were applied during the planting period of time as basal whereas inorganic N for potato was applied in three; one-third third at planting, one-third about 30 days after planting, and the remaining one-third at the beginning of the flowering. While for wheat recommended NP was applied in all plots except control which only received P_2_O_5_ to see the residual effect of dry bio-slurry in the next crop and nitrogen was supplied, in two split.

2.3 **Data Collection, Preparation, and Analysis**
    2.3.1 **Dry- bio slurry analysis**

Representative composite sample dry-bioslurry was taken from the whole collected dried pit of slurry that was collected from different biogas plants across individual farmers for analysis of (pH), organic carbon (OC%), cation exchange capacity (CEC), total nitrogen (TN%) and available phosphorus (P) by following laboratory procedures (Sahlemedhin and Taye, 2000).

Table 1. Physico-chemical analysis of dry-bio slurry before incorporation in year1and 2 and its average

| Dry bio-slurry | Concentration |
| --- | --- |
| Year 1 |  |
| Dry matter% | 11.2 |
| OC% | 27.8 |
| TN% | 1.2 |
| Av P (mg kg^-1^) | 112.1 |
| pH (H_2_O;1:2.5) | 7.9 |
| C:N ratio | 22.9 |
| CEC (cmol kg^-1^) | 59.8 |
| Year 2 |  |
| Dry matter% | 11.8 |
| OC% | 16.3 |
| TN% | 1.4 |
| Av P (mg kg^-1^) | 91.6 |
| pH (H_2_O;1:2.5) | 7.7 |
| C:N ratio | 11.6 |
| CEC (cmol kg^-1^) | 67.4 |
| Average result of Year1andYear 2 |  |
| Dry matter% | 11.5 |
| OC% | 22.1 |
| TN% | 1.3 |
| Av P (mg kg^-1^) | 101.9 |
| pH (H_2_O;1:2.5) | 7.8 |
| C: N ratio | 17.3 |
| CEC (cmol kg^-1^) | 63.6 |

OC%=organic carbon percent, TN%=total nitrogen percent, C: N=carbon to nitrogen ratio, CEC = cation exchange capacity, AvP=available phosphorus, and pH= Power of hydrogen concentration.

2.3.2 **Soil sampling and analysis before planting**

Before planting, representative soil samples were collected from 0-20 cm depth in a random sampling method from 10 spots in the field by using an auger. All samples were mixed together and one composite sample was formed. The composite sample was grounded using a mortar and passed through a 2 mm sieve for analysis of soil texture, CEC, pH, and available P; whereas a 0.5 mm sieve was used for determining the organic carbon (OC) and total N. Bulk density was determined by the core sampling method.

2.3.3 **Soil sampling and analysis after harvest in potato–wheat cropping system**

Determination of the particle size distribution was done by using the hydrometer method (procedures) compiled by Sahlemedhin and Taye (2000) and the sand, silt, and clay percent’s were calculated and identified using FAO textural triangle. The major chemical properties of soil such as OC, pH, CEC, total N and available P were analyzed following the compiled laboratory manual of Sahlemedhin and Taye (2000). Soil pH was measured in water at the ratio of 1:2.5 using glass electrode pH meter. The soil OC content was determined following the wet digestion method as outlined by Walkley and Black which involves the digestion of OC in the soil samples with potassium dichromate (K_2_Cr_2_O_7_) in a sulphuric acid solution. AvP was determined by Olsen extracting method. The total N content in the soil samples was determined following the Kjeldahl method. CEC was determined by extracting the soil samples with ammonium acetate (1NNH_4_OAc) followed by repeated washing with ethanol (96%) to remove the excess ammonium ions in the soil solution. Percolating the NH4^+^ saturated soil with sodium chloride would displace the ammonium ions adsorbed in the soil and the ammonium liberated from the distillation was titrated using 0.1N NaOH.

**Crop data collection**

Growth parameters

Plant height, number of plants per hill, and spike length were measured for both potato and wheat at the maturity stage by taking five randomly selected plants from ground level to the top apex and averaging for a single reading.

**Yield and yield components**

The number of tubers per plant potato was taken by counting tubers from five randomly taken plants at the maturity stage from the middle rows of experimental plots and averaged for a single estimation of the mean value. While thousand seed weight for wheat was taken from each treatment plot by counting of 1000 seeds. Total tuber yield, grain yield, and above-ground biomass were measured at harvesting from the middle plot area by avoid border effects.

2.4 **Economic Analysis**

Because potato is the main crop in this system the economic analysis was done based on its. Economic analysis was performed to make rational choice among the applied variables in the production of potato. The partial budget and marginal rate of return (MRR) were used for evaluating the change in farming methods that affect partially rather than the whole farm practice and also concerned with planning tool to estimate the profit change within a farm (CIMMYT, 1988). This was computed by adjusting yield downward by a 10% and multiplying it with the local field price (6 Ethiopian Birr per kg of potato). Dominance analysis was done by listing of treatments in an increasing order of cost and that has net benefit less than or equal to treatments with the lower costs that vary is dominated (CIMMYT, 1988).

2.5 **Statistical Analysis**

All data were subjected to analysis of variance through GLM procedure by using SAS software program version 9.4 (SAS Institute, 2002). List significant test (LSD) at 0.05 probability level was employed to separate treatments means where significant differences exist (Gomez and Gomez, 1984).

**3. Result and Discussion**

**3.1 Soil Chemical Properties before planting and After Harvesting of potato**

The results of soil chemical properties before and after harvest from each experimental site are indicated in Table 2, 3 & 4. The before-planting results showed that the soil was acidic in reaction with a pH (H_2_O 1:2.5) value of 5.1Y1S1 and 5.2Y2S1, which is within the range of soil pH for potato production Tekalign (1991) (table 2). The total N, available P, OC, C: N ratio and CEC of the soil Y1S1 and Y2S1 before planting were (0.16&0.16%), (6.9&6.3 mg kg^-1^), (1.8 &1.5%),(11.1&9.3) and (33.9&30.3cmol (+) kg^-1^), respectively (Table 2). The total N content of the soil was within the range of medium according to Tekalign (1991) who classified the range of total N < 0.1, 0.1- 0.15, 0.15-0.25 and > 0.25% as very low, low, medium and high, respectively. Olsen *et al*. (1954) classified available P content of the range < 5 as very low, 5 -15 as low, 15 -25 as medium and > 25 mg kg^-1^ as high. Hence the available P of the soil before planting lies under the low range. According to Landon (1991) the soil OC content ranges of 1-2, 2-4, and 4-6% are rated as low, medium and high, respectively while cation exchange capacity (CEC) ranges of 5-15, 15-25 and 25-40 cmol kg^-1^ are rated as low, medium and high, respectively.

Based on these ratings the OC (1.4 &1.5%) and CEC (33.9&30.3 cmol kg^-1^) before planting of the experimental fields were in the low and high ranges, respectively. Generally, the nutrient contents of the study site Y2S1 is not good in terms of availability of major plant nutrients beside its nice CEC. On the other hand on Y2S2 pH, TN, avP, OC, C: N and CEC pre planting values were 5.5, 0.17%, 6.8ppm, 2.4%, 14.1 and 33.9 respectively. Based on Tekalign (1991) rating the pH value was under moderate while total nitrogen was medium. Similarly, according to olsen *et al*. (1954) available P was under low while OC and CEC were medium and high respectively landon (1991). Y2S2 rather than Y1S1and Y2S1 have good soil fertility status based on its values of soil chemical properties. However, the acidity of soil Y1S1andY2S2 may be cause the sorption of available P. Thus, application of OM like bio-slurry is very essential in order to neutralize soil solution for the availability of nutrients. On the other hands, after harvest all soil chemical properties except soil pH (on Y2S2) were not affected by the application of dry bio-slurry with N equivalence rates (Table3).

The non-significant effects of the applied treatments on these parameters might be due to the slow release of nutrients from dry bio-slurry that was applied during the experimentation period to soil solution related to short cropping season potato cultivation. However, applied dry bio-slurry and N rates had a significant (*P* < 0.05) effect on pH at site2 in 2020-21soil after harvest as compared to the control (Table 2). Numerically the highest soil pH content, was obtained by the application of 100% and 100%+25%N dry bio-slurry as compared to the control (Table 3). The increment of soil pH in treated plots may be related due to the releasing of basic cations from dry bio-slurry into soil solution that makes substitute of acid cations.These results were in agreement with the investigation of Workineh *et al.* (2021) who reported that the combined application of compost with inorganic NPSB in maize increased the soil pH content after harvest as compared to control.

**3.2 Residual effect of dry bio-slurry on soil chemical properties after harvesting of wheat**

The residual effect of dry bio-slurry on selected soil chemical properties was significantly explained at (p<0.05) across sites and years in table 4. Related to this an addition of 100% (10.6tha^-1^) dry bio-slurry gives the highest TN% (0.207and 0.223) on Y1S1 and YS2 respectively than the control. This might be due to the gradual releasing of Nitrogen dry bios-slurry into soil solution beyond its chelating capacity. The finding lined with Geremew *et al*., 2019 who found that an application of dry bio-slurry with inorganic fertilizers gives the maximum value of total nitrogen than control. Similarly, the study conducted by Tsegaye *et al*., 2018 also indicated that an application of 70cm^3^ scored the highest (1.36%) TN as compared to the control which gives 0.07%

On the other hand, OC, C: N and CEC are significantly affected at Y2S1(Table 4) While in Y2S2 only CEC is significant at (p<0.05) Table 4.Based on this numerically the highest value of CEC(34.6), C: N (12.82)&OC% (2.20) was observed by application 50%DBS and 100% DBS with 25%N as compared to control on Y2S1.On Y2S2 the maximum value of CEC(cmolkg^-1^) 36.06 was obtained in plots that receive 100%DBS with 25%N than control. This might be due to the positive effect applied to DBS that enables increasing organic matter and holding capacity of positive cation in the soil exchangeable site. The finding agreed with Zelalem *et al*., 2020 who revealed that an application of 41.3m^-3^ liquid bio-slurry with 20.5kgha^-1^N significantly increased soil organic carbon than untreated plots. The study conducted by Sandeep and Salwinder (2019) also indicated that an application of 10tha^-1^ with 50%Nkgha^-1^ significantly increased OC% by scoring the maximum value of 0.67 than the control. Organic amendments significantly enhanced SOC thus had a considerable effect on soil microbes and nutrient availability and uptake this may alter the C: N ratio. However, the addition of external organic matter with a low C: N ratio may induce the mineralization of OM. This makes nitrogen trapped by organic matter; a phenomenon known as the priming effect (Shahzad *et al*. 2015). The application of the 50% DBS and 100% DBS with 25%N gives the highest CEC at Y2S1 AND Y2S2 respectively as compared to the control (Table 1) Such increment in CEC might be due to the application of DBS on soil that makes a negatively charged colloidal site and storehouse of basic cations. The finding agreed with Tamado and Mitiku (2017) who reported that the use of organic FYM and inorganic fertilizers significantly increased CEC over the control.

Table 2.Soil physico-chemical properties before planting

| 2019-20 site1(Y1S1) | | | | | | | |
| --- | --- | --- | --- | --- | --- | --- | --- |
| Texture | BD | pH | TN% | Av P (ppm) | CEC **(**cmol kg^-1^) | C:N | OC% |
| SCL | 1.22 | 5.1 | 0.16 | 6.9 | 33.9 | 8.8 | 1.4 |
| 2020-21 site1(Y2S1) | | | | | | | |
| Texture | BD | pH | TN% | Av P (ppm) | CEC (cmol kg-1) | C:N | OC% |
| SCL | 1.33 | 5.2 | 0.16 | 6.3 | 30.3 | 9.3 | 1.5 |
| 2020-21 site2 (Y2S2) | | | | | | | |
| Texture | BD | pH | TN% | Av P (ppm) | CEC (cmol kg-1) | C:N | OC% |
| SCL | 1.26 | 5.5 | 0.17 | 6.8 | 33.9 | 11.8 | 2.0 |

NB: SCL= sandy clay loam; BD= bulk density

Table 3.main effect of dry bio slurry with nitrogen on soil chemical properties after harvest of potato

| 2019-20 site1(Y1S1) | | | | | | | | | |
| --- | --- | --- | --- | --- | --- | --- | --- | --- | --- |
| Treatment | pH | TN% | Av P (ppm) | CEC **(**cmol kg^-1^) | | C:N | | | OC% |
| Control (0,0) | 5.13 | 0.12 | 9.3 | 29.1 | | 11.6 | | | 1.4 |
| RN (138Nkgha^-1^) | 5.11 | 0.12 | 7.8 | 29.9 | | 11.61 | | | 1.3 |
| 50% DBS (5.3tha^-1^) | 5.19 | 0.13 | 8.8 | 29.6 | | 11.59 | | | 1.4 |
| 100% DBS (10.6tha^-1^) | 5.18 | 0.13 | 9.6 | 29.7 | | 11.58 | | | 1.5 |
| 75%DBS(7.95tha^-1^) | 5.19 | 0.13 | 10.2 | 29.1 | | 11.62 | | | 1.5 |
| 75%DBS+25%N (7.95tha^-1^+34.5Nkgha^-1^) | 5.22 | 0.16 | 10.1 | 28.3 | | 11.57 | | | 1.9 |
| 50%DBS+50%N (5.3tha^-1^+69Nkgha^-1^) | 5.10 | 0.15 | 9.1 | 28.7 | | 11.59 | | | 1.7 |
| 25%DBS+75%N (2.65tha^-1^+103.5Nkgha^-1^) | 5.18 | 0.15 | 7.5 | 29.2 | | 11.61 | | | 1.7 |
| 100%DBS+25%N (10.6tha^-1^+34.5Nkgha^-1^) | 5.12 | 0.13 | 12.3 | 28.3 | | 11.62 | | | 1.5 |
| 100%DBS+50%N (10.6tha^-1^+69Nkgha^-1^) | 5.15 | 0.13 | 11.2 | 27.6 | | 11.60 | | | 1.5 |
| LSD | **NS** | **NS** | **NS** | **NS** | | **NS** | | | **NS** |
| CV% | **1.2** | **14.3** | **22.43** | **3.3** | | **0.21** | | | **14.19** |
| 2020-21 site1(Y2S1) | | | | | | | | | |
| Treatment | pH | TN% | Av P (ppm) | CEC (cmol kg^-1^) | | | C:N | | OC% |
| Control (0,0) | 5.2 | 0.15 | 4.4 | 27.3 | | | 11.59 | | 1.7 |
| RN (138Nkgha^-1^) | 5.3 | 0.16 | 4.3 | 27.7 | | | 11.59 | | 1.8 |
| 50% DBS (5.3tha^-1^) | 5.3 | 0.14 | 6.5 | 30.8 | | | 11.61 | | 1.7 |
| 100% DBS (10.6tha^-1^) | 5.4 | 0.15 | 6.2 | 27.4 | | | 11.60 | | 1.8 |
| 75%DBS(7.95tha^-1^) | 5.4 | 0.15 | 5.8 | 28.6 | | | 11.58 | | 1.7 |
| 75%DBS+25%N (7.95tha^-1^+34.5Nkgha^-1^) | 5.3 | 0.14 | 5.4 | 24.4 | | | 11.63 | | 1.7 |
| 50%DBS+50%N (5.3tha^-1^+69Nkgha^-1^) | 5.3 | 0.14 | 4.5 | 26.7 | | | 11.61 | | 1.6 |
| 25%DBS+75%N (2.65tha^-1^+103.5Nkgha^-1^) | 5.2 | 0.14 | 4.6 | 27.5 | | | 11.59 | | 1.7 |
| 100%DBS+25%N (10.6tha^-1^+34.5Nkgha^-1^) | 5.4 | 0.17 | 4.4 | 28.4 | | | 11.59 | | 1.9 |
| 100%DBS+50%N (10.6tha^-1^+69Nkgha^-1^) | 5.4 | 0.16 | 4.4 | 28.5 | | | 11.61 | | 1.9 |
| LSD | **NS** | **NS** | **NS** | **NS** | | | **NS** | | **NS** |
| CV% | **2.12** | **13.5** | **21.6** | **9.0** | | | **0.17** | | **13.5** |
| 2020-21 site2(Y2S2) | | | | | | | | | |
| Treatment | pH | TN% | Av P (ppm) | | CEC (cmol kg^-1^) | | | C:N | OC% |
| Control (0,0) | 5.3^cd^ | 0.17 | 12.2 | | 30.6 | | | 12.10 | 2.1 |
| RN (138Nkgha^-1^) | 5.4b^cd^ | 0.17 | 7.1 | | 31.9 | | | 11.60 | 1.9 |
| 50% DBS (5.3tha^-1^) | 5.5^abc^ | 0.18 | 11.5 | | 30.4 | | | 11.60 | 2.0 |
| 100% DBS (10.6tha^-1^) | 5.6^ab^ | 0.18 | 9.5 | | 32.1 | | | 12.62 | 2.3 |
| 75%DBS(7.95tha^-1^) | 5.6^ab^ | 0.17 | 13.2 | | 32.1 | | | 12.00 | 2.0 |
| 75%DBS+25%N (7.95tha^-1^+34.5Nkgha^-1^) | 5.5^abc^ | 0.18 | 13.3 | | 33.5 | | | 11.60 | 2.1 |
| 50%DBS+50%N (5.3tha^-1^+69Nkgha^-1^) | 5.5^abcd^ | 0.17 | 13.1 | | 32.6 | | | 12.01 | 2.1 |
| 25%DBS+75%N (2.65tha^-1^+103.5Nkgha^-1^) | 5.3^d^ | 0.16 | 9.5 | | 30.9 | | | 11.60 | 1.9 |
| 100%DBS+25%N (10.6tha^-1^+34.5Nkgha^-1^) | 5.6^a^ | 0.18 | 11.6 | | 31.8 | | | 11.80 | 2.1 |
| 100%DBS+50%N (10.6tha^-1^+69Nkgha^-1^) | 5.4^bcd^ | 0.17 | 15.3 | | 33.4 | | | 11.60 | 2.1 |
| LSD | **2.3** | **NS** | **NS** | | **NS** | | | **NS** | **NS** |
| CV% | **0.22** | **9.9** | **29.1** | | **6.1** | | | **5.9** | **13.8** |

Means followed by the same letter (s) within the column are not significantly different at (*P* ≤ 0.05). RNP=percent of recommended nitrogen and phosphorus, DBS =dry bio-slurry, pH= power of hydrogen concentration, TN% = total nitrogen percent, AvP = available phosphorus, OC% = organic carbon percent, C: N ratio = carbon to nitrogen ratio, CEC = cation exchange capacity, OM% = organic matter percent, Y1S1= year one site one, Y2S1= year two site one and Y2S2 = year two site two.

Table 4. The residual effect of dry bio-slurry on soil chemical properties after wheat harvesting in the potato-wheat cropping system

| **2020-21 site1(Y1S1)** | | | | | | | | |
| --- | --- | --- | --- | --- | --- | --- | --- | --- |
| Treatment | pH | TN% | Av P (ppm) | CEC (cmol kg^-1^) | C:N | | | OC% |
| Control (0,0) | 5.40 | 0.174bcd | 8.97 | 21.39 | 9.68 | | | 1.69 |
| RN (138Nkgha^-1^) | 5.40 | 0.196a | 10.12 | 21.69 | 10.01 | | | 1.95 |
| 50% DBS (5.3tha^-1^) | 5.40 | 0.197a | 9.25 | 26.13 | 9.64 | | | 1.88 |
| 100% DBS (10.6tha^-1^) | 5.38 | 0.168d | 11.04 | 23.51 | 11.81 | | | 1.98 |
| 75%DBS(7.95tha^-1^) | 5.40 | 0.192ab | 9.34 | 25.60 | 9.42 | | | 1.82 |
| 75%DBS+25%N (7.95tha^-1^+34.5Nkgha^-1^) | 5.42 | 0.172cd | 9.07 | 24.51 | 11.98 | | | 2.06 |
| 50%DBS+50%N (5.3tha^-1^+69Nkgha^-1^) | 5.36 | 0.188abc | 10.06 | 22.09 | 9.61 | | | 1.80 |
| 25%DBS+75%N (2.65tha^-1^+103.5Nkgha^-1^) | 5.35 | 0.202a | 10.08 | 25.11 | 10.14 | | | 2.04 |
| 100%DBS+25%N (10.6tha^-1^+34.5Nkgha^-1^) | 5.31 | 0.194ab | 10.46 | 21.81 | 11.01 | | | 2.13 |
| 100%DBS+50%N (10.6tha^-1^+69Nkgha^-1^) | 5.37 | 0.205a | 10/76 | 23.78 | 9.35 | | | 1.89 |
| **LSD** | NS | 0,020 | NS | NS | NS | | | NS |
| **CV%** | 1.3 | 6.2 | 12.7 | 11.9 | 14.8 | | | 13.3 |
| **2021-22 site1(Y2S1)** | | | | | | | | |
| Treatment | pH | TN% | Av P (ppm) | CEC (cmol kg^-1^) | | C:N | | OC% |
| Control (0,0) | 5.61 | 0.178 | 10.15 | 27.70d | | 7.61d | | 1.36b |
| RN (138Nkgha^-1^) | 5.53 | 0.179 | 10.22 | 30.52bc | | 10.31abc | | 1.85a |
| 50% DBS (5.3tha^-1^) | 5.69 | 0.180 | 11.56 | 34.67a | | 12.25abc | | 2.20a |
| 100% DBS (10.6tha^-1^) | 5.67 | 0.160 | 9.85 | 31.77bc | | 12.42ab | | 1.98a |
| 75%DBS(7.95tha^-1^) | 5.64 | 0.194 | 9.85 | 31.09bc | | 9.74cd | | 1.88a |
| 75%DBS+25%N (7.95tha^-1^+34.5Nkgha^-1^) | 5.70 | 0.178 | 11.76 | 29.56cd | | 10.79abc | | 1.90a |
| 50%DBS+50%N (5.3tha^-1^+69Nkgha^-1^) | 5.62 | 0.167 | 10.69 | 30.63bc | | 11.38abc | | 1.90a |
| 25%DBS+75%N (2.65tha^-1^+103.5Nkgha^-1^) | 5.54 | 0.186 | 9.59 | 30.37bc | | 10.00bcd | | 1.86a |
| 100%DBS+25%N (10.6tha^-1^+34.5Nkgha^-1^) | 5.58 | 0.172 | 9.88 | 31.99bc | | 12.82a | | 2.20a |
| 100%DBS+50%N (10.6tha^-1^+69Nkgha^-1^) | 5.44 | 0.185 | 10.19 | 32.91ab | | 11.78abc | | 2.17a |
| **LSD** | NS | NS | NS | 2.60 | | 2.61 | | 0.41 |
| **CV%** | 2.0 | 6.7 | 14.4 | 4.8 | | 14.1 | | 12.6 |
| **2021-22 site2(Y2S2)** | | | | | | | | |
| Treatment | pH | TN% | Av P (ppm) | CEC (cmol kg^-1^) | | | C:N | OC% |
| Control (0,0) | 5.51 | 0.176de | 10.03 | 29.43c | | | 12.03 | 2.12 |
| RN (138Nkgha^-1^) | 5.65 | 0.190bcd | 8.76 | 30.69bc | | | 12.19 | 2.29 |
| 50% DBS (5.3tha^-1^) | 5.62 | 0.186bcde | 13.49 | 35.92 a | | | 12.78 | 2.38 |
| 100% DBS (10.6tha^-1^) | 5.62 | 0.209ab | 14.08 | 33.68ab | | | 11.63 | 2.43 |
| 75%DBS(7.95tha^-1^) | 5.76 | 0.178cde | 11.74 | 34.47a | | | 13.41 | 2.39 |
| 75%DBS+25%N (7.95tha^-1^+34.5Nkgha^-1^) | 5.68 | 0.207ab | 12.93 | 34.81a | | | 12.29 | 2.54 |
| 50%DBS+50%N (5.3tha^-1^+69Nkgha^-1^) | 5.57 | 0.202abc | 13.08 | 35.88a | | | 11.47 | 2.30 |
| 25%DBS+75%N (2.65tha^-1^+103.5Nkgha^-1^) | 5.59 | 0.164e | 11.07 | 33.27ab | | | 13.52 | 2.19 |
| 100%DBS+25%N (10.6tha^-1^+34.5Nkgha^-1^) | 5.66 | 0.223a | 11.51 | 36.06a | | | 10.09 | 2.24 |
| 100%DBS+50%N (10.6tha^-1^+69Nkgha^-1^) | 5.66 | 0.207ab | 11.21 | 35.95a | | | 11.75 | 2.42 |
| **LSD** | NS | 0.025 | NS | 3.78 | | | NS | NS |
| **CV%** | 2.3 | 7.6 | 17.8 | 6.5 | | | 10.5 | 7.3 |

Means followed by the same letter (s) within the column are not significantly different at (*P* ≤ 0.05). RNP=percent of recommended nitrogen and phosphorus, DBS =dry bio-slurry, pH= power of hydrogen concentration, TN% = total nitrogen percent, AvP = available phosphorus, OC% = organic carbon percent, C: N ratio = carbon to nitrogen ratio, CEC = cation exchange capacity, OM% = organic matter percent, Y1S1= year one site one, Y2S1= year two site one and Y2S2 = year two site two.

**3.2** **Main and residual effects of dry bio slurry on potato and wheat yield and yield components in the potato-wheat cropping system**

**3.2.1 Plant height and number of Stem per hill of potato**

Combined analysis of dry bio slurry with N rates was significantly (P < 0.05) affects plant height (Table 3). Numerically,the highest values of plant height (52.1 and 48.8cm) was achieved with addition of RNP and 25%DBS with 75%N recpectively as compare control and 50%DBS that gives minimum values (Table 3).Increasing of plant height in response to application of DBS with N fertilizer may be due to the improvement of physico- chemical properties of the soil that resulted increased water absorption and nutrient utilization of the plant.Moreover, application of DBS may deliver balanced micro and macronutrients as well as enhanced availability of plant nutrients,which would help to enhance the metabolic activity of microorganisms and improvement of plant growth.The result in agreement with the findings of Melkamu *et al.,* (2020) who observed that longer plants when potato plants were applied with farm yared manure (13.5tha^-1^) and NPS (245.1kgha^-1^).lt is also harmonized with the findings of moniruzzaman *et al*.(2009) who recorded maximum plant height of french bean from the application of 120 kg N ha^-1^ while, the minimum value was recorded from the control treatment. Another study that was conducted by Muhammad *et al*. (2017) reflected that the highest value of mung bean plant height (78.08 cm) was recorded from the treatment which received 20:50 NP kg ha^-1^ with inoculation Rhizobium as compared to the lowest value 68 cm on control treatment. On the other hand, a combined analysis of variance revealed that DBS with N fertilizers had not significant effects on the stem numbers of potato per hill (Table3). This may be due to the parameter more favors genetic makeup, physiological age and tuber seed size rather than a nutrient supplement. This line with finding of De La, Guillen, & Del Moral (1994) shoot number of potato is mostly determined by the genetic makeup, the physiological age, and the size of potato seed tubers rather than mineral nutrients added in the form of fertilize

**3.2.2 Plant height and spike length of wheat**

Over year residual effect analysis of dry bio-slurry significantly affects at (P < 0.05) both plant height and spike length (Table 5) The highest values of plant height and spike (93.2 and 9.3 cm) were achieved By application of 100% DBS as compared to untreated plot (Table 5). This might be from the positive effect of DBS for delivering balanced micro and macronutrients as well as enhanced availability of plant nutrients via improving of soil properties. The result agreed with the findings of Balasubramanian.,et al 2016 who observed that the longer plants in plots which are received75%cow dung with 25 %vermicompost than non-treated plots .lt is also harmonized with Bilkis *et al*.,2017 who recorded maximum plant height(101.5cm) of Boro rice from the application of 5tha-1 trichocompost while the minimum value(78.6) was recorded from control treatment .The result related to spike length also much with Pandey.,et al 2020 who said that an application of 15tha^-1^ bio-gas slurry gives the highest spike length of wheat than checked treatment or control

Table 5. Main and residual effects of dry bio-slurry with equivalence N on growth parameters of potato and wheat

| Treatments | Main effect of DBS on Potato | | Residual effect DBS on Wheat | |
| --- | --- | --- | --- | --- |
|  | **PH (cm)** | **NSPH** | **PH(cm)** | **SL(cm)** |
| Control (0,0) | 29.4^de^ | 3.3 | 68.2d | 6.7d |
| RN (138Nkgha^-1^) | 52.1^a^ | 4.8 | 89.1c | 8.5c |
| 50% DBS (5.3tha^-1^) | 28.2^e^ | 4.4 | 92.3ab | 8.7bc |
| 100% DBS (10.6tha^-1^) | 30.4^de^ | 4.4 | 93.2a | 9.3a |
| 75%DBS(7.95tha^-1^) | 36.6^cd^ | 4.5 | 93.0ab | 9.0abc |
| 75%DBS+25%N (7.95tha^-1^+34.5Nkgha^-1^) | 41.9^bc^ | 4.6 | 92.1abc | 9.0abc |
| 50%DBS+50%N (5.3tha^-1^+69Nkgha^-1^) | 42.7^bc^ | 3.7 | 90.2bc | 8.9abc |
| 25%DBS+75%N (2.65tha^-1^+103.5Nkgha^-1^) | 48.8^ab^ | 4.0 | 90.2bc | 8.7bc |
| 100%DBS+25%N (10.6tha^-1^+34.5Nkgha^-1^) | 44.0^bc^ | 4.3 | 92.1ab | 9.0abc |
| 100%DBS+50%N (10.6tha^-1^+69Nkgha^-1^) | 47.1^ab^ | 4.2 | 90.6abc | 9.1ab |
| LSD | **7.9** | **NS** | 3.0 | 0.5 |
| CV% | **20.9** | **30.1** | 3.6 | 6.6 |

Means followed by the same letter (s) within the column are not significantly different at (*P* ≤ 0.05). DBS= dry bio slurry, N= nitrogen, PH= plant height, NSPH = number of stems per hill, SL=spike length.

### 3.2.3 Number of tubers per plant and total tuber yield of potato

The combined analysis result in both years across sites indicates that yield and yield a component of potato was significantly differ at (P<0.05) due to the effects of DBS with equivalence N (Table 6). The application of 75% N with 25% DBS gives the highest fresh total tuber yield (27.6tha^-1^) while the lowest fresh tuber yield (8.6tha^-1^) was observed at control. This might be due to the release of N from dry bio-slurry (DBS) and Urea to soil solution that makes for plant better growth and development. Moreover, it could be due to the addition of both macro and micro nutrients from the dry bio-slurry (DBS) by improving of soil physico-chemical properties. This study is in line with the findings of Yalemtsehay and Fisseha (2016) who revealed that, the supplying of recommended inorganic fertilizer (100kg DAP, 50kg Urea and 50kg Murate potash per hectare) with 8tha^-1^bio-slurry gives maximum (266.7 t ha^-1^) yield of cabbage as compare to the lowest (160 t ha^-1^) from the control treatment and it gives about 66.7% yield increment due to the combination both bio-slurry and recommended fertilizers over control. On the other hand the study done by Tsegaye *et al*.(2020) revealed that the lowest value of fresh shoot biomass and marketable yield of potato tuber were achieved from control while the highest values were obtained in plots that treated combined application of farm yard manure and recommended nitrogen and phosphorus.

Similarly number of tubers per plant (NTPP) was significantly affected at (P<0.05) by the application DBS with equi-valence nitrogen. Maximum value of NTPP was observed through the application of recommended NP as compare to control treatment (Table 6).Even if the maximum value occurs at Recommended NP the other dry bio slurry-nitrogen combination treatments also give better yield advantage than control. This might be due to; the harmonization of organic and inorganic fertilizers for uptake and assimilation of nutrients to potato tubers by increasing the availability of native soil nutrients through higher biological activities. The result coincides with Suh *et al*. (2015) who observed that an application of combined organic and inorganic fertilizers increasing number of tubers per plant in treated plots than control or untreated plots. Another study done by Geremew *et al*. (2019) showed that an addition of dry bio-slurry with recommended nitrogen phosphorus increase 40 to 73% number of fruits per plant on tomato.

**3.2.4 Grain yield and aboveground biomass of wheat**

Yield and aboveground biomass of wheat was significantly differ at (P<0.05) (Table 6) by residual effect of dry bio slurry. The application of 100%DBS gives the highest grain yield (3.85tha^-1^) as compare to control. Similarly, addition of 75%DBS gives the highest aboveground biomass yield of wheat (9.59tha^-1^) as compare to control (Table 6). Releasing N from dry bio-slurry (DBS) to soil solution may makes for plant better growth and development. Moreover, it could be due to the addition of both macro and micro nutrients from the dry bio-slurry in to soil solution as residual time friend. Beyond its positive effect on soil improvement by increasing the availability of native soil nutrients. The result coincides with Pandey *et al*., 2020 who reported the supplement of 10 and15 t ha^-1^ increasing grain yield of wheat significantly by scored maximum yield than control. Similarly study done by Shahid *et al*., 2016 revealed that he application of combined use of biogas slurry and chemical fertilizer @ 50% has a good strategy for sustainable crop yield by improving soil health. Beside to this Mercy *et al*. (2022) also showed that an addition of 100% dry bio-slurry increase Stover and stalk yield of maize by 45.5 and 42.2% than control treatments through biological activities

Table6.Response of potato and wheat yield parameters for dry bio slurry and equivalence nitrogen main and residual effect

| Treatments | Main effect of DBS on Potato | | Residual effect DBS on Wheat | | |
| --- | --- | --- | --- | --- | --- |
|  | NTPP | TYD t ha^-1^ | GY(t ha^-1^) | BY(tha^-1^) | 1000 SW(g) |
| Control (0,0) | 4.2^e^ | 8.6^d^ | 1.23d | 3.11d | 30.1 |
| RN (138Nkgha^-1^) | 10.9^a^ | 26.2^ab^ | 3.17c | 7.9c | 32.4 |
| 50% DBS (5.3tha^-1^) | 5.0^de^ | 12.0^cd^ | 3.33bc | 8.37bc | 32.8 |
| 100% DBS (10.6tha^-1^) | 6.4^cd^ | 13.4^c^ | 3.85a | 9.52ab | 33.6 |
| 75%DBS(7.95tha^-1^) | 7.3^bc^ | 14.9^c^ | 3.83a | 9.59a | 33.9 |
| 75%DBS+25%N (7.95tha^-1^+34.5Nkgha^-1^) | 6.5^bcd^ | 23.0^b^ | 3.47abc | 9.08ab | 33.2 |
| 50%DBS+50%N (5.3tha^-1^+69Nkgha^-1^) | 6.4^cd^ | 24.6^ab^ | 3.47abc | 8.53abc | 32.1 |
| 25%DBS+75%N (2.65tha^-1^+103.5Nkgha^-1^) | 6.8^bc^ | 27.6^a^ | 3.28bc | 8.43abc | 32.6 |
| 100%DBS+25%N (10.6tha^-1^+34.5Nkgha^-1^) | 7.0^bc^ | 25.2^ab^ | 3.70ab | 9.56a | 33.0 |
| 100%DBS+50%N (10.6tha^-1^+69Nkgha^-1^) | 8.1^b^ | 26.8^a^ | 3.59abc | 9.06abc | 32.4 |
| LSD | 1.7 | 3.7 | 0.5 | 1.2 | NS |
| CV% | 25.6 | 19.4 | 15.6 | 14.8 | 10.1 |

## Means followed by the same letter (s) within the column are not significantly different at (P ≤ 0.05). DBS= dry bio slurry, N= nitrogen, PH= plant height, NTPP = number of tubers per plant, TYD, = total tuber yield, GY= grain yield, BY= above ground biomass and SW = seed weight.

## 3.3 Partial Budget Analysis

Net benefits were calculated by current fertilizer (Urea) cost of 13.643 Birr kg^-1^, cost of DBS kg^-1^ 0.2Birr, field price of potato was 6 Birr kg^-1^, cost of labor per man day in the area is 70 Birr. The marginal rate of returns 100% was used to determine the acceptability of treatments. This economic analysis indicated that most of treatments give high net benefit than control (Table 7). Addition of 50%DBS with 50%RN and 25%DBS with 75%RN gave (128613.6 birr) and (144460.3 birr) net benefit with marginal rate of return of 8461.1% and 4486.3% respectively . This indicate that for every 1birr invested for 50%DBS with 50%RN and 25%DBS with 75%RN in field, farmers can obtain an additional 84.611 and 44.863 Birr respectively (CIMMYT, 1988). All Un-dominated treatment rates could be acceptable for potato producers in the study area except the dominated one’s. Even if enough un-dominated alternatives treatments have been available as choice for potato cultivation farmers in the study area. Application of 25%DBS with75%RN and 50%DBS and 50%N scored the most promising result 144460.3 and 128613.6 Birr net benefit respectively as compare to other treatments (Table 7). Therefore, the most economical rate for producers with low cost and higher benefit was 25%DBS with 75%RN.As second option farmers can used 50%DBS with 50%RN for potato production because of it also have promising net benefit with marginal rate of return

Table 7. Partial budget and marginal analysis of potato as affected by the application of dry bio-slurry with nitrogen at Yilemana Densa District.

| Treatments  (RN +DBS kgha^-1^) | 10% Adjusted tuber Yield t ha^-1^ | Total variable Cost Birr ha^-1^ | Net Benefits Birr ha^-1^ | MRR% |
| --- | --- | --- | --- | --- |
| Control | 7.74 | 0 | 46440 | 0 |
| 50% DBS | 10.8 | 1760 | 63040 | 943.2 |
| 75%DBS | 13.41 | 2640 | 77820 | 1679.6 |
| 100% DBS | 12.06 | 3520 | 68840 | D |
| 50%DBS+50%N | 22.14 | 4226.45 | 128613.6 | 8461.1 |
| 25%DBS+75%N | 24.84 | 4579.675 | 144460.3 | 4486.3 |
| 100%DBS+25%N | 22.68 | 4753.225 | 131326.8 | D |
| RNP | 23.58 | 4932.9 | 136547.1 | D |
| 100%DBS+50%N | 24.12 | 5986.45 | 138733.6 | D |
| 75%DBS+25%N | 20.7 | 10873.23 | 113326.8 | D |

RNP=percent of recommended nitrogen and phosphorus in kg per hectare, DBS=dry bio-slurry in kilogram per hectare, MRR= marginal rate of return; D= is dominated treatments

**4. Conclusion and Recommendation**

Yield and yield components of potato and wheat were significantly affected by main and residual effect of dry bio slurry with nitrogen supplement. Both Potato and wheat grown on by addition of dry bio-slurry with nitrogen was improved in yield and yield components as compared to control. Applied dry bio-slurry with N (nitrogen) had a positive impacts on plant height, number of tubers per plant, total tuber yield, grain yield and above ground biomass. Supplement of Dry bio-slurry with N rate in most treatments gives higher net benefit when compared to control. Among treatments used for this experiment undominated once gave better net benefit response with over 100% marginal rate of return than control in the main crop (potato) and residual crop (wheat). Therefore, resource poor producers or small scale farmers can benefited, if they apply these soil improvement rates depending on their convenience. Related to this application 50%DBS with 50% N gives a higher net benefit with 8461.1% marginal return. As a result, small-scale farmers could use it for potato-wheat production system in the area. In a mixed farming system like the Yilemana Densa district livestock production is a component of their livelihood; cattle manure and biodigester plantation is available. Hence, farmers should be encouraged to practice these fertilizers in order to produce vegetables and cereal crops in their backyards and nearest farms. Finally, similar studies will be done in different locations, crops, years and forms of bio-slurry in order to confirm the current findings and to give a concrete recommendation for crop production and soil health amendment in the study area.

**Conflict of interest**

There is no conflict of interest between Authors

**Acknowledgment**
The author is grateful to Adet Agricultural research center for its administrative and budget support

**Reference**

A. Balasubramanian, P. Stalin, M.Saravanaperumal and S.R.Vinoth kumar.2016. Residual effect of Integrated Nutrient Management practices on growth and yield of rice fallow blackgram (Vigna mugno L.). Journal of Emerging Technologies and Innovative Research, 3(5): 2349-5162.

Alva, A. 2004. Potato nitrogen management. J. Veg. Crop Prod., 10: 97-130.

B. P. Pandey*, N. Khatri, M. Yadav, K. R. Pant, R. P. Poudel, and A. H. Khan.2020. Effect of Digestate /Biogas Slurry In Wheat Under Rice – Wheat Cropping System. Journal of Agriculture and Forestry University, 4: 67-75.

Chanie, C., Teshome, T., Temesgen, T., & Berihun, B. (2017). Characterization of potato production, marketing, and utilization in North Western Amhara Region, Ethiopia. Journal of Horticulture and Forestry, 9(3), 17–25. doi:10.5897/JHF

CIMMYT. 1988. From Agronomic Data to Farmer’s Recommendation: an Economic Training Manual. Revised Edition. Mexico, D.F.

CSA (Central Statistic Agency). 2016. Agricultural sample survey report on area and production of major crops for the period 2015/2016 cropping season. Vol I. Statistical bulletin 584, Addis Ababa.

CSA (Central Statistic Agency).2014.Agricultural sample survey report on area and production of major crops for the period 2013/2014 cropping season. Vol I. Statistical Bulletin 532, Addis Ababa.

CSA (Central Statistical Agency). (2018). Agricultural sample survey: Area and production of major crops. Addis Ababa, Ethiopia.

CSA (Central Statistical Agency). (2020). Agricultural sample survey 2019/20 (2012 E.C) volume I. Report on area and production of major crops meher season crops for private peasant holdings. CSA, Addis Ababa.

De La, I., Guillen, I. A., & Del Moral, L. F. G. 1994. Yield development in potatoes as influenced by cultivar and the timing and level of nitrogen fertilization. American Potato Journal, 71, 165–173. Doi: 10.1007/ BF02849051

Efrem B, Hirut K, Getachew B. 2000. Durum wheat in Ethiopia: an old crop in an ancient land. Addis Ababa: Institute of Biodiversity Conservation and Research.

Errebhi, M., Rosen, C.J., Gupta, S.C. and Birong, D.E. 1998. Potato Yield Response and Nitrate Leaching as Influenced by Nitrogen Management. Agron. J. 90:10–15.

Eyasu Elias .2016. Soils of the Ethiopian highlands: Geomorphology and Properties. CASCAPE Project, ALTERA, Wageningen University and Research Centre (Wageningen UR), the Netherlands, p. 385

Eyasu Elias. 2016. Soils of the Ethiopian highlands: Geomorphology and Properties. CASCAPE Project, ALTERA, Wageningen University and Research Centre (Wageningen UR), the Netherlands, p. 385.

FAOSTAT. (2017). Food and Agriculture Organization corporate statistical database of the United Nations. Retrieved from http://www.fao.org/faostat/en/#data/QC

FAOSTAT. 2015. Online http://faostat.fao.org/site/291/default.aspx. Published 2014. Accessed Mar 6.

Geremew Biramo , Girma Abera and Birhanu Biazin.2019. Effects of dry bioslurry and chemical fertilizers on tomato growth performance, fruit yield, and soil properties under irrigated conditions in Southern Ethiopia.

Gomez K.A and A.A. Gomez. 1984. Statistical procedures for agricultural research, 2nd edition. John Wiley and Sons, New York. International Congress Series 1291: 42-52.

Karki KB (1997). Estimation of plant nutrient loss from biogas slurry. Biogas Support Programme.

Khan SA, Malav LC, Kumar S, Malav MK and Gupta N (2015). Resource utilization of biogas slurry for better yield and nutritional quality of baby corn. Advances in Environmental and Agricultural Science, 382-394.

Landon J.R. 1991. Booker tropical soil manual: a handbook for soil survey and agricultural land evaluation in the tropics and subtropics. New York.

M. A. Haque, M. Jahiruddin, M. S. Islam, M. M. Rahman, M. A. Saleque. (2018). Effect of Bio slurry on the Yield of Wheat and Rice in the Wheat– Rice Cropping System. Journal of Agric Res, 7(4):432–442.

Melkamu Alemayehu, Minwyelet Jemberie, Tadele Yeshiwas & Masho Aklile.2020. Integrated application of compound NPS fertilizer and farmyard manure for economical production

Mercy Kamau Rewe, Esther Muindi, James Ndiso, Kevin Kinusu, Stephen Mailu,Peterson Njeru and Rewe Thomas.2022. Integrated effect of liquid bio slurry and inorganic fertilizer on selected soil chemical properties, maize (Zea mays) growth, yield and grain quality. African Journal of Crop Science, 10 (5): 001-016.

Moniruzzaman M, Halim G.M.A and Firoz Z A. 2009. Performances of the French bean as influenced by plant density and nitrogen application. Bangladesh. JAR.34(1): 105-111.

Muhammad U.C, Muhammad U.H, Imran K.2017. Effect of different nitrogen and phosphorus fertilizer levels in combination with nitrogen and phosphorus solubilizing inoculants on the growth and yield of mung bean. PJLSS. 15(1): 31-36.

Olsen S.R, Cole C.V, Watanabe F.S and Dean L.A.1954. Estimation of available phosphorus in soils by extraction with sodium carbonate. USDA Circular, 939: 1-19.

Rhaman M.S., Kibria M.G., Hossain M., and Hoque M.A. 2016. Effects of organic manure and bio-slurries with chemical fertilizers on growth and yield of rice (cv. BRRI dhan28). Int. J. Expt. Agric., 6 (3): 36-42

S. Bilkis, M.R. Islam, M. Jahiruddin, M.M. Rahaman. 2017. Integrated Use Of Manure And Fertilizers Increases Rice Yield, Nutrient Uptake And Soil Fertility In The Boro-Fallow-T.Aman Rice Cropping Pattern. SAARC J. Agri, 15(2): 147-161.

Sahlemedihn Sertus and Taye Bekel. 2000. Procedures for sol and plant analysis. Addis Ababa, Ethiopia.

Sandeep Sharma & Salwinder Singh Dhaliwal.2019. Effect of Sewage Sludge and Rice Straw Compost on Yield, Micronutrient Availability and Soil Quality under Rice–Wheat System. Journal Communications In Soil Science and Plant Analysis.

Shahid, M., A. K. Shukla, P. Bhattacharyya, R. Tripathi, S. Mohanty, A. Kumar, B. Lal, P. Gautam, R. Raja, B. B. Panda., et al. 2016. Micronutrients (Fe, Mn, Zn and Cu) balance under long-term application of fertilizer and manure in a tropical rice–rice system. Journal of Soils and Sediments 16 (3):737–47. doi: 10.1007/s11368-015- 1272-6.

Shahzad, K., A. Khan, J.U. Smith, M. Saeed, S.A. Khan and S.M. Khan. 2015. Residual effects of different tillage systems, bio slurry and poultry manure on soil properties and subsequent wheat productivity under humid subtropical conditions of Pakistan. International journal Biosci. 6(11): 99-108.

Shankarappa TH. Gurumurthy SB, Patil SV and Lokesh MS. (2012). Influence of phosphorus enriched biogas spent slurry (BSS) on growth and yield of sunflower (Helianthus annuus). Journal of Plant Science, 7: 253-258.

Tamado T, Mitiku W. 2017. Effect of combined application of organic and mineral nitrogen and phosphorus fertilizer on soil physico-chemical properties and grain yield of food Barley (Hordeum vulgare L.) in Kaffa Zone, South-western Ethiopia. MEJS. 9(2):242–261.

Tekalign Tadese. 1991. Soil, plant, water, fertilizer, animal manure and compost analysis. Working Document No. 13. International Livestock Research Center for Africa, Addis Ababa.

Tsegaye Girma,Birhanu Biazin, Sheleme & Berga Lemaga.2020. Integrated Application of Organic and Blended Mineral Fertilizers Improves Potato Productivity and Income for Smallholder Farmers in Acidic Soils.

Tsegaye Terefe, Tewodros Ayalew and Hussien Mohammed Beshir.2018. Combined Application of Bioslurry and Inorganic Fertilizers on Quality Traits of Cabbage and Soil Properties. Asian Journal of Biological Sciences, 11 (1): 24-32

White, P.J., Wheatley, R.E., Hammond, J.P. and Zhang, K. 2007. Minerals, soils and roots. In: Vreugdenhil D (ed) Potato biology and biotechnology, advances and perspectives. Elsevier, Amsterdam, pp: 739-752.

Workineh Ejigu.2020. Integrated fertilizer application improves soil properties and maize (Zea mays L.) yield on Nitisols in Northwestern Ethiopia.

Yalemtsehay Debebe and Fisseha Itana.2016. Comparative study on the effect of applying biogas slurry and inorganic fertilizer on soil properties, growth and yield of White Cabbage (Brassica oleracea var. capitata f. alba). Journal of Biology, Agriculture and Healthcare. 6(19).

Zelalem Addis Musse, Tarekegn Yoseph Samago & Hussien Mohammed Bisher.2020. Effect of liquid bio-slurry and nitrogen rates on soil physico-chemical properties and quality of green bean (Phaseolus vulgaris L.) at Hawassa Southern Ethiopia.Journal of plant interaction, 15 (1):207–212.

Zelalem, A., Tekalign, T. and Nigussie, D. 2009. Response of potato (Solanum tuberosum L.) to different rates of nitrogen and phosphorus fertilization on vertisols at DebreBerhan, in the central highlands of Ethiopia. African journal of plant science, 3: 16-24.an journal of plant science, 3: 16-24.
