# Supplementary material for: Effects of dry bio-slurry and nitrogen fertilizer on potato and wheat yields under rotation cropping system
Source: PLoS One. 2024 Jul 31;19(7):e0306625. doi: 10.1371/journal.pone.0306625 (PMC11290689; doi:10.1371/journal.pone.0306625)
Supplement: Attachment — Submitted filename: Review report PONE-D-23-10312.docx [file pone.0306625.s002.docx]

**Review report**

**Manuscript number**: PONE-D-23-10312

**Title:** Main and residual effect of dry bio-slurry with nitrogen fertilizer application on soil properties and crop production under Potato-wheat cropping system

**General comment**

Integrated application of organic and inorganic fertilizers is an important soil amendment strategy that improves the sustainability of crop production including potato and wheat. Potato and wheat are produced by smallholder farmers in Ethiopia where enhancing their productivity contributes a lot for the livelihood improvement and ensuring food security in the country. In this regard, this research work may have significant contribution.

However, the manuscript is not well structured and lacks justification and objectives. The methodology is not well described and the results are not well presented and discussed. The authors are advised to make use of Microsoft grammar and spelling check. Comments to the individual section of the manuscript are presented below.

Title: It is suggested to rephrase as “Effects of dry-bio-slurry supplemented with chemical nitrogen fertilizer on growth and yield of potato and wheat under Potato-wheat cropping system

**Abstract**

- It is recommended to include problem statement and objective of the study at the beginning of the abstract.
- The conclusion should be changed after correcting the MRR analysis in the results and discussion.

**Introduction**

- The authors should justify why intergraded application of organic and inorganic fertilizer is better than the solo application of the fertilizers.
- Previous research experiences in the integrated application of fertilizers should be reviewed.
- Most of the information/references are very old and the authors should change them with recent references
- There are sentences that are difficult to understand and unfinished, which should be revised
- A lot of grammatical and typographical errors that should be revised
- For more information please refer the commented document

**Methodology**

This part of the manuscript should be given due attention and requires rigorous revision. Description of the study area, the production system and data collection methods implemented (growth and yield) should be clearly described so that it is understandable and could be repeated by other person (for more information, please refer the attached document).

**Results and discussion**

The way results are presented is not attractive for the reader. It is suggested to present first the results and then forward why it could be and then compare the results with others.

Results should be presented in the past tense form.

Although no description of site 1 and site 2 in the methodology, results of site 1 and site 2 (soil sample results) are presented, which are confusing.

The economic analysis should be done once again as the MRR of **50%DBS+50%N (**8461.1%) is not correct. Accordingly, the conclusion and recommendation part should be revised based on the corrected MRR value.

For more information, refer commented document

**Conclusion and recommendation**

It should be revised after the revision of the MRR values.

**References**

The way reference listed should be uniform and meet the guideline of the journal.
