# Supplementary material for: Effects of dry bio-slurry and nitrogen fertilizer on potato and wheat yields under rotation cropping system
Source: PLoS One. 2024 Jul 31;19(7):e0306625. doi: 10.1371/journal.pone.0306625 (PMC11290689; doi:10.1371/journal.pone.0306625)
Supplement: Attachment — Submitted filename: Response to Reviwers plose.docx [file pone.0306625.s004.docx]

Response to Authors

Dear respected editorial we have tried to adjust the manuscript by tracking, coloring based on your comments and without tracking

And here we also put the general response to the author’s comment

| **Comments** | **Response** |
| --- | --- |
| Reviewer 1  .title: It is suggested to rephrase as “Effects of dry-bio-slurry supplemented with chemical nitrogen fertilizer on growth and yield of potato and wheat under Potato-wheat cropping system  Abstract  • It is recommended to include problem statement and objective of the study at the beginning of the abstract.  • The conclusion should be changed after correcting the MRR analysis in the results and discussion.  Introduction  • The authors should justify why intergraded application of organic and inorganic fertilizer is better than the solo application of the fertilizers.  • Previous research experiences in the integrated application of fertilizers should be reviewed.  • Most of the information/references are very old and the authors should change them with recent references  • There are sentences that are difficult to understand and unfinished, which should be revised  • A lot of grammatical and typographical errors that should be revised  • For more information please refer the commented document | - We have are adjust the title based on reviewer **“Effects of dry-bio-slurry supplemented with chemical nitrogen fertilizer on growth and yield of potato and wheat under Potato-wheat cropping system** - Here we also incorporate statement of the problem at the begging of abstract - we have tried to check MRR by back tracing of the data its correct but our recommendation and conclusion adjusted from 50%RN+ 50DBS to 25%DBS +75%RN - we have justified it in the manuscript based on the comment - in case of reference we have tried to explorer recent works and adjust it based on the comment - we also tried adjust the language errors by the application of Grammerly |
| Methodology  This part of the manuscript should be given due attention and requires rigorous revision. Description of the study area, the production system and data collection methods implemented (growth and yield) should be clearly described so that it is understandable and could be repeated by other person (for more information, please refer the attached document). | We are tried to more clarify based on the comment |
| Results and discussion  The way results are presented is not attractive for the reader. It is suggested to present first the results and then forward why it could be and then compare the results with others.  Results should be presented in the past tense form.  Although no description of site 1 and site 2 in the methodology, results of site 1 and site 2 (soil sample results) are presented, which are confusing.  The economic analysis should be done once again as the MRR of 50%DBS+50%N (8461.1%) is not correct. Accordingly, the conclusion and recommendation part should be revised based on the corrected MRR value.  For more information, refer commented document  Conclusion and recommendation  It should be revised after the revision of the MRR values. | In this we make it in main body of the manuscript through tracking |
| References  The way reference listed should be uniform and meet the guideline of the journal. | Based on journal guide line we tried to adjust it |
| eviewer #2: I must appreciate authors for nice work in which they have tried to understand supplementation of N with DBS on the performance of Potato-wheat cropping system and soil quality. The study has been conducted by following established procedures and methods and is scientifically sound. The data recorded and presented in the MS are also in order. I have thoroughly gone through the MS and realized that there is still scope to further improve its quality, presentation and content. According some suggestions/comments have been tendered on the MS itself. These comments are easy to understand and self explanatory. In addition to these comments, some specific comments are as under. Authors are advised to revise the MS by duly incorporating all the suggestions/comments.  1. English language polishing is highly needed to bring the information of the MS in presentable form.  2. Authors are advised to include more yield attributes of both the crops for better and clear understanding of treatment effects on test crops.  In addition to the above, it is recommended to include system equivalent yield as study has been attempted in system mode.  3. Include recent and updated works to support findings. Authors are also advised to cross check the work cited in text and presented in the reference section and vice versa.  Reviewer #3: The research holds importance in the area where its being conducted. Although the program is well planned but it needs to be re written. The introduction is very weak, does not fully justify the topic. It needs to be revised keeping in view all the important parameters in the title. The results too are poorly written and discussed with most of the incomplete sentences.  the abstract also needs to be rewritten.. | •we also tried adjust the language errors by the application of Grammerly and for the sake of soil we only interested the listed yield parameters rather to other attributers .because of that we only use that parameters for those crops  .Yes we check it and adjust based on yours comment for all reference |
| Reviewer #4: Starting from the topic need major and careful modifications. The abstract does not contain the mail elements and it is poor. The result section is not properly interpreted. conclusion and recommendation section are poor. Majority of the references are very old. | .We have already adjust it based on reviewer 1 comment |
| 8. Please extract some data from table and present in good-quality figures. figures must be >300 dpi. | .Here We have think it’s better put it in table rather tables |
