# Supplementary material for: Effects of dry bio-slurry and nitrogen fertilizer on potato and wheat yields under rotation cropping system
Source: PLoS One. 2024 Jul 31;19(7):e0306625. doi: 10.1371/journal.pone.0306625 (PMC11290689; doi:10.1371/journal.pone.0306625)
Supplement: Attachment — Submitted filename: Response to Reviwers plose best.docx [file pone.0306625.s009.docx]

**Response to Authors**

Dear respected editorial we have tried to adjust the manuscript by tracking, coloring based on your comments and without tracking

And here we also put the general response to the author’s comment

| **Comments** | **Response** |
| --- | --- |
| **Reviewer 1** | **Authors** |
| I. The title shall be revised by considering the replacement/substitution of chemical N with DBS II. Statement of the problem in the abstract requires more articulation, III. Please review previous research findings/practices that showed the improvement of growth and yield of crops and soil properties through application of organic and inorganic fertilizers (only general principles are reviewed). IV. Correct the net plot area and MRR, which create deviations in the collected data and conclusion and recommendation V. The recommendation in the abstract and in conclusion is different. Please revise and make a similar recommendation in both sections of the manuscript. VI. Revise grammatical and editorial errors throughout the document | I. We have been adjusting the title based on reviewer 4 suggestion **“Effects of dry bio slurry with nitrogen fertilizer on yields of potato and wheat under short term crop rotation system** by considering the comment of reviewer 1 II. Here we are done the re -articulation of for statement of the problem in the abstract part based on the reviewer comment III. Based on the comment we in corporate the previous findings that deals the integration effect of organic and inorganic fertilizers on growth and soil properties IV. Here we correct both MRR and net plot area-based reviewer comment V. We have been making it similar in both parts of the manuscript for recommendation VI. Here we want to appreciate all of the reviewers including reviewer1 for their effort as manuscript be well edited by language writing. by fully considering and give attention for there frequent comment we have correct and check it by fluent speaker and language skilled persons in addition to our correction effort .so the manuscript well edited and corrected in terms of language |
| **Comments** | **Response** |
| **Reviewer 4** | **Authors** |
| I. Suggested title: Effects of dry bioslurry with nitrogen fertilizer on yields of potato and wheat under short term crop rotation system II. The abstract lacks short background of the problem. III. The result section in the abstract is not well written. Better to summarize the key results only. IV. There are too long sentences in the paper (see the first four lines of the abstract, description of study area), difficult to realize the concept. V. The language is generally poor. It should be further improved by subject experts. VI. There are editorial errors like spacing (2.2.2Gudenie potato variety, 2.2.3TAY variety of Wheat), spelling (List significant difference (LSD). VII. SAS software version 9.4 is not available during 2002. You have cited the correct releasing year. VIII. In table 2, describe the clay, sand and silt contents and remove repeated rows containing names of soil properties. | I. We have been adjusting the title based on reviewer suggestion “**Effects of dry bio slurry with nitrogen fertilizer on yields of potato and wheat under short term crop rotation system** II. we incorporate the background of the problem in abstract part based on this comment (reviewer 4). III. we are correct it in both parts by summarize. IV. we make clear and short in most lines and sentences in the manuscript. V. Here we want to appreciate all of the reviewers including reviewer4 for their effort as manuscript be well edited by language writing. by fully considering and give attention for their frequent comment we have correct and check it by fluent speaker and language skilled persons in addition to our correction effort .so the manuscript well edited and corrected in terms of language. VI. We correct it by 2014.   VIII. we also correct it based on the comment |
