# Supplementary material for: Effects of dry bio-slurry and nitrogen fertilizer on potato and wheat yields under rotation cropping system
Source: PLoS One. 2024 Jul 31;19(7):e0306625. doi: 10.1371/journal.pone.0306625 (PMC11290689; doi:10.1371/journal.pone.0306625)
Supplement: Attachment — Submitted filename: Review report PONE-D-23-10312R3.docx [file pone.0306625.s011.docx]

**Review report**

**Manuscript number**: PONE-D-23-10312R3

**Title:** Effects of dry-bio-slurry with chemical nitrogen fertilizer on growth and yield of potato and wheat under short term crop rotation

**General comment**

The authors addressed most of the comments raised in the previous version. However, I am not still satisfied with the introductory sentences in the abstract, which I tried to rephrase it in the document. Moreover, I request the authors as well as the editors to revisit the document towards grammar, sentence construction and typographical errors. It is one of the major problems of the document. In addition, the MRR values are not correctly calculated, which I also tried to correct in the document.

I recommend accepting the document after incorporation of the comments and rigorous grammatical and typographical corrections. For more information, please refer the commented document.
