# Supplementary material for: Effects of dry bio-slurry and nitrogen fertilizer on potato and wheat yields under rotation cropping system
Source: PLoS One. 2024 Jul 31;19(7):e0306625. doi: 10.1371/journal.pone.0306625 (PMC11290689; doi:10.1371/journal.pone.0306625)
Supplement: Attachment — Submitted filename: Response to Reviwers m.docx [file pone.0306625.s012.docx]

**Response to Authors**

Dear respected editorials and reviewers we have really appreciate for your rest less effort to amend this manuscript. By saying this, respected editorials by following the comment we have tried to adjust the manuscript in marked and unmarked format. So, respected editorials please publish on time as much as possible. And here we also put the general response to the author’s comment

| **Comments** | **Response** |
| --- | --- |
| **Reviewer 4** | **Authors** |
| I. The suggested title by reviewer was “**Effects of dry bio- slurry and nitrogen fertilizer on potato and wheat yields under rotation cropping system”** II. The abstract is not clear and precise. Please rewrite this?, III. The language is also still poor such as grammar, punctuations (line 79: (13) Due), spacing (line 27: 3.85tha-1).Line 14 and 106: The experiment was contain ten levels of treatments Control ---?.Line 21-22: the results were indicated that; an application of dry bio-slurry with nitrogen fertilizer was ---? - IV. Line 14-21: Write full description of all abbreviations mentioned first in the text: DBS, RN, RCBD, ANOVA, SAS. V. Most sentences are too long and difficult to understand for readers (Line 9-12, 93-96). So, make the sentences short and clear VI. Words found in the title cannot be a keyword like dry bio-slurry, potato, wheat VII. Line 96: Location of the study area (110 21' 22'' N and 370 25' 43'' E), is not correct. It is a point, not a polygon?Cgfc VIII. Amend citations based on the journal guidelines Bhjbh IX. Line 93-94: three sites (Y1S1, Y2S1and Y2S2), this not clearly described whether 2 or 3? X. Conclusion part is poor. So, rewrite this properly. | I. Based on your (reviewer 4) suggestion we have been adjust the title **“Effects of dry bio- slurry and nitrogen fertilizer on potato and wheat yields under rotation cropping system** II. Here we have wanted to thank you for reviewers for their rest less effort to make precise and clear the abstract part. By saying this respected reviewer, we are re adjust and write this abstract part in order to be clear and precise III. Based on the comment we correct the grammar, punctuations and spaces IV. Here in the lines, we make adjust it by full writing those abbreviations V. Here also We corrected these long sentences by making more sense full and short VI. Here we rewrite the words that are note in the manuscript title VII. In this based on the reviewer comment we correct it by putting polygon points (110 21' 18'' to 110 21' 22'' N and 370 25' 37'' to 370 25' 43'' E) rather single points VIII. Based on the comment and the guidelines of the journals we put the citations under closed bracts [ ] IX. We have adjusted by deleted these unclear abbreviations on the lines X. Her respected editorials based on your concrete comment we rewrite and correct it XI. |
| **General Comments** | **Response** |
| **Reviewer 4** | **Authors** |
| I. The authors addressed most of the comments raised in the previous version. However, I am not still satisfied with the introductory sentences in the abstract, which I tried to rephrase it in the document. Moreover, I request the authors as well as the editors to revisit the document towards grammar, sentence construction and typographical errors. It is one of the major problems of the document. In addition, the MRR values are not correctly calculated, which I also tried to correct in the document. I recommend accepting the document after incorporation of the comments and rigorous grammatical and typographical corrections. For more information, please refer the commented document. | I. Here we want to appreciate all of the reviewers including reviewer1 for their effort as manuscript be well edited by language writing. by fully considering and give attention for their frequent comment we have correct and check it by fluent speaker and language skilled persons in addition to our correction effort .so the manuscript well edited and corrected in terms of language. And in the case of MRR as you remember respected reviewer based on reviwer1 previous comment we have check the economic analysis by following CIMMYT guideline and we correct our recommendation from 50%RN+50%DBS to 25%DBS+75%RN. Here respected reviewers I assured to you we have re check it and correct it and it is true |
