# Supplementary material for: Effects of dry bio-slurry and nitrogen fertilizer on potato and wheat yields under rotation cropping system
Source: PLoS One. 2024 Jul 31;19(7):e0306625. doi: 10.1371/journal.pone.0306625 (PMC11290689; doi:10.1371/journal.pone.0306625)
Supplement: Attachment — Submitted filename: Review report PONE-D-23-10312R4.docx [file pone.0306625.s014.docx]

**Review report**

**Date: 4/06/2024**

**Manuscript number**: PONE-D-23-10312R4

**Title:** Effects of dry-bio-slurry with chemical nitrogen fertilizer on growth and yield of potato and wheat under short term crop rotation

**General comment**

The authors did not still address the MRR calculation correctly. Although the grammar, sentence construction and typographical errors are improved, there are still issues to be addressed in this regard. I still request the authors as well as the editors to take care of grammar and typographical errors.

I recommend the acceptance of the document after incorporation of the comments and rigorous grammatical and typographical corrections. For more information, please refer the commented document.
