# Supplementary material for: Effects of dry bio-slurry and nitrogen fertilizer on potato and wheat yields under rotation cropping system
Source: PLoS One. 2024 Jul 31;19(7):e0306625. doi: 10.1371/journal.pone.0306625 (PMC11290689; doi:10.1371/journal.pone.0306625)
Supplement: Attachment — Submitted filename: Response to Reviwers J.docx [file pone.0306625.s015.docx]

**Response to Authors**

Dear respected editorials and reviewers we have really appreciate for your rest less effort to amend this manuscript. By saying this, respected editorials by following the comment that was given in the previous and now round we have tried to adjust the manuscript in marked and unmarked format. So, respected editorials please publish on time as much as possible. And here we also put the response to the author’s comment

| **Comments** | **Response** |
| --- | --- |
| **Reviewer 1** | **Authors** |
| I. The authors did not still address the MRR calculation correctly. II. Although the grammar, sentence construction and typographical errors are improved, there are still issues to be addressed in this regard. I still request the authors as well as the editors to take care of grammar and typographical errors. | I. In this based on your previous comment we carefully recheck it and adjust based on your previous comment calculation method. Based on that the MRR values of both 50% RN+50% DBS (8461.1% to 3204.7%) and 25%DBS+75%RN (4486.3%to4463.3%) undominated treatments values are changed. Here we want thank you for your repeated comment that helps as to see correctly. And the recommendation lies on 25%DBS+75%RN than 50%RN+50%DBS II. Here we want to appreciate all of the reviewers for their effort as manuscript be well edited by language writing. By considering and giving full attention for their frequent comment we have correct and check it by fluent speaker and language skilled persons in addition to our correction effort. So, the manuscript improved in terms of language. And also we correct the grammar, punctuations and spaces again III. |
| **General Comments** | **Response** |
| **Journal Requirements** | **Authors** |
| I. Please review your reference list to ensure that it is complete and correct. If you have cited papers that have been retracted, please include the rationale for doing so in the manuscript text, or remove these references and replace them with relevant current references. Any changes to the reference list should be mentioned in the rebuttal letter that accompanies your revised manuscript. If you need to cite a retracted article, indicate the article’s retracted status in the References list and also include a citation and full reference for the retraction notice. | I. Her we also check all reference list that are corrected and completed rather retracted |
